# Supplementary material for: Research to evaluate safety and impact of long COVID intervention with Ensitrelvir for National Cohort (RESILIENCE Study): A protocol for a randomized, double-blind, placebo-controlled trial
Source: PLoS One. 2025 Nov 3;20(11):e0335609. doi: 10.1371/journal.pone.0335609 (PMC12582476; doi:10.1371/journal.pone.0335609)
Supplement: S1 File — The full study protocol in English. (DOCX) [file pone.0335609.s001.docx]

Specific Clinical Research Plan

Efficacy of Encitrelvir fumarate in the treatment of COVID-19 post-illness symptoms.

Research Evaluation of Safety and Impact in Long COVID Intervention with Ensitrelvir for National Cohort

(RESILIENCE STUDY)

| Principal investigator | Satoshi Kutsuna  Department of Infection Control, The University of Osaka﻿ Hospital  TEL: 06-6879-5111 |
| --- | --- |
| Research Plan Number | COVID-ENT-001 |
| creation-day | July 25, 2025 |
| version number | Version 4.0 |

version management

| version number | Date Created/Revised |
| --- | --- |
| 1st ed. | November 13, 2023 |
| Version 1.1 | December 28, 2023 |
| Version 2.0 | May 21, 2024 |
| Version 2.1 | July 31, 2024 |
| Version 2.2 | August 9, 2024 |
| Version 2.3 | October 28, 2024 |
| Version 2.4 | November 21, 2024 |
| Version 3.0 | November 26, 2024 |
| Version 3.1 | January 21, 2025 |
| Version 3.2 | April 28, 2025 |
| Version 3.3 | June 2, 2025 |
| Version 4.0 | July 25, 2025 |

**summary**

1. **Purpose and Contents of Clinical Research**

| Purpose of the Study | To verify the superiority of ensitrelvir over placebo for post-COVID-19 conditions in mild COVID-19 patients treated with encitrelvir fumarate for 5 days. The safety of encitrelvir will also be evaluated. |
| --- | --- |
| Planned Research Period | jRCT publication date～ December 31, 2026 |
| Number of planned research subjects | Total of 2,000 cases as the number of study subjects to be enrolled  Encitrelvir group: 1,000 cases  Placebo group: 1,000 cases |
| Study Design | Multicenter, randomized, double-blind, parallel-group study |
| target disease name | COVID-19 |
| criterion (criteria) for selection | (1) Persons diagnosed with COVID-19 by nucleic acid amplification (PCR, LAMP method, etc.), antigen qualitative test or antigen quantitative test at the medical institution referring the research subject  (2) Persons with a mild disease severity classification in the Guide to the Clinical Practice of New-type Coronavirus Infections (COVID-19) (Edition 10.0) at the time of obtaining consent.  (3) Those who are expected to start taking the study drug within 72 hours of the onset of the disease.  (4) Persons with a body temperature of 37.0°C or higher at the time of definitive diagnosis at the referring medical institution for the research subject  (5) Women of childbearing potential who are able to use adequate contraception with their partner when having sexual intercourse during the period of taking the study drug and within 2 weeks after the last dose.  (6) Persons who are 18 years of age or older at the time of obtaining consent  (7) Persons who can obtain written consent from the individual to participate in this study. |
| exclusion criteria | (1) Persons at high risk for severe COVID-19  (2) Persons who have received antiviral drugs indicated for COVID-19 (e.g., remdesivir, molnupiravir, nilmatrelvir/ritonavir, encitrelvir fumarate) or neutralizing antibody drugs (caciribimab/imdevimab. etc.) since the onset of this COVID-19 or within 15 days prior to the date consent was obtained Sotrovimab, tixagevimab/silgavimab, etc.).  (3)  Have received anti-IL-6 antibody preparations (e.g., tocilizumab), JAK inhibitors (e.g., baricitinib; excluding topical use), or oral, suppository, or injectable steroids within 2 weeks prior to consent.  (4) Have participated in a clinical trial of a COVID-19 treatment since the onset of this COVID-19  (5) Those who are unable to meet online with the principal investigator or research assistant physicians using a smartphone  (6) Persons who are unable to enter information into the patient-reported outcomes collection system using a smartphone  (7) Patients with a history of hypersensitivity to any ingredient of encitrelvir products  (8) Patients who are receiving a drug that is contraindicated with encitrelvir fumarate or who are scheduled to receive the drug during the period of study drug administration and within 2 weeks after the last administration  (9) Patients with renal or hepatic dysfunction who are receiving colchicine  (10) Women who are pregnant or may become pregnant (The possibility of pregnancy shall be determined after checking menstrual history and recent sexual activity.)  (11) Lactating women  (12) Persons with severe hepatic dysfunction  (13) Immunocompromised patients and those on dialysis  (14) Persons with other coexisting infectious diseases  (15) Persons who are currently participating in another intervention study or who have participated in this study  (16) Have participated in a clinical trial of a pharmaceutical product within the past year  (17) Persons deemed inappropriate as research subjects by the principal investigator or subinvestigator for other reasons |
| protocol treatment | 1. Ensitrelvir group  Encytrelvir is administered orally at a dose of 375 mg on day 1 and 125 mg once daily on days 2 through 5.  2. placebo group  Placebo is administered orally at a dose of 3 tablets on day 1 and 1 tablet once daily on days 2 through 5. |
| Criteria for discontinuance | 1. discontinuation of study medication for each study subject  (1) When an adverse event occurs and the principal investigator or subinvestigator determines that the continuation of this research poses an unacceptable risk to the health of the research subjects  (2) If the research subject requests to stop taking the study drug  (3) If administration of a drug contraindicated with encitrelvir fumarate becomes necessary  (4) When other COVID-19 therapies or respiratory therapies are required, or when a co-morbid condition necessitates the administration of a COVID-19 therapy that is also indicated for the disease in question.  (5) If found to be inappropriate as a research subject  (6) When a female research subject is found to be pregnant  (7) Other cases in which the principal investigator or subinvestigator determines that the intervention should be discontinued  2. discontinuation of research for each research subject  (1) When the research subject requests discontinuation of the research  (2) If it proves impossible to conduct the required observation in the future due to the convenience of the research subject, such as a malfunction of the smartphone.  (3) If the research subject is found to have contracted COVID-19 again during the follow-up period  (4) If the subject is found to be inappropriate as a research subject before taking the study drug  (5) Other cases in which the principal investigator or subinvestigator determines that the research should be terminated  3. discontinuation of the entire study  (1) When new significant information is obtained that may adversely affect the safety of research subjects or the conduct of this research, such as when anticipated adverse events (e.g., illness) significantly exceed those anticipated at the time of planning.  (2) When it is judged to be extremely difficult to achieve the target number of research subjects, such as when the enrollment of research subjects is significantly slower than planned.  (3) If an opinion is received from an accredited clinical research review committee that this research should be discontinued.  (4) Other circumstances necessitating the discontinuation or suspension of this research |
| Efficacy Primary Endpoint | The proportion of subjects who have at least one of the following symptoms: malaise (fatigue), shortness of breath or dyspnea, abnormal sense of smell, or abnormal sense of taste at both 1 month and 3 months after the start of treatment, OR who have at least one of the following symptoms at 3 months: decreased concentration/thinking ability, decreased problem-solving ability, or memory loss (short- or long-term). |
| Efficacy secondary endpoints | (1) The proportion of subjects who have at least one of the following symptoms: malaise (fatigue), shortness of breath or difficulty breathing, abnormal sense of smell, or abnormal sense of taste at both 1 month and 3 months after the start of treatment, OR who have at least one of the following symptoms at 3 months: decreased concentration/thinking ability, decreased problem-solving ability, or memory loss (short- or long-term), with the symptoms being considered related or of unknown relation to COVID-19.  (2) The proportion of subjects who have at least one of the following symptoms: malaise (fatigue), shortness of breath or difficulty breathing, abnormal sense of smell, or abnormal sense of taste at all three time points (1, 3, and 6 months) after the start of treatment, OR who have at least one of the following symptoms at 6 months: decreased concentration/thinking ability, decreased problem-solving ability, or memory loss (short- or long-term).  (3) The proportion of subjects who have at least one of the following symptoms: malaise (fatigue), shortness of breath or difficulty breathing, abnormal sense of smell, or abnormal sense of taste at all three time points (1, 3, and 6 months) after the start of treatment, OR who have at least one of the following symptoms at 6 months: decreased concentration/thinking ability, decreased problem-solving ability, or memory loss (short- or long-term), with the symptoms being considered related or of unknown relation to COVID-19.  (4) The proportion of subjects who have at least one of the following symptoms: malaise (fatigue), shortness of breath or difficulty breathing, abnormal sense of smell, or abnormal sense of taste at both 1 month and 3 months after the start of treatment.  (5) The proportion of subjects who have at least one of the following symptoms: malaise (fatigue), shortness of breath or difficulty breathing, abnormal sense of smell, or abnormal sense of taste at both 1 month and 3 months after the start of treatment, with the symptoms being considered related or of unknown relation to COVID-19.  (6) The proportion of subjects who have each of the following symptoms: malaise (fatigue), shortness of breath or difficulty breathing, abnormal sense of smell, and abnormal sense of taste at both 1 month and 3 months after the start of treatment.  (7) The proportion of subjects who have each of the following symptoms: malaise (fatigue), shortness of breath or difficulty breathing, abnormal sense of smell, and abnormal sense of taste at both 1 month and 3 months after the start of treatment, with the symptoms being considered related or of unknown relation to COVID-19.  (8) The proportion of subjects who have at least one of the following symptoms at 3 months after the start of treatment: decreased concentration/thinking ability, decreased problem-solving ability, or memory loss (short- or long-term).  (9) The proportion of subjects who have at least one of the following symptoms at 3 months after the start of treatment: decreased concentration/thinking ability, decreased problem-solving ability, or memory loss (short- or long-term), with the symptoms being considered related or of unknown relation to COVID-19.  (10) The proportion of subjects who have each of the following symptoms at 3 months after the start of treatment: decreased concentration/thinking ability, decreased problem-solving ability, and memory loss (short- or long-term).  (11) The proportion of subjects who have each of the following symptoms at 3 months after the start of treatment: decreased concentration/thinking ability, decreased problem-solving ability, and memory loss (short- or long-term), with the symptoms being considered related or of unknown relation to COVID-19.  (12) The proportion of subjects who have not returned to their usual state of health as before COVID-19 at 3 months after the start of treatment and have at least one of the 14 COVID-19 symptoms (malaise (fatigue), body aches or muscle pain, headache, chills, feverishness, runny or stuffy nose, sore throat, cough, shortness of breath or difficulty breathing, nausea, vomiting, diarrhea, abnormal sense of smell, or abnormal sense of taste).  (13) The proportion of subjects who have not returned to their usual state of health as before COVID-19 at 3 months after the start of treatment and have at least one of the 4 neurological symptoms (decreased concentration/thinking ability, decreased problem-solving ability, memory loss (short- or long-term), or insomnia).  (14) The proportion of subjects who have not returned to their usual state of health as before COVID-19 at 3 months after the start of treatment and have at least one of the following symptoms: malaise (fatigue), body aches or muscle pain, headache, chills, feverishness, runny or stuffy nose, sore throat, cough, shortness of breath or difficulty breathing, nausea, vomiting, diarrhea, abnormal sense of smell, abnormal sense of taste, muscle weakness, decreased concentration/thinking ability, decreased problem-solving ability, memory loss (short- or long-term), insomnia, hair loss, palpitations or increased heart rate, joint pain, loss of appetite, dizziness or abnormal balance, chest pain, or skin rash. |
| Efficacy Exploratory Endpoints | (1)The proportion of subjects who have at least one of the following symptoms: malaise (fatigue), shortness of breath or difficulty breathing, abnormal sense of smell, or abnormal sense of taste for 2 consecutive time points (2 and 3 months) after the start of treatment, OR who have at least one of the following symptoms at 3 months: decreased concentration/thinking ability, decreased problem-solving ability, or memory loss (short- or long-term).  (2) The proportion of subjects who have at least one of the following symptoms: malaise (fatigue), shortness of breath or difficulty breathing, abnormal sense of smell, or abnormal sense of taste for 2 consecutive time points (2 and 3 months) after the start of treatment, OR who have at least one of the following symptoms at 3 months: decreased concentration/thinking ability, decreased problem-solving ability, or memory loss (short- or long-term), with the symptoms being considered related or of unknown relation to COVID-19.  (3) The proportion of subjects who have at least one of the following symptoms: malaise (fatigue), shortness of breath or difficulty breathing, abnormal sense of smell, or abnormal sense of taste for 2 consecutive time points (2 and 3 months) after the start of treatment.  (4) The proportion of subjects who have at least one of the following symptoms: malaise (fatigue), shortness of breath or difficulty breathing, abnormal sense of smell, or abnormal sense of taste for 2 consecutive time points (2 and 3 months) after the start of treatment, with the symptoms being considered related or of unknown relation to COVID-19.  (5) The proportion of subjects who have each of the following symptoms: malaise (fatigue), shortness of breath or difficulty breathing, abnormal sense of smell, and abnormal sense of taste for 2 consecutive time points (2 and 3 months) after the start of treatment.  (6) The proportion of subjects who have each of the following symptoms: malaise (fatigue), shortness of breath or difficulty breathing, abnormal sense of smell, and abnormal sense of taste for 2 consecutive time points (2 and 3 months) after the start of treatment, with the symptoms being considered related or of unknown relation to COVID-19.  (7) The proportion of subjects who have at least one of the 14 COVID-19 symptoms (malaise (fatigue), body aches or muscle pain, headache, chills, feverishness, runny or stuffy nose, sore throat, cough, shortness of breath or difficulty breathing, nausea, vomiting, diarrhea, abnormal sense of smell, or abnormal sense of taste) at both 1 month and 3 months after the start of treatment, OR who have at least one of the following symptoms at 3 months: muscle weakness, decreased concentration/thinking ability, decreased problem-solving ability, memory loss (short- or long-term), insomnia, hair loss, palpitations or increased heart rate, joint pain, loss of appetite, dizziness or abnormal balance, chest pain, or skin rash.  (8) The proportion of subjects who have at least one of the 14 COVID-19 symptoms (malaise (fatigue), body aches or muscle pain, headache, chills, feverishness, runny or stuffy nose, sore throat, cough, shortness of breath or difficulty breathing, nausea, vomiting, diarrhea, abnormal sense of smell, or abnormal sense of taste) at both 1 month and 3 months after the start of treatment, OR who have at least one of the following symptoms at 3 months: muscle weakness, decreased concentration/thinking ability, decreased problem-solving ability, memory loss (short- or long-term), insomnia, hair loss, palpitations or increased heart rate, joint pain, loss of appetite, dizziness or abnormal balance, chest pain, or skin rash, with the symptoms being considered related or of unknown relation to COVID-19.  (9) The proportion of subjects who have at least one of the 14 COVID-19 symptoms (malaise (fatigue), body aches or muscle pain, headache, chills, feverishness, runny or stuffy nose, sore throat, cough, shortness of breath or difficulty breathing, nausea, vomiting, diarrhea, abnormal sense of smell, or abnormal sense of taste) at both 1 month and 3 months after the start of treatment.  (10) The proportion of subjects who have at least one of the 14 COVID-19 symptoms (malaise (fatigue), body aches or muscle pain, headache, chills, feverishness, runny or stuffy nose, sore throat, cough, shortness of breath or difficulty breathing, nausea, vomiting, diarrhea, abnormal sense of smell, or abnormal sense of taste) at both 1 month and 3 months after the start of treatment, with the symptoms being considered related or of unknown relation to COVID-19.  (11) The proportion of subjects who have each of the 14 COVID-19 symptoms (malaise (fatigue), body aches or muscle pain, headache, chills, feverishness, runny or stuffy nose, sore throat, cough, shortness of breath or difficulty breathing, nausea, vomiting, diarrhea, abnormal sense of smell, or abnormal sense of taste) at both 1 month and 3 months after the start of treatment.  (12) The proportion of subjects who have each of the 14 COVID-19 symptoms (malaise (fatigue), body aches or muscle pain, headache, chills, feverishness, runny or stuffy nose, sore throat, cough, shortness of breath or difficulty breathing, nausea, vomiting, diarrhea, abnormal sense of smell, or abnormal sense of taste) at both 1 month and 3 months after the start of treatment, with the symptoms being considered related or of unknown relation to COVID-19.  (13) The proportion of subjects who have at least one of the following symptoms at 3 months after the start of treatment: muscle weakness, decreased concentration/thinking ability, decreased problem-solving ability, memory loss (short- or long-term), insomnia, hair loss, palpitations or increased heart rate, joint pain, loss of appetite, dizziness or abnormal balance, chest pain, or skin rash.  (14) The proportion of subjects who have at least one of the following symptoms at 3 months after the start of treatment: muscle weakness, decreased concentration/thinking ability, decreased problem-solving ability, memory loss (short- or long-term), insomnia, hair loss, palpitations or increased heart rate, joint pain, loss of appetite, dizziness or abnormal balance, chest pain, or skin rash, with the symptoms being considered related or of unknown relation to COVID-19.  (15) The proportion of subjects who have each of the following symptoms at 3 months after the start of treatment: muscle weakness, decreased concentration/thinking ability, decreased problem-solving ability, memory loss (short- or long-term), insomnia, hair loss, palpitations or increased heart rate, joint pain, loss of appetite, dizziness or abnormal balance, chest pain, or skin rash.  (16) The proportion of subjects who have each of the following symptoms at 3 months after the start of treatment: muscle weakness, decreased concentration/thinking ability, decreased problem-solving ability, memory loss (short- or long-term), insomnia, hair loss, palpitations or increased heart rate, joint pain, loss of appetite, dizziness or abnormal balance, chest pain, or skin rash, with the symptoms being considered related or of unknown relation to COVID-19.  (17) The proportion of subjects who have not returned to their usual state of health as before COVID-19 at 3 months after the start of treatment.  (18) The amount of change from baseline in QOL at 3 months and 6 months after the start of treatment, respectively.  (19) The amount of change from baseline in work productivity at 3 months and 6 months after the start of treatment, respectively. |
| Safety endpoints | (1) The number of cases, number of events, and their proportion of adverse events.  October 2023 (2) The number of cases, number of events, and their proportion of serious adverse events. |

1. **Schematic of the study (Schema)**

After 6 months

↓

Day 5 of dose

↓

Start taking

↓

Confirmation of post-illness symptoms

allocation

Encitrelvir fumarate group

Confirmation of post-illness symptoms

placebo

1. **Schedule for observation, examination, and evaluation**

The principal investigator or research associate will collect data according to the "Observation, Testing, and Evaluation Schedule.

|  | pre-contact period | curative phase | | Tracking period | | | | | | | |
| --- | --- | --- | --- | --- | --- | --- | --- | --- | --- | --- | --- |
|  | at registration | Test Drugs  Dose  Start Date | After 1 week^*2^ | | After 1 month | After 2 months | After 3 months | After 4 months | After 5 months | After 6 months | time of discontinuance |
| Visit | 1 | - | 2 | | 3 | 4 | 5 | 6 | 7 | 8 |  |
| Day |  | 0 | 7 | | 28 | 56 | 84 | 112 | 140 | 168 |  |
| tolerance level | -^*1^ | - | -2 to +5 | | ±7 | ±7 | ±7 | ±7 | ±7 | ±7 |  |
| Obtaining Consent | ● |  |  | |  |  |  |  |  |  |  |
| Registration and allocation | ● |  |  | |  |  |  |  |  |  |  |
| Background of Study Subjects | ● |  |  | |  |  |  |  |  |  |  |
| Height and Weight | ● |  |  | |  |  |  |  |  |  |  |
| co-morbid disease | ● |  |  | |  |  |  |  |  |  |  |
| pre-existing medical condition | ● |  |  | |  |  |  |  |  |  |  |
| Start taking study medication |  | ● |  | |  |  |  |  |  |  |  |
| record of test drug doses |  |  |  | |  |  |  |  |  |  |  |
| Online Interview | ● |  | ● | |  |  |  |  |  |  |  |
| Presence of risk factors for severe disease | ● |  |  | |  |  |  |  |  |  |  |
| Check concomitant medications | ● |  | ● | |  |  |  |  |  |  |  |
| Pregnancy Confirmation | ● |  |  | |  |  |  |  |  |  |  |
| post-affective symptoms |  |  |  | | ● | ● | ● | ● | ● | ● | period |
| quality of life |  | ● |  | | ● | ● | ● | ● | ● | ● | period |
| labor productivity |  | ● |  | | ● | ● | ● | ● | ● | ● | period |
| Medical visits and prescriptions for medications |  |  |  | | ● | ● | ● | ● | ● | ● | period |
| COVID-19 Re-incidence of COVID-19 |  |  |  | | ● | ● | ● | ● | ● | ● | period |
| Adverse events ^*3^ |  |  |  | |  |  |  |  |  |  |  |

●: Required items, 0: Items to be implemented whenever possible

*1: The study drug should be addressed so that it can be started within 72 hours of the onset of symptoms.

*2: Whenever possible, an online interview will be conducted when study medication or the study is discontinued.

*3: Collection of adverse events will begin on Day 0 and continue until 2 weeks after the last dose of study drug.

**Table of Contents**

[1. Definition of Abbreviations and Terms 1](#_Toc183598251)

[1.1. abbreviation 1](#_Toc183598252)

[1.2. definition of a word or phrase 1](#_Toc183598253)

[2. Research Background 2](#_Toc183598254)

[2.1. target disease name 2](#_Toc183598255)

[2.2. Concept of Target Diseases 2](#_Toc183598256)

[2.3. Epidemiology of target diseases 2](#_Toc183598257)

[2.4. standard treatment 2](#_Toc183598258)

[2.5. COVID-19 post-affect symptoms 3](#_Toc183598259)

[2.6. Drugs, etc. to be considered in this study 4](#_Toc183598260)

[2.7. Significance of conducting this study 4](#_Toc183598261)

[3. Purpose of the Study and Endpoints 5](#_Toc183598262)

[3.1. Purpose of the Study 5](#_Toc183598263)

[3.2. Efficacy Primary Endpoint 5](#_Toc183598264)

[3.3. Efficacy secondary endpoints 5](#_Toc183598265)

[3.4. Efficacy Exploratory Endpoints 6](#_Toc183598266)

[3.5. Safety endpoints 8](#_Toc183598267)

[4. Study Design 8](#_Toc183598268)

[4.1. Study Design 8](#_Toc183598269)

[4.2. Number of planned research subjects for this study 9](#_Toc183598270)

[4.3. Planned duration of this study 10](#_Toc183598271)

[5. Selection of research subjects 11](#_Toc183598272)

[5.1. criterion (criteria) for selection 11](#_Toc183598273)

[5.2. exclusion criteria 11](#_Toc183598274)

[6. Investigational Product 14](#_Toc183598275)

[6.1. Outline of the pharmaceuticals, etc. that are the subject of the research 14](#_Toc183598276)

[6.1.1. test drug 14](#_Toc183598277)

[6.1.1. control drug 14](#_Toc183598278)

[6.2. Ensuring the quality of pharmaceuticals and other products that are the subject of research 14](#_Toc183598279)

[7. Method of Administration (Protocol Treatment) 16](#_Toc183598280)

[7.1. Method of application of the drug or other product under study 16](#_Toc183598281)

[7.1.1. Encitrel Building Group 16](#_Toc183598282)

[7.1.2. Placebo group 16](#_Toc183598283)

[7.1.3. Basis for establishing the method of application of the drug or other product under study 16](#_Toc183598284)

[7.2. Concomitant medications/adjunctive therapy 16](#_Toc183598285)

[7.3. drug use prohibited in combination with other drugs 16](#_Toc183598286)

[8. Schedule of Assessments 18](#_Toc183598287)

[8.1. Observation, examination and evaluation schedule 18](#_Toc183598288)

[8.1.1. At registration (Visit 1) 18](#_Toc183598289)

[8.1.2. Start date of study medication 18](#_Toc183598290)

[8.1.3. After 1 week (Visit 2) 18](#_Toc183598291)

[8.1.4. After 1 month (Visit 3), 2 months (Visit 4), 3 months (Visit 5), 4 months (Visit 6), 5 months (Visit 7), 6 months (Visit 8) 18](#_Toc183598292)

[8.1.5. At discontinuation of follow-up period 18](#_Toc183598293)

[8.2. Study Calendar 19](#_Toc183598294)

[9. Observation, examination and evaluation procedures 20](#_Toc183598295)

[9.1. Decentralized Clinical Trials support system used in this study 20](#_Toc183598296)

[9.2. Recruitment of research subjects 20](#_Toc183598297)

[9.3. Research Candidate Registration 20](#_Toc183598298)

[9.4. Obtaining Consent 20](#_Toc183598299)

[9.5. Registration, allocation and sending of study drugs 20](#_Toc183598300)

[9.5.1. Registration, allocation and study drug delivery procedures 20](#_Toc183598301)

[9.5.2. Creation and storage of allocation procedures 21](#_Toc183598302)

[9.5.3. emergency key opening 21](#_Toc183598303)

[9.6. record of test drug doses 21](#_Toc183598304)

[9.7. Background of Study Subjects 21](#_Toc183598305)

[9.8. Height and Weight 22](#_Toc183598306)

[9.9. co-morbid disease 22](#_Toc183598307)

[9.10. pre-existing medical condition 22](#_Toc183598308)

[9.11. Presence of risk factors for severe disease 22](#_Toc183598309)

[9.12. Check concomitant medications 22](#_Toc183598310)

[9.13. Pregnancy 22](#_Toc183598311)

[9.14. post-affective symptoms 22](#_Toc183598312)

[9.15. quality of life 22](#_Toc183598313)

[9.16. labor productivity 22](#_Toc183598314)

[9.17. Medical visits and prescriptions for medications 22](#_Toc183598315)

[9.18. COVID-19 Re-incidence of COVID-19 23](#_Toc183598316)

[9.19. Criteria for Discontinuation and Termination by Study Subject 23](#_Toc183598317)

[9.19.1. Discontinuation per research subject 23](#_Toc183598318)

[9.19.2. Discontinuation procedures for each research subject 23](#_Toc183598319)

[9.19.3. Termination per research subject 24](#_Toc183598320)

[10. adverse event 25](#_Toc183598321)

[10.1. Definition of adverse events 25](#_Toc183598322)

[10.2. Collection period for adverse events 25](#_Toc183598323)

[10.3. Confirmation of subjective symptoms 25](#_Toc183598324)

[10.4. Adverse Event Assessment 25](#_Toc183598325)

[10.4.1. Adverse event name 25](#_Toc183598326)

[10.4.2. expression date 25](#_Toc183598327)

[10.4.3. severity (of an illness) 26](#_Toc183598328)

[10.4.4. seriousness 26](#_Toc183598329)

[10.4.5. Causal relationship with research 26](#_Toc183598330)

[10.4.6. Causal relationship with study drug 27](#_Toc183598331)

[10.4.7. day on which judgment and haste are avoided (judgement) 27](#_Toc183598332)

[10.4.8. return to origin 27](#_Toc183598333)

[10.5. Measures to be taken in case of adverse events 28](#_Toc183598334)

[10.5.1. Treatment of research subjects 28](#_Toc183598335)

[10.5.2. Adverse event follow-up 28](#_Toc183598336)

[10.5.3. Reporting to funding companies 28](#_Toc183598337)

[10.6. Adverse events expected in this study 28](#_Toc183598338)

[11. Reporting of Diseases and Other Safety Events. 30](#_Toc183598339)

[11.1. Definition of Disease, etc. 30](#_Toc183598340)

[11.2. Procedures for Reporting Serious Reporting of Diseases and Other Safety Events. to an Accredited Clinical Research Review Committee 30](#_Toc183598341)

[11.3. Procedures for reporting serious illnesses, etc. to the Minister of Health, Labour and Welfare 31](#_Toc183598342)

[12. data management 32](#_Toc183598343)

[12.1. Data Management Plan 32](#_Toc183598344)

[12.2. case report 32](#_Toc183598345)

[13. Statistical Considerations 33](#_Toc183598346)

[13.1. Definition of analysis population 33](#_Toc183598347)

[13.2. Data Handling 33](#_Toc183598348)

[13.2.1. Handling of measured values outside the specified tolerance range 33](#_Toc183598349)

[13.2.2. Handling of missing values 33](#_Toc183598350)

[13.3. Analysis Method 33](#_Toc183598351)

[13.3.1. Background of Study Subjects 33](#_Toc183598352)

[13.3.2. Status of study medication 33](#_Toc183598353)

[13.3.3. concomitant medications 34](#_Toc183598354)

[13.3.4. Efficacy Primary Endpoint 34](#_Toc183598355)

[13.3.5. Efficacy secondary endpoints 34](#_Toc183598356)

[13.3.6. Efficacy Exploratory Endpoints 37](#_Toc183598357)

[13.3.7. Subgroup analysis for efficacy endpoints 40](#_Toc183598358)

[13.3.8. Safety endpoints 40](#_Toc183598359)

[13.4. Criteria for interim analysis and early discontinuation 40](#_Toc183598360)

[13.5. Change in statistical analysis plan 40](#_Toc183598361)

[14. Quality Control and Quality Assurance 41](#_Toc183598362)

[14.1. Quality Control Policy 41](#_Toc183598363)

[14.2. Quality Targets 41](#_Toc183598364)

[14.3. monitoring 41](#_Toc183598365)

[14.4. audit 41](#_Toc183598366)

[14.5. Response to investigations by regulatory authorities, etc. 41](#_Toc183598367)

[14.6. incompatible 42](#_Toc183598368)

[14.6.1. Definition of Nonconformity 42](#_Toc183598369)

[14.6.2. Critical Nonconformity 42](#_Toc183598370)

[14.6.3. Nonconformity Management Procedures 42](#_Toc183598371)

[15. ethical consideration 43](#_Toc183598372)

[15.1. Rules and Regulations to be complied with 43](#_Toc183598373)

[15.2. Approval by an accredited clinical research review committee and the administrator of the implementing medical institution 43](#_Toc183598374)

[15.3. Cost sharing for research subjects in this study 43](#_Toc183598375)

[15.4. Consent Explanatory Document and Consent of Study Subjects 43](#_Toc183598376)

[15.4.1. Procedure for Obtaining Consent 43](#_Toc183598377)

[15.4.2. Items to be included in the consent document 44](#_Toc183598378)

[15.5. Consultation service from research subjects 45](#_Toc183598379)

[15.6. Anticipated benefits and disadvantages to the research subjects 45](#_Toc183598380)

[15.6.1. Projected Profit 45](#_Toc183598381)

[15.6.2. Anticipated disadvantages 45](#_Toc183598382)

[15.6.3. Comprehensive evaluation of benefits and disadvantages and measures to minimize disadvantages 45](#_Toc183598383)

[15.7. Confidentiality of research subjects (protection of personal information) 45](#_Toc183598384)

[16. Compensation for Study-Related Injury 47](#_Toc183598385)

[17. Discontinuation or termination of the entire clinical research 48](#_Toc183598386)

[17.1. Criteria for discontinuation 48](#_Toc183598387)

[17.2. Abort Procedure 48](#_Toc183598388)

[17.3. Termination Criteria 48](#_Toc183598389)

[18. Disclosure of research information and publication of results 49](#_Toc183598390)

[18.1. Register your study 49](#_Toc183598391)

[18.2. Publication of Research Results 49](#_Toc183598392)

[18.2.1. Key Evaluators Report 49](#_Toc183598393)

[18.2.2. general report 49](#_Toc183598394)

[18.2.3. Publication of conferences, etc. 49](#_Toc183598395)

[19. Change Management 51](#_Toc183598396)

[19.1. Changes to documents approved by an accredited clinical research review committee 51](#_Toc183598397)

[19.2. Change of implementation plan 51](#_Toc183598398)

[19.3. Minor changes to the implementation plan 51](#_Toc183598399)

[20. conflict of interest 52](#_Toc183598400)

[20.1. Sources of funding for this study 52](#_Toc183598401)

[20.2. conflict of interest management 52](#_Toc183598402)

[21. Periodic reports to the Accredited Clinical Research Review Committee and the Minister of Health, Labour and Welfare 53](#_Toc183598403)

[21.1. Periodic reports to the Accredited Clinical Research Review Board 53](#_Toc183598404)

[21.1.1. Matters to be reported in periodic reports 53](#_Toc183598405)

[21.1.2. Periodic Reporting Period 53](#_Toc183598406)

[21.2. Periodic reports to the Minister of Health, Labor and Welfare 53](#_Toc183598407)

[21.2.1. Matters to be reported in periodic reports 53](#_Toc183598408)

[21.2.2. Periodic Reporting Period 53](#_Toc183598409)

[22. Methods of storage and disposal of materials, records, etc. 54](#_Toc183598410)

[22.1. Retention of original documents 54](#_Toc183598411)

[22.2. Retention of documents of record as required by law 54](#_Toc183598412)

[22.3. Secondary Use of Information 55](#_Toc183598413)

[22.4. Disposal Procedures and Methods 55](#_Toc183598414)

[23. Ownership of Data and Results 56](#_Toc183598415)

[24. Committees, etc. to be established in this study 57](#_Toc183598416)

[24.1. Independent Data Monitoring Committee 57](#_Toc183598417)

[25. References 62](#_Toc183598431)

1. Definition of Abbreviations and Terms
   1. abbreviation

| abbreviation | perfect type | Japanese notation |
| --- | --- | --- |
| COVID-19 | Coronavirus Disease 2019 | new strains of coronavirus infection |
| SARS-CoV-2 | Severe acute respiratory syndrome coronavirus 2 | new strains of coronavirus |

- 1. definition of a word or phrase

1. principal investigator

A person who conducts clinical research as stipulated in the Clinical Research Act, which means a physician who supervises operations related to clinical research at a medical institution.

1. Principal investigator

In the case of multicenter collaborative research, a principal investigator who represents the principal investigators at multiple institutions.

1. research physician

A physician who shares clinical research duties under the guidance of a principal investigator at a medical institution.

1. monitoring

(2) "Investigation" means an investigation that the principal investigator designates a specific person to conduct regarding the progress of this research and whether or not this research is being conducted in accordance with the Clinical Research Act, its enforcement regulations, and the research protocol, in order to ensure that the clinical research is being conducted properly from the perspective of ensuring reliability of the clinical research and protecting the research subjects. (2) "Investigation" means an investigation conducted by the principal investigator by designating a specific person.

1. audit

An investigation that the principal investigator designates a specific person to conduct to determine whether or not this research was conducted in accordance with the Clinical Research Act, its enforcement regulations, and the research protocol, in order to ensure the reliability of the data collected through clinical research from the perspective of ensuring reliability in clinical research and protecting research subjects.

1. research collaborator

(2) "Research physician" means a pharmacist, nurse, or other medical personnel who cooperates in the research work of a principal investigator or subinvestigator under the guidance of the principal investigator or subinvestigator at the implementing medical institution.

1. Research subject referral medical institution

Medical institutions that refer patients who are candidates for research subjects to the implementing medical institution

1. Research Background
   1. target disease name

New coronavirus infection (COVID-19)

- 1. Concept of Target Diseases

COVID-19 is a respiratory infection caused by a novel coronavirus (SARS-CoV-2). The COVID-19 Clinical Practice Guide, Version 10.0 defines the following severity categories: mild, moderate I, moderate II, and severe. Mild, Moderate I, Moderate II, and Severe.


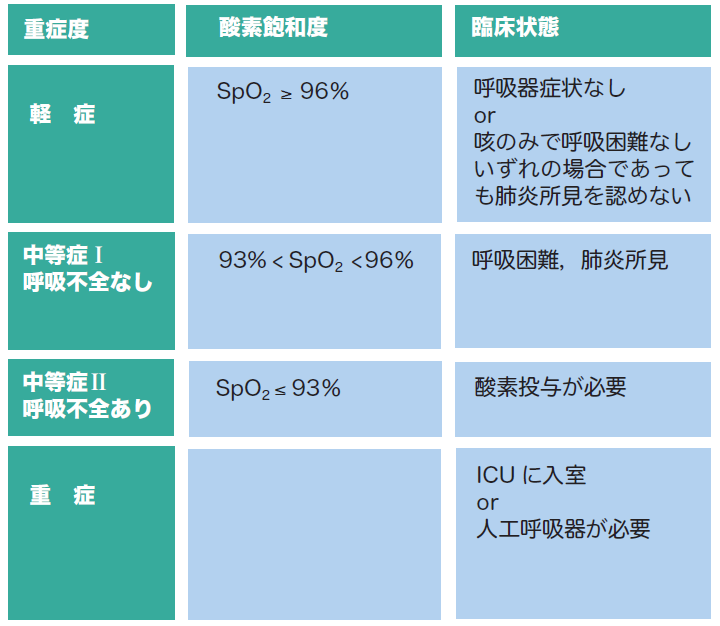


- 1. Epidemiology of target diseases

As of May 9, 2023, the cumulative number of COVID-19 cases in Japan was 33,803,572 (according to the Ministry of Health, Labor and Welfare). The early stages of the epidemic were characterized by the small number of children infected, but since the appearance of the mutant strain, infection among children has become more common. Men are more susceptible to severe cases than women, and the elderly are more susceptible than the young, but there is no difference in the risk of morbidity.

- 1. standard treatment

As of August 22, 2023, the following drugs are approved in Japan for the treatment of COVID-19.

| antiviral drug | Lemdecivir (Beckley), mornupiravir (Ragebrio), nilmatrelvir/ritonavir (Pakilovid), encitrelvir fumarate (Zocova) |
| --- | --- |
| neutralizing antibody | Casilivimab/imdemimab (Lonapreve), sotrovimab (Zebedee) tixagevimab/silgavimab (Evasheld) |
| Anti-inflammatory and immunomodulatory drugs | Dexamethasone (Dexate, etc.), baricitinib (Olmiento), tocilizumab (Actemra) |

Generic name (product name)

The COVID-19 Clinical Practice Guide, Version 10.0 recommends the use of antivirals and neutralizing antibody drugs for mild and moderate I cases, and dexamethasone, tocilizumab, and baricitinib for moderate II and above.

Symptomatic treatment is the usual treatment for patients with mild disease, the subject of this study. Nilmatrelvir/ritonavir, remdesivir, and mornupiravir may be used in elderly patients and those at high risk of severe disease with underlying medical conditions; as of December 2023, there is no evidence that administration of encitrelvir fumarate can reduce severe disease, but it has been shown to reduce the It is used in patients with mild disease who are at low risk for severe disease because of its ability to shorten the symptomatic period by approximately 1 day in patients with mild disease.

- 1. COVID-19 post-affect symptoms

COVID-19 post-associated symptoms are all symptoms that have persisted since the acute phase of the disease without any other apparent cause, despite the disappearance of infectiousness, or that arise anew or reappear during the course of the disease and persist. Typical symptoms are fatigue, joint pain, myalgia, cough, sputum production, shortness of breath, chest pain, hair loss, memory impairment, poor concentration, headache, depression, olfactory disturbance, taste disturbance, palpitations, diarrhea, abdominal pain, sleep disturbance, and muscle weakness.

The pathogenetic mechanisms of post-affection symptoms are still unclear and include direct damage to virus-infected tissues (especially the lungs), persistent infection with minute amounts of virus, progressive inflammation due to immune dysregulation after viral infection, vascular damage and ischemia due to increased blood coagulation and thrombosis caused by the virus, and dysregulation of the renin-angiotensin system caused by viral infection. Dysregulation of the renin-angiotensin system due to viral infections, etc. It is believed that in some cases, a combination of these factors, rather than a single pathogenesis, may be present as post-affection symptoms.

In a follow-up study in Japan of 1,066 patients with a diagnosis of COVID-19 and a history of hospitalization, approximately 30% of all affected patients had one or more post-affective symptoms even 12 months after diagnosis.[1] In a study of 1,003 patients with moderate disease or higher, symptoms such as muscle weakness and fatigue decreased in frequency over time, but were still present in approximately 5% to 10% of patients at 12 months, and some post-affective symptoms were still present in 13.6% of patients. [2] In an observational study of post-illness symptoms conducted jointly by The University of Osaka﻿ and Toyonaka City (about 5% of patients with moderate II or severe disease), 47.7% of 4,047 patients who responded to a questionnaire experienced some post-illness symptoms, with 5.2% of patients reporting symptoms at 30 days and 3.7% at 60 days after onset, including fatigue, cough, and hair loss 5.2% reported some post-illness symptoms at 30 days and 3.7% at 60 days, including fatigue, cough, and hair loss.[3]

A survey of antinucleocapsid antibody positivity conducted in 13,121 people aged 16-69 who donated blood at the Japanese Red Cross Society in February 2023 showed that more than 40% of them may be infected with SARS-CoV-2.[4] While it is possible for anyone to be infected at any time, the fact that no prevention methods for post-affection symptoms However, there are no established methods for prevention or treatment of post-exposure symptoms, which is a major challenge.

A study using health insurance data found that patients who took nilmatrelvir/ritonavir, one of the standard drugs for COVID-19, within 5 days of onset of illness had a 26% reduced risk of post-acute symptoms compared to patients who did not take the drug. [5] The study suggests that acute antiviral therapy may reduce the risk of post-embolic symptoms. However, nilmatrelvir/ritonavir is indicated only for patients at risk for severe disease, which limits the target population for administration.

- 1. Drugs, etc. to be considered in this study

Encitrelvir fumarate is a drug for the treatment of infections caused by SARS-CoV-2, discovered by Shionogi & Co. The drug exerts its antiviral effect against SARS-CoV-2 by inhibiting 3C-like protease, which is essential for the processing of the polyprotein encoded by the SARS-CoV-2 gene and for virus replication.

In a placebo-controlled, randomized, double-blind, parallel-group study in COVID-19 patients aged ≥12 years and <70 years (Phase 3 part of the international Phase 2/3 study [T1221]), 375 mg of encitrelvir was orally administered once daily on Day 1 and 125 mg on Days 2 through 5. (662 Japanese), of whom 690 had COVID-19 symptoms onset and less than 72 hours before randomization, the median time to recovery of 5 symptoms (fatigue or tiredness, fever or fevers, runny or stuffy nose, sore throat, and cough) was 167.9 hours in the encitrelvir group and 192.9 hours in the placebo group. and 192.2 hours in the placebo group (p=0.0407). Adverse drug reactions occurred in 24.5% (148/604) of patients, the most common being a decrease in high-density lipoprotein (18.4%).

The drug was approved as an emergency drug in November 2022.

- 1. Significance of conducting this study

An exploratory evaluation of post-incidence symptoms in the clinical trial described above (subanalysis through day 169 after initiation of treatment in a population of patients with baseline scores above the median of 14 symptoms characteristic of COVID-19) showed that the proportion of patients who developed 14 symptoms was 27% in the placebo group and 15% in the encytrelvir group (relative (45% relative risk reduction), and 4 neurologic symptoms such as impaired concentration and insomnia occurred in 43% of patients on placebo and 29% of patients on encitrelvir (33% relative risk reduction). [6] However, since this is an exploratory evaluation rather than a primary endpoint, more validated study results are desired.

Encitrelvir fumarate is an antiviral drug that can be prescribed to patients who are not at risk for severe disease, and if more reliable study designs show that it is also effective in reducing the onset of post-embolic symptoms, it may bring us closer to solving the social problem of post-embolic symptoms.

1. Objectives of the study and evaluation items
   1. Purpose of the Study

To evaluate the superiority of encitrelvir over placebo in post morbidity symptoms in mild COVID-19 patients treated with encitrelvir fumarate for 5 days. The safety of encitrelvir will also be evaluated.

- 1. Efficacy primary endpoint

Percentage of study subjects who had symptoms of fatigue, shortness of breath or dyspnea, abnormal sense of smell, or abnormal taste at both 1 and 3 months after the start of treatment, or who had symptoms of poor concentration and thinking, poor problem solving, or forgetfulness (short-term or long-term) at 3 months.

Rationale for setting the primary endpoints

We limited our evaluation of post-affect symptoms to those symptoms that occur most frequently. The WHO definition of COVID-19 post-illness symptoms is "symptoms that persist for at least 2 months and cannot be explained by symptoms of other diseases, and are usually still present 3 months after the onset of COVID-19. Since COVID-19 post-illness symptoms are usually present as early as 3 months after the onset of COVID-19, we decided to evaluate post-illness symptoms at 3 months after the start of treatment, and we required that COVID-19 acute phase symptoms, which can also occur as symptoms of other diseases, be present at both 1 month and 3 months after the start of treatment. COVID-19 acute phase symptoms, which can also occur as symptoms of other diseases, were required to be present at both 1 and 3 months of treatment.

- 1. Efficacy secondary endpoints
  2. The proportion of subjects who have at least one of the following symptoms: malaise (fatigue), shortness of breath or dyspnea, abnormal sense of smell, or abnormal sense of taste at both 1 month and 3 months after the start of treatment, OR who have at least one of the following symptoms at 3 months: decreased concentration/thinking ability, decreased problem-solving ability, or memory loss (short- or long-term), (with the symptoms being considered related or of unknown relation to COVID-19).
  3. The proportion of subjects who have at least one of the following symptoms: malaise (fatigue), shortness of breath or dyspnea, abnormal sense of smell, or abnormal sense of taste at all three time points (1, 3, and 6 months) after the start of treatment, OR who have at least one of the following symptoms at 6 months: decreased concentration/thinking ability, decreased problem-solving ability, or memory loss (short- or long-term).
  4. The proportion of subjects who have at least one of the following symptoms: malaise (fatigue), shortness of breath or dyspnea, abnormal sense of smell, or abnormal sense of taste at all three time points (1, 3, and 6 months) after the start of treatment, OR who have at least one of the following symptoms at 6 months: decreased concentration/thinking ability, decreased problem-solving ability, or memory loss (short- or long-term), (with the symptoms being considered related or of unknown relation to COVID-19).
  5. The proportion of subjects who have at least one of the following symptoms: malaise (fatigue), shortness of breath or dyspnea, abnormal sense of smell, or abnormal sense of taste at both 1 month and 3 months after the start of treatment.
  6. The proportion of subjects who have at least one of the following symptoms: malaise (fatigue), shortness of breath or dyspnea, abnormal sense of smell, or abnormal sense of taste at both 1 month and 3 months after the start of treatment, (with the symptoms being considered related or of unknown relation to COVID-19).
  7. The proportion of subjects who have each of the following symptoms: malaise (fatigue), shortness of breath or dyspnea, abnormal sense of smell, and abnormal sense of taste at both 1 month and 3 months after the start of treatment.
  8. The proportion of subjects who have each of the following symptoms: malaise (fatigue), shortness of breath or dyspnea, abnormal sense of smell, and abnormal sense of taste at both 1 month and 3 months after the start of treatment, (with the symptoms being considered related or of unknown relation to COVID-19).
  9. The proportion of subjects who have at least one of the following symptoms at 3 months after the start of treatment: decreased concentration/thinking ability, decreased problem-solving ability, or memory loss (short- or long-term).
  10. The proportion of subjects who have at least one of the following symptoms at 3 months after the start of treatment: decreased concentration/thinking ability, decreased problem-solving ability, or memory loss (short- or long-term), (with the symptoms being considered related or of unknown relation to COVID-19).
  11. The proportion of subjects who have each of the following symptoms at 3 months after the start of treatment: decreased concentration/thinking ability, decreased problem-solving ability, and memory loss (short- or long-term).
  12. The proportion of subjects who have each of the following symptoms at 3 months after the start of treatment: decreased concentration/thinking ability, decreased problem-solving ability, and memory loss (short- or long-term), (with the symptoms being considered related or of unknown relation to COVID-19).
  13. The proportion of subjects who have not returned to their usual state of health as before COVID-19 at 3 months after the start of treatment and have at least one of the 14 COVID-19 symptoms (malaise (fatigue), body aches or muscle pain, headache, chills, feverishness, runny or stuffy nose, sore throat, cough, shortness of breath or dyspnea, nausea, vomiting, diarrhea, abnormal sense of smell, or abnormal sense of taste).
  14. The proportion of subjects who have not returned to their usual state of health as before COVID-19 at 3 months after the start of treatment and have at least one of the 4 neurological symptoms (decreased concentration/thinking ability, decreased problem-solving ability, memory loss (short- or long-term), or insomnia).
  15. The proportion of subjects who have not returned to their usual state of health as before COVID-19 at 3 months after the start of treatment and have at least one of the following symptoms: malaise (fatigue), body aches or muscle pain, headache, chills, feverishness, runny or stuffy nose, sore throat, cough, shortness of breath or dyspnea, nausea, vomiting, diarrhea, abnormal sense of smell, abnormal sense of taste, muscle weakness, decreased concentration/thinking ability, decreased problem-solving ability, memory loss (short- or long-term), insomnia, hair loss, palpitations or increased heart rate, joint pain, loss of appetite, dizziness or abnormal balance, chest pain, or skin rash.

[Rationale for Secondary Endpoints]

- 1. To evaluate the effect of ensitrelvir fumarate on post-COVID conditions that the subjects themselves judge to be related or of unknown relation to COVID-19, among the symptoms of the primary endpoint.
  2. To evaluate the effect of ensitrelvir fumarate on post-COVID conditions for a longer period than the time point set in the primary endpoint.
  3. Of the items in (2), to evaluate the effect of ensitrelvir fumarate on post-COVID conditions that the subjects themselves judge to be related or of unknown relation to COVID-19.
  4. To evaluate the effect of ensitrelvir fumarate on post-COVID conditions that have persisted since the acute phase.
  5. Of the items in (4), to evaluate the effect of ensitrelvir fumarate on post-COVID conditions that the subjects themselves judge to be related or of unknown relation to COVID-19.
  6. To evaluate the effect of ensitrelvir fumarate on each post-COVID symptom that has persisted since the acute phase.
  7. Of the items in (6), to evaluate the effect of ensitrelvir fumarate on each post-COVID symptom that the subjects themselves judge to be related or of unknown relation to COVID-19.
  8. To evaluate the effect of ensitrelvir fumarate on neurological post-COVID conditions.
  9. Of the items in (8), to evaluate the effect of ensitrelvir fumarate on neurological post-COVID conditions that the subjects themselves judge to be related or of unknown relation to COVID-19.
  10. To evaluate the effect of ensitrelvir fumarate on each neurological post-COVID symptom.
  11. Of the items in (10), to evaluate the effect of ensitrelvir fumarate on each neurological post-COVID symptom that the subjects themselves judge to be related or of unknown relation to COVID-19.
  12. To evaluate the effect of ensitrelvir fumarate on post-COVID conditions related to acute phase symptoms in patients who have not returned to their usual state of health.
  13. To evaluate the effect of ensitrelvir fumarate on neurological post-COVID conditions in patients who have not returned to their usual state of health.
  14. To evaluate the effect of ensitrelvir fumarate on overall post-COVID conditions in patients who have not returned to their usual state of health.
  15. Efficacy Exploratory Endpoints
  16. The proportion of subjects who have at least one of the following symptoms: malaise (fatigue), shortness of breath or dyspnea, abnormal sense of smell, or abnormal sense of taste for 2 consecutive time points (2 and 3 months) after the start of treatment, OR who have at least one of the following symptoms at 3 months: decreased concentration/thinking ability, decreased problem-solving ability, or memory loss (short- or long-term).
  17. The proportion of subjects who have at least one of the following symptoms: malaise (fatigue), shortness of breath or dyspnea, abnormal sense of smell, or abnormal sense of taste for 2 consecutive time points (2 and 3 months) after the start of treatment, OR who have at least one of the following symptoms at 3 months: decreased concentration/thinking ability, decreased problem-solving ability, or memory loss (short- or long-term), (with the symptoms being considered related or of unknown relation to COVID-19).
  18. The proportion of subjects who have at least one of the following symptoms: malaise (fatigue), shortness of breath or dyspnea, abnormal sense of smell, or abnormal sense of taste for 2 consecutive time points (2 and 3 months) after the start of treatment.
  19. The proportion of subjects who have at least one of the following symptoms: malaise (fatigue), shortness of breath or dyspnea, abnormal sense of smell, or abnormal sense of taste for 2 consecutive time points (2 and 3 months) after the start of treatment, (with the symptoms being considered related or of unknown relation to COVID-19).
  20. The proportion of subjects who have each of the following symptoms: malaise (fatigue), shortness of breath or dyspnea, abnormal sense of smell, and abnormal sense of taste for 2 consecutive time points (2 and 3 months) after the start of treatment.
  21. The proportion of subjects who have each of the following symptoms: malaise (fatigue), shortness of breath or dyspnea, abnormal sense of smell, and abnormal sense of taste for 2 consecutive time points (2 and 3 months) after the start of treatment, (with the symptoms being considered related or of unknown relation to COVID-19).
  22. The proportion of subjects who have at least one of the 14 COVID-19 symptoms (malaise (fatigue), body aches or muscle pain, headache, chills, feverishness, runny or stuffy nose, sore throat, cough, shortness of breath or dyspnea, nausea, vomiting, diarrhea, abnormal sense of smell, or abnormal sense of taste) at both 1 month and 3 months after the start of treatment, OR who have at least one of the following symptoms at 3 months: muscle weakness, decreased concentration/thinking ability, decreased problem-solving ability, memory loss (short- or long-term), insomnia, hair loss, palpitations or increased heart rate, joint pain, loss of appetite, dizziness or abnormal balance, chest pain, or skin rash.
  23. The proportion of subjects who have at least one of the 14 COVID-19 symptoms at both 1 month and 3 months after the start of treatment, OR who have at least one of the following symptoms at 3 months as listed in (7), (with the symptoms being considered related or of unknown relation to COVID-19).
  24. The proportion of subjects who have at least one of the 14 COVID-19 symptoms (as listed in (7)) at both 1 month and 3 months after the start of treatment.
  25. The proportion of subjects who have at least one of the 14 COVID-19 symptoms (as listed in (7)) at both 1 month and 3 months after the start of treatment, (with the symptoms being considered related or of unknown relation to COVID-19).
  26. The proportion of subjects who have each of the 14 COVID-19 symptoms (as listed in (7)) at both 1 month and 3 months after the start of treatment.
  27. The proportion of subjects who have each of the 14 COVID-19 symptoms (as listed in (7)) at both 1 month and 3 months after the start of treatment, (with the symptoms being
  28. The proportion of subjects who have at least one of the following symptoms at 3 months after the start of treatment: muscle weakness, decreased concentration/thinking ability, decreased problem-solving ability, memory loss (short- or long-term), insomnia, hair loss, palpitations or increased heart rate, joint pain, loss of appetite, dizziness or abnormal balance, chest pain, or skin rash.
  29. The proportion of subjects who have at least one of the symptoms listed in (13) at 3 months after the start of treatment, (with the symptoms being considered related or of unknown relation to COVID-19).
  30. The proportion of subjects who have each of the symptoms listed in (13) at 3 months after the start of treatment.
  31. The proportion of subjects who have each of the symptoms listed in (13) at 3 months after the start of treatment, (with the symptoms being considered related or of unknown relation to COVID-19).
  32. The proportion of subjects who have not returned to their usual state of health as before COVID-19 at 3 months after the start of treatment.
  33. The amount of change from baseline in QOL at 3 months and 6 months after the start of treatment, respectively.
  34. The amount of change from baseline in work productivity at 3 months and 6 months after the start of treatment, respectively.

[Rationale for Exploratory Endpoints]

1. To explore the effect of ensitrelvir fumarate on post-COVID conditions when the duration of symptom continuation is set to be shorter.
2. Of the items in (1), to evaluate the effect of ensitrelvir fumarate on post-COVID conditions that the subjects themselves judge to be related or of unknown relation to COVID-19.
3. To explore the effect of ensitrelvir fumarate on post-COVID conditions that have persisted since the acute phase, when the duration of symptom continuation is set to be shorter.
4. Of the items in (3), to evaluate the effect of ensitrelvir fumarate on post-COVID conditions that the subjects themselves judge to be related or of unknown relation to COVID-19.
5. To explore the effect of ensitrelvir fumarate on each post-COVID symptom that has persisted since the acute phase, when the duration of symptom continuation is set to be shorter.
6. Of the items in (5), to evaluate the effect of ensitrelvir fumarate on each post-COVID symptom that the subjects themselves judge to be related or of unknown relation to COVID-19.
7. To explore the effect of ensitrelvir fumarate on a wide range of post-COVID conditions.
8. Of the items in (7), to evaluate the effect of ensitrelvir fumarate on post-COVID conditions that the subjects themselves judge to be related or of unknown relation to COVID-19.
9. To explore the effect of ensitrelvir fumarate on post-COVID conditions associated with a wide range of acute-phase symptoms.
10. Of the items in (9), to evaluate the effect of ensitrelvir fumarate on post-COVID conditions that the subjects themselves judge to be related or of unknown relation to COVID-19.
11. To explore the effect of ensitrelvir fumarate on each post-COVID symptom associated with a wide range of acute-phase symptoms. (
12. Of the items in (11), to evaluate the effect of ensitrelvir fumarate on each post-COVID symptom that the subjects themselves judge to be related or of unknown relation to COVID-19.
13. To explore the effect of ensitrelvir fumarate on other wide-ranging post-COVID conditions.
14. Of the items in (13), to evaluate the effect of ensitrelvir fumarate on post-COVID conditions that the subjects themselves judge to be related or of unknown relation to COVID-19.
15. To explore the effect of ensitrelvir fumarate on each of the other wide-ranging post-COVID symptoms. (
16. Of the items in (15), to evaluate the effect of ensitrelvir fumarate on each post-COVID symptom that the subjects themselves judge to be related or of unknown relation to COVID-19.
17. To evaluate the effect of ensitrelvir fumarate in patients who have not returned to their usual state of health due to post-COVID conditions other than those evaluated in the study protocol.
18. To evaluate the effect of ensitrelvir fumarate on QOL.
19. To evaluate the effect of ensitrelvir fumarate on work productivity scores.
    1. Safety endpoints

(1) Number and rate of adverse events

(2) Number and rate of serious adverse events

Rationale for establishing safety endpoints

(1) (2) To evaluate the safety of Encitrelvir fumarate.

1. Study Design
   1. Study Design

A multicenter, randomized, double-blind, parallel-group study of the following two groups.

1. Encitrel Building Group
2. Placebo group

For allocation, a stratified replacement block method is applied with the following allocation factors

COVID-19 vaccination history

Severity of COVID-19 symptoms (14 symptoms) at the time of obtaining consent (<9 points, ≥9 points)

After 6 months

↓

Day 5 of dose

↓

Start taking

↓

Confirmation of post-illness symptoms

allocation

Encitrelvir fumarate group

Confirmation of post-illness symptoms

placebo

[Rationale for setting up the study design: ]

[6]Random assignment was used to minimize background bias and increase comparability between groups. In the exploratory analysis of the Phase 3 part of the international Phase 2/3 study [T1221 study], those with a baseline COVID-19 symptom severity* (14 symptoms) score of 9 or higher had a higher incidence of post-affect symptoms than those with a score of less than 9, so these were used as allocation factors. The primary endpoint is the subjective symptoms of the study subjects, and we will be blinded to the possibility that knowing the allocation group may affect the assessment.

The sum of the following symptoms scored on a 4-point scale (0: no symptoms, 1: mild, 2: moderate, 3: severe): fatigue, muscle aches or pains, headache, chills/sweats, fever, runny or stuffy nose, sore throat, cough, shortness of breath, nausea, vomiting, diarrhea, abnormal taste, and abnormal smell. Taste and smell abnormalities were scored on a 3-point scale (0: as usual, 1: not as much as usual, 2: not at all).

In the Guide to the Clinical Practice of New Coronavirus Infections (Edition 10.0), treatment without antiviral drugs is an option for patients at low risk of severe disease, and we believe that placebo administration in the subjects of this study (patients with mild disease) is feasible. There is still no established prophylaxis or treatment for post-associated symptoms, and there are no existing drugs that can be used as a control. Therefore, we believe that it is ethically acceptable to use placebo as a control.

- 1. Number of planned research subjects for this study

Total of 2,000 cases as the number of study subjects to be enrolled

Encitrelvir group: 1,000 cases

Placebo group: 1,000 cases

[Basis for setting the number of subjects for the planned research: ]

In the Japanese ITT population in the Phase 3 part of the global Phase 2/3 study [T1221 study] of encitrelvir fumarate, the proportion of subjects who had "any of the following symptoms after COVID-19: fatigue, shortness of breath or dyspnea, abnormal sense of smell or taste at both 1 month and 3 months from the start of treatment" or "any of the following symptoms at 3 months: poor concentration and thinking, poor problem solving, and forgetfulness (short-term or long-term)" was 22.4% in the placebo group and 22.4% in the encitrelvir group. The proportion of study subjects who had "any of the following symptoms after COVID-19: fatigue, shortness of breath or dyspnea, abnormal sense of smell, or abnormal taste at both 1 and 3 months of treatment," or "decreased ability to concentrate or think, poor problem solving, or forgetfulness (short-term or long-term) at 3 months" was 22.4% for placebo and 15.4% for the encitrelvir group, for a relative risk of 68.9%.

In the most recent global Phase III study of encitrelvir fumarate [SCORPIO-HR study], which was ongoing at the time of the study design, the combined incidence of post-epidemic symptoms in the actual drug and placebo groups was 12.4% as of October 2023 in a blinded fashion. Considering that the incidence of post-illness symptoms varied by epidemic strain, this incidence rate was used as the overall incidence rate. Assuming an overall incidence of 12.4% and a relative risk of 70%, the incidence of post-embolic symptoms was calculated to be 14.6% in the placebo group and 10.2% in the encitrelvir group.

The null hypothesis is that the placebo and encitrelvir groups have the same incidence of post-illness symptoms, and the alternative hypothesis is that the placebo and encitrelvir groups do not have the same incidence of post-illness symptoms. The number of cases required was calculated by testing for differences in mother proportions.

[If an interim analysis is to be performed]

Under the assumption that the fraction of information at the time of the interim analysis was 50% and that the efficacy discontinuation criterion was based on an alpha consumption function with O'Brien-Flemming bounds, the required number of cases was calculated to be 1,818, assuming an outcome proportion of 14.6% in the non-exposed group, a conservative relative risk of 70%, a level of significance of 5% bilaterally, and a power of 80%. The number of cases required for the study was calculated to be 1,818, with a significance level of 5% bilaterally and a power of 80%. Assuming that approximately 10% of the patients would drop out, the target number of cases was set at 2,000.

[If no interim analysis is performed]

The number of cases required was calculated to be 1,784 when the proportion of outcomes in the non-exposed group was set at 14.6%, the relative risk at 70%, the significance level at 5% bilaterally, and the power at 80%. Assuming that approximately 10% of the patients would drop out, the target number of cases was set at 2,000.

- 1. Planned duration of this study

1. Planned Research Period

jRCT publication date～ December 31, 2026

1. Expected registration period

jRCT publication date～ December 31, 2025

1. Expected Observation Period

jRCT publication date～ June 30, 2026

1. Selection of research subjects

Patients who meet all the selection criteria and do not violate the exclusion criteria will be considered for the study.

- 1. criterion (criteria) for selection

1. Persons diagnosed with COVID-19 by nucleic acid amplification method (PCR, LAMP method, etc.), antigen qualitative test or antigen quantitative test at the medical institution referring the research subject
2. Persons with a mild disease severity classification in the Clinical Practice Guide for New-type Coronavirus Infections (COVID-19) (Edition 10.0) at the time of obtaining consent.
3. Expected to start taking study medication within 72 hours of onset*.
    When the patient is judged to have one or more of the 14 symptoms of *COVID-19 (fatigue, muscle aches or pains, headache, chills/sweats, fever or fevers, runny or stuffy nose, sore throat, cough, shortness of breath [dyspnea], nausea, vomiting, diarrhea, abnormal taste, abnormal sense of smell)
4. Research subject referral Persons with a body temperature of 37.0°C or higher at the time of definitive diagnosis at a medical institution.
5. Women of childbearing potential who are able to use adequate contraception with their partner when having sex during the study drug and within 2 weeks after the last dose
6. Persons who are at least 18 years of age at the time of obtaining consent.
7. Persons whose written consent to participate in this study is obtained from the individual

[Basis for setting selection criteria ]

(1)～ (3) To select appropriate study subjects for evaluation of the efficacy of encitrelvir fumarate.

(4) Because a subgroup analysis of the Phase 3 part of the international Phase 2/3 study [T1221 study] showed a higher incidence of post-illness symptoms in subjects whose body temperature was 37°C or higher.

(5) Because encitrelvir fumarate has been shown to cause fetal teratogenicity in rabbit fetuses in animal studies, and although the effects in humans are not known, taking the drug during pregnancy may cause fetal malformations. The contraceptive period after the last dose is equal to five times the median (51.4 hours) and maximum (66.4 hours) half-life of encitrelvir fumarate in studies in healthy adult women.

(6) (7) To conduct research on adults with appropriate consent.

- 1. exclusion criteria

1. Persons at high risk for severe COVID-19
2. Antiviral drugs indicated for COVID-19 (e.g., remdesivir, mornupiravir, nilmatrelvir/ritonavir, encitrelvir fumarate), neutralizing antibody drugs (cacirivimab/imdevimab, sotrovimab. etc.) since the current COVID-19 or within 15 days before the date consent was obtained, tixagevimab/silgavimab, etc.).
3. Patients who have received anti-IL-6 antibody products (e.g., tocilizumab), JAK inhibitors (e.g., baricitinib, etc.; excluding topical application), oral, suppository, or injectable steroids within 2 weeks prior to obtaining consent. ), oral, suppository or injectable steroids within 2 weeks prior to obtaining consent.
4. Have participated in a clinical trial of a COVID-19 treatment since the onset of this COVID-19
5. Those unable to meet online with Principal Investigator or Research Affiliate using a smart phone
6. Persons unable to enter information into the patient-reported outcomes collection system using a smart phone
7. Patients with a history of hypersensitivity to any ingredient of encitrelvir products
8. Patients who are receiving or are scheduled to receive during the study drug administration period and within 2 weeks after the last administration of the study drug any drug that is contraindicated with encitrelvir fumarate (see section 7.3).
9. Patients with renal or hepatic dysfunction who are receiving colchicine
10. Women who are pregnant or possibly pregnant (the possibility of pregnancy shall be determined after checking menstrual history and recent sexual activity)
11. Lactating women
12. Persons with severe hepatic dysfunction
13. Immunocompromised patients and those on dialysis

Refer to Table 37 in the "Infectious Diseases Society of Japan Vaccine Committee, COVID-19 Vaccine Task Force: Recommendations on COVID-19 Vaccine (7th Edition).

1. Persons with other coexisting infectious diseases
2. Persons who are currently participating in another intervention study or who have participated in this study
3. Have participated in a pharmaceutical clinical trial within the past year
4. Persons deemed inappropriate as research subjects by the principal investigator or subinvestigator for other reasons

[Rationale for setting exclusion criteria: ]

(1) Because the Guide for the Treatment of Novel Coronavirus Infections (COVID-19) (Edition 10.0) recommends nilmatrelvir/ritonavir, lemdecivir or mornupiravir for patients at high risk of severe disease.

(2) - (4) This was established to avoid influence on the efficacy evaluation by the administration of COVID-19 therapeutic agents. Anti-IL-6 antibody preparations, JAK inhibitors (except for topical application), and oral, suppository, or injectable steroids that do not have an indication for COVID-19 may also affect the efficacy evaluation.

(5) To conduct online interviews with research subjects by the principal investigator or a research associate.

(6) To use patient-reported outcome collection systems.

(7)～ (10) Encitrelvir fumarate is contraindicated and the safety of the study subjects was considered.

(11) Because administration of Encitrelvir fumarate is not recommended and the safety of infants was considered. (12) Encitrelvir Because administration of fumaric acid is not recommended and the safety of the study subjects was considered.

(13) Because of the risk of COVID-19 severity due to reduced immunocompetence, and because of safety considerations when placebo was administered.

(14) Because it is difficult to evaluate efficacy when symptoms such as fever are present due to the coexistence of other infectious diseases.

(15) Because of the potential for unanticipated effects on this study and on the intervention studies in which they are participating.

(16) Encitrelvir fumarate is a strong CYP3A inhibitor and also inhibits P-gp, BCRP, OATP1B1, and OATP1B3, and there is a possibility of unknown interactions with the investigational drug. Conservatively, "within the past year" was chosen, assuming that some investigational drugs have a long half-life.

(17) To exclude persons whom the principal investigator or subinvestigator determines to be inappropriate for this study for reasons other than those listed above.

1. Investigational Product
   1. Outline of the pharmaceuticals, etc. that are the subject of the research

The following study drugs (test and control drugs) will be used in this study.

| Name of test drug | Encitrelvir fumarate 125 mg/Placebo |
| --- | --- |
| Serial number or serial number | CF21014 |
| outward appearance | 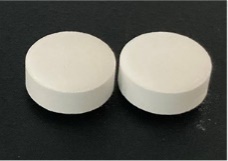  Approximately 9.0 mm in diameter and 4.9 mm thick bare tablets  Test and control drugs are identical in appearance and cannot be distinguished. |
| Storage | Store at room temperature |

- - 1. test drug

| Not approved under the Pharmaceuticals and Medical Devices Law,  Off-label, on-label | | □ unapproved | Off-label | ■ within the scope of approval |
| --- | --- | --- | --- | --- |
| Generic name (if not approved in Japan or abroad, provide the development code) | | Encitrelvir fumarate | | |
| Trade name (for overseas products, also list the name of the country) | | Zocova Tablets 125 mg | | |
| Name of manufacturing and sales company | | Shionogi & Co. | | |
| approval number | | 30400AMX00205000 | | |
| Provider of Test Drugs, etc. | name | Shionogi & Co. | | |
|  | Location | 3-1-8 Doshomachi, Chuo-ku, Osaka | | |

- - 1. control drug

| Not approved under the Pharmaceuticals and Medical Devices Law,  Off-label, on-label | | Not approved | Off-label | Within □ Approval |
| --- | --- | --- | --- | --- |
| Generic name (if not approved in Japan or abroad, provide the development code) | | placebo | | |
| Test Drug Provider (Drugs, etc.) | name | Shionogi & Co. | | |
|  | Location | 3-1-8 Doshomachi, Chuo-ku, Osaka | | |

- 1. Ensuring the quality of pharmaceuticals and other products that are the subject of research

The Principal Investigator shall

1 Documents describing the ingredients, quantities, specifications and research methods, matters concerning performance and structure, matters concerning methods of manufacturing, etc., matters concerning packaging and labeling of pharmaceutical products, etc., methods of use in clinical research, and other necessary matters shall be prepared or obtained and stored.

(2) The date of manufacture, serial number or production code of the drug product, etc., and other records relating to the manufacture of said drug product, etc., shall be recorded and preserved.

3 The quantity and date of acquisition of pharmaceuticals and other supplies shall be recorded and stored.

4 For each research subject, the quantity and date of use of the drug or other substance shall be recorded and stored.

5 With respect to the disposal of pharmaceutical products, etc., the quantity and date of disposal shall be recorded and preserved.

6 When information is obtained that the quality of a drug or other substance used in clinical research is defective, etc., it shall be verified, and a report on the measures to be taken, such as suspension of the clinical research, shall be made to the Accredited Clinical Research Review Committee. In addition, a record of such report shall be made.

7 When it is judged necessary to recall a drug or other product due to reasons such as the quality of the drug or other product used in clinical research being defective, the Committee shall promptly report the matter to the Authorized Clinical Research Review Committee and shall also perform the following duties

(a) Promptly instruct the principal investigator to discontinue use and recall the drug or other product.

(b) Prepare and maintain a record of the recall process that describes the details of the recall, the results of the investigation into the cause, and the remedial measures taken.

The principal investigator shall

1 Upon receipt of notification from the principal investigator, instructions to discontinue use and recall of the drug or other product will be promptly given to the research physicians and others involved in the research.

1. Method of Administration (Protocol Treatment)
   1. Application of the drug or other product under study Methods
      1. Encitrel Building Group

Encytrelvir is administered orally at a dose of 375 mg on day 1 and 125 mg once daily on days 2 through 5.

- - 1. Placebo group

Placebo is administered orally at a dose of 3 tablets on day 1 and 1 tablet once daily on days 2 through 5.

- - 1. Basis for establishing the method of application of the drug or other product under study

Followed the approved dosage and administration of Encitrelvir fumarate.

- 1. Concomitant medications/adjunctive therapy

Symptomatic treatment of COVID-19 symptoms (e.g., antipyretic analgesics and antitussives) and symptomatic treatment of post-affected symptoms is acceptable.

For comorbidities, concomitant medications and adjunctive therapies are administered as part of routine medical care.

- 1. drug use prohibited in combination with other drugs

The use of the following drugs is prohibited during the administration of the study drug and within 2 weeks after the last dose

Drugs contraindicated for co-administration with ensitrelvir fumarate (the following are listed in the May 2025 revision (22nd edition). The latest package insert must be checked). Pimozide, quinidine sulfate hydrate, bepridil hydrochloride hydrate, ticagrelor, eplerenone, ergotamine tartrate/caffeine anhydrous isopropylanthipyrine, ergometrine maleate, methylergometrine maleate, dihydroergotamine mesylate, simvastatin, Triazolam, anamorelin hydrochloride, ibabradine hydrochloride, venetoclax [during dose escalation for relapsed or refractory chronic lymphocytic leukemia (including small lymphocytic lymphoma)], ibrutinib, blonanserin, lurasidone hydrochloride, azelnidipine, azelnidipine olmesartan medoxomil, Suvorexant, tadalafil (Adsilca), vardenafil hydrochloride hydrate, romitapide mesylate, rifabutin, finelenone, reversavan, riociguat, apalutamide, carbamazepine, enzalutamide, mitotane, phenytoin, fosphenytoin sodium hydrate, Rifampicin, St. John's Wort (St. John's Wort) containing foods

1. Antiviral drugs with efficacy against COVID-19 (e.g., remdesivir, mornupiravir, nilmatrelvir/ritonavir), neutralizing antibody drugs (e.g., sotrovimab, cacirivimab/imdevimab, tixagevimab/silgavimab)
2. Anti-IL-6 antibody preparations (e.g., tocilizumab), JAK inhibitors (e.g., baricitinib. Steroids for oral, suppository or injection (excluding topical application)

[Rationale for setting up concomitant use of prohibited drugs: ]

(1) To ensure the safety of research subjects.

(2) (3) Because it may affect the validity assessment in this study.

1. Schedule of Assessments

The principal investigator or research associate will collect data according to the following Observation, Testing, and Evaluation Schedule .

- 1. Observation, examination and evaluation schedule
     1. At registration (Visit 1)
- Background of Study Subjects
- Height and Weight
- co-morbid disease
- pre-existing medical condition
- Online Interview
- Presence of risk factors for severe disease
- Check concomitant medications
- Pregnancy Confirmation
  - 1. Start date of study medication
- quality of life
- labor productivity
  - 1. After 1 week (Visit 2)
- Online Interview
- Drugs taken in the study
- Check concomitant medications
- Pregnancy Confirmation
- adverse event
  - 1. After 1 month (Visit 3), 2 months (Visit 4), 3 months (Visit 5), 4 months (Visit 6), 5 months (Visit 7), 6 months (Visit 8)
- post-affective symptoms
- quality of life
- labor productivity
- COVID-19 Re-incidence of COVID-19
  - 1. At discontinuation of follow-up period
- Post-illness symptoms (whenever possible)
- Quality of life (as much as possible)
- Labor productivity score (where possible)
- COVID-19 Re-incidence (as much as possible)
  1. Study Calendar

|  | pre-contact period | curative phase | | Tracking period | | | | | | | |
| --- | --- | --- | --- | --- | --- | --- | --- | --- | --- | --- | --- |
|  | at registration | Test Drugs  Dose  Start Date | After 1 week^*2^ | | After 1 month | After 2 months | After 3 months | After 4 months | After 5 months | After 6 months | time of discontinuance |
| Visit | 1 | - | 2 | | 3 | 4 | 5 | 6 | 7 | 8 |  |
| Day |  | 0 | 7 | | 28 | 56 | 84 | 112 | 140 | 168 |  |
| tolerance level | -^*1^ | - | -2 to +5 | | ±7 | ±7 | ±7 | ±7 | ±7 | ±7 |  |
| Obtaining Consent | ● |  |  | |  |  |  |  |  |  |  |
| Registration and allocation | ● |  |  | |  |  |  |  |  |  |  |
| Background of Study Subjects | ● |  |  | |  |  |  |  |  |  |  |
| Height and Weight | ● |  |  | |  |  |  |  |  |  |  |
| co-morbid disease | ● |  |  | |  |  |  |  |  |  |  |
| pre-existing medical condition | ● |  |  | |  |  |  |  |  |  |  |
| Start taking study medication |  | ● |  | |  |  |  |  |  |  |  |
| record of test drug doses |  |  |  | |  |  |  |  |  |  |  |
| Online Interview | ● |  | ● | |  |  |  |  |  |  |  |
| Presence of risk factors for severe disease | ● |  |  | |  |  |  |  |  |  |  |
| Check concomitant medications | ● |  | ● | |  |  |  |  |  |  |  |
| Pregnancy Confirmation | ● |  |  | |  |  |  |  |  |  |  |
| post-affective symptoms |  |  |  | | ● | ● | ● | ● | ● | ● | period |
| quality of life |  | ● |  | | ● | ● | ● | ● | ● | ● | period |
| labor productivity |  | ● |  | | ● | ● | ● | ● | ● | ● | period |
| Medical visits and prescriptions for medications |  |  |  | | ● | ● | ● | ● | ● | ● | period |
| COVID-19 Re-incidence of COVID-19 |  |  |  | | ● | ● | ● | ● | ● | ● | period |
| Adverse events ^*3^ |  |  |  | |  |  |  |  |  |  |  |

●: Required items, 0: Items to be implemented whenever possible

*1: The study drug should be addressed so that it can be started within 72 hours of the onset of symptoms.

*2: Whenever possible, an online interview will be conducted when study medication or the study is discontinued.

*3: Collection of adverse events will begin on Day 0 and continue until 2 weeks after the last dose of study drug.

1. Observation, examination and evaluation procedures
   1. Decentralized Clinical Trials support system used in this study

| System Name (Provider) | summary |
| --- | --- |
| MiROHA  (MICIN Corporation) | The system is accessed by a browser and used for online interviews and eConsent by the principal investigator, principal investigator and research subjects.  It will also be used by the principal investigator and research associate physicians to record data. |
| Study Concierge  (Buzzreach Inc.) | Using an application installed on the smart phones of the research subjects, the research subjects will record their medication status, post-illness symptoms, quality of life, and labor productivity. |

- 1. Recruitment of research subjects

When a patient with suspected COVID-19 is seen, the physician at the referring institution for research subjects will make a definitive diagnosis of COVID-19 by nucleic acid amplification (PCR, LAMP, etc.), antigen qualitative test, or antigen quantitative test. Patients with a confirmed diagnosis of COVID-19 will be asked if they are willing to receive an explanation of this study from the principal investigator or subinvestigator, after symptomatic treatment if necessary. Patients who are willing to receive an explanation will be referred to the recruitment website. The patient enters his/her name, cell phone number, e-mail address, mailing address, and desired date and time of the online interview on the recruitment site. The "confirmation e-mail" sent after entering the information on the recruitment site will include a link to the consent document so that the patient can read the consent document before the online interview.

The Principal Investigator, Research Associate or Research Collaborator will confirm the desired date and time of the online interview on the recruitment website, determine the time of the online interview, and then communicate this information to the patient.

- 1. Research Candidate Registration

The principal investigator, research associate or research collaborator will register the patient's name and e-mail address on the MiROHA eConsent screen.

- 1. Obtaining Consent

The principal investigator or a research associate will meet with the patient online to explain the study and obtain consent from the patient (see Section 15.4.1 for detailed procedures for obtaining consent).

- 1. Registration, allocation and sending of study drugs
     1. Registration, allocation and study drug delivery procedures

1. The principal investigator or research associate evaluates the eligibility of research subjects for whom consent has been obtained based on the selection and exclusion criteria, and enters the necessary information into the Web-based allocation system for research subjects who are determined to be eligible.
2. The study drug to be administered to the subject is determined in the Web allocation system.
3. The study drug distribution center will send the allocated study drug to the location designated by the research subject.
4. The study subject will record receipt of the study drug in the study concierge.
   - 1. Creation and storage of allocation procedures

The allocation manager will develop allocation procedures and maintain allocation information for research subjects.

Allocation information will be stored in a secure location and will not be available to all parties, including the study subjects, principal investigators, principal investigators and subinvestigators, to maintain blinded access.

Key openings for the entire study will be conducted after the data are fixed for final analysis. Interim analyses will be conducted by the Independent Data Monitoring Committee. At that time, allocation information will be submitted only to the Independent Data Monitoring Committee and will not be available to all parties involved, including the study subjects, principal investigators and subinvestigators.

- - 1. emergency key opening

1. Emergency key openings are permitted only in emergency situations, such as when a serious adverse event occurs in a research subject and it becomes necessary to specify the treatment to be given to the research subject in order to provide appropriate medical treatment.
2. When emergency key opening becomes necessary, perform emergency key opening according to the allocation procedure.
   1. record of test drug doses

The study subjects will record in the Study Concierge whether or not they are taking the study medication on each dose day and the time of the dose (first dose day only).

- 1. Background of Study Subjects

The following survey and evaluation items will be identified

- Date of Agreement
- Date of birth
- gender
- Ethnicity, race
- COVID-19 Date of onset
- COVID-19 confirmed diagnosis (date of diagnosis, test method)
- Body temperature at the time of definitive diagnosis at the referring medical institution of the research subject
- Degree of COVID-19 symptoms (fatigue, muscle aches or pains, headache, chills/sweats, fever or fevers, runny or stuffy nose, sore throat, cough, shortness of breath, nausea, vomiting, diarrhea, abnormal taste, and abnormal smell) at the time of consent
- Vaccination history (if vaccination exists, number of vaccinations and date of last vaccination)
- Smoking status
  1. Height and Weight

Obtain height and weight from the research subject.

Height in cm (integer)

Weight in kg (integer)

BMI is calculated during statistical analysis based on height and weight.

- 1. co-morbid disease

Identify another comorbid condition concurrent with COVID-19 at the time consent is obtained.

- 1. pre-existing medical condition

Identify illnesses requiring hospitalization within 1 year prior to obtaining consent and illnesses requiring a hospital visit within 12 weeks prior to obtaining consent.

To confirm whether or not and when COVID-19 is present.

- 1. Presence of risk factors for severe disease

Evaluate the presence of risk factors for severe disease based on information such as age, gender, BMI, and comorbidities.

- 1. Check concomitant medications

Confirm concomitant medications (drug name, duration of use, and reason for use) with the study subjects.

- 1. Pregnancy

In women, the presence or absence of pregnancy is confirmed by a medical interview.

- 1. post-affective symptoms

Study subjects enter the severity of post-illness symptoms and other information (Appendix 1) into the Study Concierge.

- 1. quality of life

Study subjects will enter their responses to the EQ-5D-5L (Appendix 2) into Study Concierge.

- 1. labor productivity

Study subjects will enter their responses to the Work Productivity and Activity Impairment Questionnaire: General Health Japanese version (Appendix 3) into the Study Concierge.

- 1. Medical visits and prescriptions for medications

Study subjects enter answers to questions about their medical visits and medication prescriptions (Appendix 4) into the Study Concierge.

- 1. COVID-19 Re-incidence of COVID-19

Study subjects will enter their answers to the COVID-19 recurrence questions (Appendix 5) into the Study Concierge.

- 1. Criteria for Discontinuation and Termination by Study Subject
     1. Discontinuation per research subject

If any of the following are found to be the case after enrollment of a study subject, the principal investigator or subinvestigator will discontinue study medication for that subject. The discontinued subjects will be evaluated at the time of discontinuation and moved to the follow-up period as prescribed.

1. If an adverse event occurs and the principal investigator or a subinvestigator determines that the continuation of the study poses an unacceptable risk to the health of the research subjects
2. If the research subject requests to stop taking the study medication
3. If it becomes necessary to administer a drug that is contraindicated with encitrelvir fumarate
4. Other COVID-19 medications or respiratory therapy are required, or if comorbidities necessitate the administration of COVID-19 medications that are also indicated for the disease in question.
5. If you are found to be inappropriate as a research subject
6. If a female research subject is found to be pregnant
7. Other cases in which the principal investigator or a subinvestigator determines that the intervention should be discontinued

If, after enrollment of a research subject, it is found that any of the following applies to the subject, the principal investigator or a subinvestigator will discontinue the research on the subject.

1. If the research subject requests discontinuation of the research
2. If it proves impossible to perform the required observation in the future due to the convenience of the research subject, such as a malfunction of the smartphone
3. If a study subject is found to have COVID-19 again during the follow-up period
4. If you are found to be unsuitable as a research subject prior to taking the study medication
5. Other cases in which the principal investigator or subinvestigator determines that the research should be terminated
   - 1. Discontinuation procedures for each research subject

If it is found that the discontinuation criteria are met, the principal investigator or subinvestigator will immediately explain this to the research subjects and take alternative treatment or other measures as necessary.

In the event of discontinuation during the treatment phase, the principal investigator or research associate will conduct the observations and assessments planned for this study during the Visit 2 online interview whenever possible and record the results along with the reason for discontinuation in the case report form at .

In the event of discontinuation during the follow-up period, the study subject will conduct the evaluation planned for this study whenever possible and enter it into the Study Concierge along with the reason for discontinuation.

- - 1. Termination per research subject

The completion of all planned observations, examinations, and evaluations described in this research protocol shall constitute the termination of the subject of the study.

1. adverse event
   1. Definition of adverse events

An adverse event is any unwanted or unintended sign (including abnormal laboratory values), symptom, or illness that occurs in a study subject, whether or not causally related to the study. Any worsening of symptoms observed prior to the start of the study that occur after administration will also be treated as an adverse event.

- 1. Collection period for adverse events

The collection of adverse events will begin at the start of the study drug dose and will continue until 2 weeks after the last dose of study drug.

- 1. Confirmation of subjective symptoms

The principal investigator or research associate will confirm from the research subject the presence or absence of subjective symptoms during the online interview.

- 1. Adverse Event Assessment

The principal investigator or research associate will record the following on the case report form for each adverse event observed during the collection period. If more than one adverse event is observed, record each event separately.

1. Adverse event name
2. expression date
3. severity (of an illness)
4. Severity (non-serious, serious)
5. Causal Relationship to Research
6. Causal relationship with study drug
7. day on which judgment and haste are avoided (judgement)
8. return to origin
   - 1. Adverse event name

List each event by its diagnostic name. Signs (including abnormal laboratory values and abnormal ECG findings) and symptoms associated with that diagnostic name should not be listed as a separate adverse event. If the diagnosis is unknown, list the signs or symptoms as an adverse event, as appropriate.

- - 1. expression date

The following criteria are used to determine the date of onset of an adverse event.

| adverse event | expression date |
| --- | --- |
| For signs, symptoms, and diseases (diagnostic name) | State the date when the research subject or the principal investigator or research associate first became aware of the signs and symptoms of the adverse event. |
| For asymptomatic disease | State the date the test was performed for diagnosis and the date the diagnosis was confirmed.  State the date when the diagnosis was confirmed, even if the laboratory findings show evidence of an old condition or the time of occurrence can be approximately estimated. |
| In case of exacerbation of comorbidities | State the date when the research subject or the principal investigator or subinvestigator first became aware of the disease or worsening of symptoms. |
| In the event of an abnormality in an examination after the start of application of the drug, etc. subject to this study | List the dates of any laboratory tests with abnormal laboratory values that are considered clinically problematic. |
| If an abnormality is found in the examination at the time of the start of application of the drug, etc. subject to this study and worsens in subsequent examinations | List the date of the test when there was a clear increase, decrease, increase, or decrease as determined by medical judgment based on the laboratory value trends. |

- - 1. severity (of an illness)

Classify the severity of adverse events as follows

| light degree | Transient and easily tolerated |
| --- | --- |
| moderate | To the extent that it interferes with normal activities |
| High degree | To the extent that it makes normal activities impossible |

- - 1. seriousness

Regardless of the severity of the adverse event, the following are considered serious

1. death
2. Risk of death
3. Admission to a medical institution for treatment or prolongation of hospital stay
4. disability
5. risk of injury
6. Severe according to the above
7. Congenital diseases or anomalies in later generations
   - 1. Causal Relationship to Research

The causal relationship between the conduct of this study and the adverse event shall be classified as follows. If the judgment is made that there is no causal relationship, the reason for the judgment should be recorded in the case report form.

| Relevant. | Adverse events that can be reasonably attributed to the conduct of this study |
| --- | --- |
| No relation | Adverse events that cannot be reasonably attributed to the conduct of this study |

See Section 10.4.6 for examples of what can be reasonably explained.

- - 1. Causal relationship with study drug

Classify the causal relationship between the study drug and the adverse event as follows. If it is judged that there is no causal relationship, the reason for the judgment should be recorded in the case report form.

| Relevant. | Adverse events that can be reasonably explained as being caused by the study drug |
| --- | --- |
| No relation | Adverse events that cannot be reasonably attributed to the study drug |

An adverse event can be reasonably explained as being caused by the study drug if, for example, similar adverse events have been reported and can be explained by the pharmacological effects of the study drug, if an increase or decrease in dosage affects the occurrence or severity of the adverse event, or if information on medical history/complications and concomitant medications is collected to the extent possible and considered, but cannot be explained by factors other than the study drug. The case in which the adverse event cannot be explained by factors other than the study drug, even after collecting as much information as possible on history/comorbidities and concomitant medications.

- - 1. day on which judgment and haste are avoided (judgement)

The date of resolution or recovery of symptoms or the date of determination of the outcome of the adverse event. If the outcome is "death," the date of death shall be the date of death.

- - 1. return to origin

Classify adverse event outcomes as follows

| classification | evaluation criteria |
| --- | --- |
| recovery | Resolution or recovery of symptoms  Normalization of laboratory values or restoration to pre-dose laboratory values |
| light | Symptoms almost disappeared  Laboratory values have improved but have not normalized or recovered to pre-dose laboratory values  Deaths in which the adverse event was not the direct cause of death, and the deaths occurred while the adverse event was still minor. |
| unrecovered | No change in symptoms or laboratory values  Symptoms, findings, and laboratory values on the last day of the observation period are worse than the severity at the time of onset.  Irreversible congenital anomalies  Deaths in which the adverse event was not the direct cause of death, and in which the adverse event remained unrecovered. |
| Recovered but with sequelae | Remained dysfunctional to the extent that it interfered with daily life. |
| death | Direct association was found between death and the adverse event.  Directly related" means that the adverse event caused or clearly contributed to the death.  Death is not considered as an outcome for adverse events that are judged (judged or presumed) not to be the direct cause of death in the same case. |
| unknown | The progress after the date of onset of symptoms cannot be traced in this study protocol due to transfer to a different hospital, change of residence, etc. |

- 1. Measures to be taken in case of adverse events
     1. Treatment of research subjects

In the event of an adverse event, the principal investigator and the research physician will recommend that the research subject receive medical care at the medical institution to which the research subject was referred. If necessary, the investigators will seek to resolve the problem and determine the cause by having the subject undergo diagnosis and treatment by a specialist physician.

- - 1. Adverse event follow-up

Adverse events that occur during the study period will be followed by telephone whenever possible until they recover or are determined to be no longer clinically necessary.

- - 1. Reporting to funding companies

In the event of an adverse event, the principal investigator or a subinvestigator will report it in accordance with the procedures specified by the fund provider, Shionogi & Co. The report should not contain information that could identify the research subjects.

- 1. Adverse events expected in this study

The adverse reactions listed in the Encytrelvir Fumarate package insert (July 2024 revision (14th edition)) are as follows The principal investigator or research associate should review the most current version of the package insert when administering the study drug.

(1) Serious side effects

Shock, anaphylaxis (frequency unknown)

(2) Other side effects

| Type³ Frequency | 5% or more | Less than 1-5 | Less than 1 | frequency unknown |
| --- | --- | --- | --- | --- |
| hypersensitivity |  |  | rash | pruritic |
| digestive organs |  |  | Nausea, vomiting, diarrhea, abdominal discomfort |  |
| psychoneurotic system |  |  | headache |  |
| metabolism |  |  | dyslipidemia (dyslipidemia, dyslipidemia) |  |
| Other | Lower HDL cholesterol (16.6%) | Elevated triglycerides, elevated bilirubin, lowered blood cholesterol | Serum iron elevation |  |

1. Reporting of Diseases and Other Safety Events.
   1. Definition of Disease, etc.

Illness, etc." includes any illness, disability or death or infectious disease suspected to have been caused by the conduct of this research, as well as abnormal laboratory values and various symptoms.

- 1. Procedures for Reporting Serious Diseases, etc. to an Accredited Clinical Research Review Committee

In the event of any of the reportable diseases, etc. listed in 1. through 3. below, the physician in charge will promptly inform the principal investigator. When the principal investigator becomes aware of any of the following matters, he/she shall report it to the administrator of the implementing medical institution within the time period specified for each, and then notify the principal investigator, who shall report it to the Accredited Clinical Research Review Committee and provide information to the other principal investigators. The other principal investigators who receive the information will promptly report the contents of the information to the administrator of the implementing medical institution.

1. the following, which are suspected to be due to the conduct of this study and cannot be predicted: 7 days

A. Death Death

b. Diseases that may lead to death, etc.

2. the following: 15 days

(a) The following The following, which are suspected to be due to the conduct of this research (excluding those listed in 1)

(1) Death

(2) Diseases, etc. that may lead to death

(b) The following The following, which are suspected to be due to the conduct of this research and cannot be predicted

(1) Diseases requiring hospitalization or prolonged hospitalization at a medical institution for treatment

(2) Disability

(3) Diseases, etc. that may lead to disability

(4) Diseases, etc. that are as serious as (1) through (3) above and death or diseases that may lead to death

(5) Congenital diseases or anomalies in subsequent generations

3. suspected to be due to the implementation of this research (other than 1 and 2 above): When making periodic reports to the Authorized Clinical Research Review Committee pursuant to the provisions of Article 17, paragraph (1) of the Act

●Diseases, etc. subject to reporting to an accredited clinical research review committee

| Diseases, etc. | Unpredictable | Predictable |
| --- | --- | --- |
| a. Death | seven days | fifteen days |
| b. Diseases that may lead to death, etc. | seven days | fifteen days |
| c. Diseases that require hospitalization or extended stay at a medical institution for treatment | fifteen days | periodic report |
| d. Obstacles | fifteen days | periodic report |
| e. Diseases, etc. that may lead to disability | fifteen days | periodic report |
| f. Diseases which are as serious as c. through e. and death and diseases which may lead to death. | fifteen days | periodic report |
| g. Congenital diseases or anomalies in subsequent generations | fifteen days | periodic report |
| h. Other diseases, etc. | periodic report | periodic report |

The principal investigator or subinvestigator shall notify the administrator of the institution and the principal investigator of any changes in the reported details of the disease, such as changes in outcome, and the principal investigator shall report to the Accredited Clinical Research Review Committee.

- 1. Procedures for reporting serious illnesses, etc. to the Minister of Health, Labour and Welfare

When the Principal Investigator becomes aware of any of the matters described in 1 and 2(b) of Section 11.2, he/she shall report it to the Minister of Health, Labour and Welfare from the Disease Reporting screen of the jRCT within the time period specified in the table in the same section.

1. data management
   1. Data Management Plan

The data management representative will inquire with the principal investigator or subinvestigator regarding missing values, incomplete values, or suspected erroneous entries in the case report form. The Data Management Officer will confirm the response or data correction by the principal investigator or subinvestigator.

- 1. case report

The principal investigator or subinvestigator will prepare case report forms for all research subjects for whom consent has been obtained.

When preparing case reports, input the information into MiROHA and the Web allocation system.

Changes or amendments to a case report are recorded as an audit trail that documents the information before and after the change or amendment, the person who made the change or amendment, the date of the change or amendment, and the reason for the change or amendment.

The principal investigator is solely responsible for the accuracy and reliability of all data entered into the case report form.

All data entered into the case report form and Study Concierge shall be considered source documents.

1. Statistical Considerations

The detailed data handling and analysis will be separately specified in the "Statistical Analysis Plan". The statistical analysis plan will be fixed by the time of the interim analysis.

- 1. Definition of analysis population

In this study, two types of analysis populations will be established: the "largest analysis population (Full Analysis Set: FAS)" and the "safety analysis population (Safety Analysis Set: SAS)".

FAS is defined as study subjects who have taken the study drug at least once and have been observed, examined and evaluated at one or more time points .

SAS is defined as Study subjects who have taken the study drug at least once

- 1. Data Handling

Data will be handled as follows. The statistical analyst will consult with the principal investigator and data management staff to determine the analytical handling of data on all research subjects prior to data fixation.

- - 1. Handling of measured values outside the specified tolerance range

For data that deviate from the acceptable range of measurement for the evaluation item, the statistical analysis supervisor will decide whether to accept or reject the data after consultation with the principal investigator and the data management staff. If there are multiple data within the acceptable range, the absolute value of the difference in the number of days from the specified evaluation date shall be calculated, and the data with the smallest absolute value shall be adopted as the data for that evaluation period. If the absolute values are the same, the data shall be examined individually (by evaluation item).

- - 1. Handling of missing values

Missing data will not be supplemented.

- 1. Analysis Method
     1. Background of Study Subjects

Analysis items　　 : Background of the study subjects

Analysis population: FAS, SAS

Analysis Methods　　 : Demographic and other characteristics are summarized using descriptive statistics.

For continuous variables, the number of study subjects, mean, standard deviation, minimum, median and maximum values are calculated. For categorical variables, the number of study subjects, frequency and percentage are calculated.

- - 1. Status of study medication

Analysis Item　　 : Status of study medication

Analysis population: FAS, SAS

Analysis method　　 : Summarize the duration of taking the study drug, whether or not the drug was discontinued, and the reason for discontinuation.

- - 1. concomitant medications

Analysis item　　 : Concomitant medications

Analysis population: FAS, SAS

Analysis Methods　　 : Summarize with respect to concomitant medications.

- - 1. Efficacy Primary Endpoint

Analysis Item: Percentage of subjects with symptoms of fatigue, shortness of breath or dyspnea, abnormal sense of smell, or abnormal taste at both 1 and 3 months after the start of treatment, or with symptoms of poor concentration and thinking, poor problem solving, or memory loss (short-term or long-term) at 3 months after the start of treatment. Percentage

Analysis population: FAS

Analysis method: The presence or absence of either "symptoms of fatigue, shortness of breath or dyspnea, abnormal sense of smell, or abnormal taste at both 1 month and 3 months after the start of treatment" or "symptoms of poor concentration and thinking, poor problem solving, or forgetfulness (short-term or long-term) at 3 months" was used as the response variable. A modified Poisson regression model will be applied using presence/absence as the response variable and the treatment group, COVID-19 vaccination history, severity of COVID-19 symptoms (14 symptoms) at the time of consent (<9 points, ≥9 points), age (continuous value), gender, and BMI (<25, ≥25) as explanatory variables. Adjusted risk ratios will be calculated and compared to the placebo group.

- - 1. Efficacy secondary endpoints

Analysis Item: The proportion of subjects who have at least one of the following symptoms: malaise (fatigue), shortness of breath or dyspnea, abnormal sense of smell, or abnormal sense of taste at both 1 month and 3 months after the start of treatment, OR who have at least one of the following symptoms at 3 months: decreased concentration/thinking ability, decreased problem-solving ability, or memory loss (short- or long-term), (with the symptoms being considered related or of unknown relation to COVID-19). Analysis Population: FAS Analysis Method: A modified Poisson regression model will be applied, using the presence or absence of either of the following as the response variable: "at least one of the symptoms of malaise (fatigue), shortness of breath or dyspnea, abnormal sense of smell, or abnormal sense of taste, judged by the subjects themselves as being related or of unknown relation to COVID-19, at both 1 and 3 months after the start of treatment" OR "at least one of the symptoms of decreased concentration/thinking ability, decreased problem-solving ability, or memory loss (short- or long-term), judged by the subjects themselves as being related or of unknown relation to COVID-19, at 3 months after the start of treatment". The explanatory variables will be treatment group, presence or absence of COVID-19 vaccination history, severity of COVID-19 symptoms (14 symptoms) at the time of consent (less than 9 points, 9 points or more), age (continuous variable), sex, and BMI (less than 25, 25 or more). The adjusted risk ratio will be calculated and compared with the placebo group.

Analysis Item: The proportion of subjects who have at least one of the following symptoms: malaise (fatigue), shortness of breath or dyspnea, abnormal sense of smell, or abnormal sense of taste at all three time points (1, 3, and 6 months) after the start of treatment, OR who have at least one of the following symptoms at 6 months: decreased concentration/thinking ability, decreased problem-solving ability, or memory loss (short- or long-term). Analysis Population: FAS Analysis Method: A modified Poisson regression model will be applied, using the presence or absence of either of the following as the response variable: "at least one of the symptoms of malaise (fatigue), shortness of breath or dyspnea, abnormal sense of smell, or abnormal sense of taste at all three time points (1, 3, and 6 months) after the start of treatment" OR "at least one of the symptoms of decreased concentration/thinking ability, decreased problem-solving ability, or memory loss (short- or long-term) at 6 months after the start of treatment". The explanatory variables will be treatment group, presence or absence of COVID-19 vaccination history, severity of COVID-19 symptoms (14 symptoms) at the time of consent (less than 9 points, 9 points or more), age (continuous variable), sex, and BMI (less than 25, 25 or more). The adjusted risk ratio will be calculated and compared with the placebo group.

Analysis Item: The proportion of subjects who have at least one of the following symptoms: malaise (fatigue), shortness of breath or dyspnea, abnormal sense of smell, or abnormal sense of taste at all three time points (1, 3, and 6 months) after the start of treatment, OR who have at least one of the following symptoms at 6 months: decreased concentration/thinking ability, decreased problem-solving ability, or memory loss (short- or long-term), (with the symptoms being considered related or of unknown relation to COVID-19). Analysis Population: FAS Analysis Method: A modified Poisson regression model will be applied, using the presence or absence of either of the following as the response variable: "at least one of the symptoms of malaise (fatigue), shortness of breath or dyspnea, abnormal sense of smell, or abnormal sense of taste, judged by the subjects themselves as being related or of unknown relation to COVID-19, at all three time points (1, 3, and 6 months) after the start of treatment" OR "at least one of the symptoms of decreased concentration/thinking ability, decreased problem-solving ability, or memory loss (short- or long-term), judged by the subjects themselves as being related or of unknown relation to COVID-19, at 6 months after the start of treatment". The explanatory variables will be treatment group, presence or absence of COVID-19 vaccination history, severity of COVID-19 symptoms (14 symptoms) at the time of consent (less than 9 points, 9 points or more), age (continuous variable), sex, and BMI (less than 25, 25 or more). The adjusted risk ratio will be calculated and compared with the placebo group.

Analysis Item: The proportion of subjects who have at least one of the following symptoms: malaise (fatigue), shortness of breath or dyspnea, abnormal sense of smell, or abnormal sense of taste at both 1 month and 3 months after the start of treatment. Analysis Population: FAS Analysis Method: A modified Poisson regression model will be applied, using the presence or absence of at least one of the symptoms of malaise (fatigue), shortness of breath or dyspnea, abnormal sense of smell, or abnormal sense of taste at both 1 month and 3 months after the start of treatment as the response variable. The explanatory variables will be treatment group, presence or absence of COVID-19 vaccination history, severity of COVID-19 symptoms (14 symptoms) at the time of consent (less than 9 points, 9 points or more), age (continuous variable), sex, and BMI (less than 25, 25 or more). The adjusted risk ratio will be calculated and compared with the placebo group.

Analysis Item: The proportion of subjects who have at least one of the following symptoms: malaise (fatigue), shortness of breath or dyspnea, abnormal sense of smell, or abnormal sense of taste at both 1 month and 3 months after the start of treatment, (with the symptoms being considered related or of unknown relation to COVID-19). Analysis Population: FAS Analysis Method: A modified Poisson regression model will be applied, using the presence or absence of at least one of the symptoms of malaise (fatigue), shortness of breath or dyspnea, abnormal sense of smell, or abnormal sense of taste, judged by the subjects themselves as being related or of unknown relation to COVID-19, at both 1 month and 3 months after the start of treatment as the response variable. The explanatory variables will be treatment group, presence or absence of COVID-19 vaccination history, severity of COVID-19 symptoms (14 symptoms) at the time of consent (less than 9 points, 9 points or more), age (continuous variable), sex, and BMI (less than 25, 25 or more). The adjusted risk ratio will be calculated and compared with the placebo group.

Analysis Item: The proportion of subjects who have each of the following symptoms: malaise (fatigue), shortness of breath or dyspnea, abnormal sense of smell, and abnormal sense of taste at both 1 month and 3 months after the start of treatment. Analysis Population: FAS Analysis Method: A modified Poisson regression model will be applied, using the presence or absence of each individual symptom of malaise (fatigue), shortness of breath or dyspnea, abnormal sense of smell, and abnormal sense of taste at both 1 month and 3 months after the start of treatment as the response variables. The explanatory variables will be treatment group, presence or absence of COVID-19 vaccination history, severity of COVID-19 symptoms (14 symptoms) at the time of consent (less than 9 points, 9 points or more), age (continuous variable), sex, and BMI (less than 25, 25 or more). The adjusted risk ratio will be calculated and compared with the placebo group.

Analysis Item: The proportion of subjects who have each of the following symptoms: malaise (fatigue), shortness of breath or dyspnea, abnormal sense of smell, and abnormal sense of taste at both 1 month and 3 months after the start of treatment, (with the symptoms being considered related or of unknown relation to COVID-19). Analysis Population: FAS Analysis Method: A modified Poisson regression model will be applied, using the presence or absence of each individual symptom of malaise (fatigue), shortness of breath or dyspnea, abnormal sense of smell, and abnormal sense of taste, judged by the subjects themselves as being related or of unknown relation to COVID-19, at both 1 month and 3 months after the start of treatment as the response variables. The explanatory variables will be treatment group, presence or absence of COVID-19 vaccination history, severity of COVID-19 symptoms (14 symptoms) at the time of consent (less than 9 points, 9 points or more), age (continuous variable), sex, and BMI (less than 25, 25 or more). The adjusted risk ratio will be calculated and compared with the placebo group.

Analysis Item: The proportion of subjects who have at least one of the following symptoms at 3 months after the start of treatment: decreased concentration/thinking ability, decreased problem-solving ability, or memory loss (short- or long-term). Analysis Population: FAS Analysis Method: A modified Poisson regression model will be applied, using the presence or absence of at least one of the symptoms of decreased concentration/thinking ability, decreased problem-solving ability, or memory loss (short- or long-term) at 3 months after the start of treatment as the response variable. The explanatory variables will be treatment group, presence or absence of COVID-19 vaccination history, severity of COVID-19 symptoms (14 symptoms) at the time of consent (less than 9 points, 9 points or more), age (continuous variable), sex, and BMI (less than 25, 25 or more). The adjusted risk ratio will be calculated and compared with the placebo group.

Analysis Item: The proportion of subjects who have at least one of the following symptoms at 3 months after the start of treatment: decreased concentration/thinking ability, decreased problem-solving ability, or memory loss (short- or long-term), (with the symptoms being considered related or of unknown relation to COVID-19). Analysis Population: FAS Analysis Method: A modified Poisson regression model will be applied, using the presence or absence of at least one of the symptoms of decreased concentration/thinking ability, decreased problem-solving ability, or memory loss (short- or long-term), judged by the subjects themselves as being related or of unknown relation to COVID-19, at 3 months after the start of treatment as the response variable. The explanatory variables will be treatment group, presence or absence of COVID-19 vaccination history, severity of COVID-19 symptoms (14 symptoms) at the time of consent (less than 9 points, 9 points or more), age (continuous variable), sex, and BMI (less than 25, 25 or more). The adjusted risk ratio will be calculated and compared with the placebo group.

Analysis Item: The proportion of subjects who have each of the following symptoms at 3 months after the start of treatment: decreased concentration/thinking ability, decreased problem-solving ability, and memory loss (short- or long-term). Analysis Population: FAS Analysis Method: A modified Poisson regression model will be applied, using the presence or absence of each individual symptom of decreased concentration/thinking ability, decreased problem-solving ability, and memory loss (short- or long-term) at 3 months after the start of treatment as the response variables. The explanatory variables will be treatment group, presence or absence of COVID-19 vaccination history, severity of COVID-19 symptoms (14 symptoms) at the time of consent (less than 9 points, 9 points or more), age (continuous variable), sex, and BMI (less than 25, 25 or more). The adjusted risk ratio will be calculated and compared with the placebo group.

Analysis Item: The proportion of subjects who have each of the following symptoms at 3 months after the start of treatment: decreased concentration/thinking ability, decreased problem-solving ability, and memory loss (short- or long-term), (with the symptoms being considered related or of unknown relation to COVID-19). Analysis Population: FAS Analysis Method: A modified Poisson regression model will be applied, using the presence or absence of each individual symptom of decreased concentration/thinking ability, decreased problem-solving ability, and memory loss (short- or long-term), judged by the subjects themselves as being related or of unknown relation to COVID-19, at 3 months after the start of treatment as the response variables. The explanatory variables will be treatment group, presence or absence of COVID-19 vaccination history, severity of COVID-19 symptoms (14 symptoms) at the time of consent (less than 9 points, 9 points or more), age (continuous variable), sex, and BMI (less than 25, 25 or more). The adjusted risk ratio will be calculated and compared with the placebo group.

Analysis Item: The proportion of subjects who have not returned to their usual state of health as before COVID-19 at 3 months after the start of treatment and have at least one of the 14 COVID-19 symptoms (malaise (fatigue), body aches or muscle pain, headache, chills, feverishness, runny or stuffy nose, sore throat, cough, shortness of breath or dyspnea, nausea, vomiting, diarrhea, abnormal sense of smell, or abnormal sense of taste). Analysis Population: FAS Analysis Method: A modified Poisson regression model will be applied, using the presence or absence of at least one of the 14 COVID-19 symptoms in subjects who have not returned to their usual state of health as before COVID-19 at 3 months after the start of treatment as the response variable. The explanatory variables will be treatment group, presence or absence of COVID-19 vaccination history, severity of COVID-19 symptoms (14 symptoms) at the time of consent (less than 9 points, 9 points or more), age (continuous variable), sex, and BMI (less than 25, 25 or more). The adjusted risk ratio will be calculated and compared with the placebo group.

Analysis Item: The proportion of subjects who have not returned to their usual state of health as before COVID-19 at 3 months after the start of treatment and have at least one of the 4 neurological symptoms (decreased concentration/thinking ability, decreased problem-solving ability, memory loss (short- or long-term), or insomnia). Analysis Population: FAS Analysis Method: A modified Poisson regression model will be applied, using the presence or absence of at least one of the 4 neurological symptoms in subjects who have not returned to their usual state of health as before COVID-19 at 3 months after the start of treatment as the response variable. The explanatory variables will be treatment group, presence or absence of COVID-19 vaccination history, severity of COVID-19 symptoms (14 symptoms) at the time of consent (less than 9 points, 9 points or more), age (continuous variable), sex, and BMI (less than 25, 25 or more). The adjusted risk ratio will be calculated and compared with the placebo group.

Analysis Item: The proportion of subjects who have not returned to their usual state of health as before COVID-19 at 3 months after the start of treatment and have at least one of the following symptoms: malaise (fatigue), body aches or muscle pain, headache, chills, feverishness, runny or stuffy nose, sore throat, cough, shortness of breath or dyspnea, nausea, vomiting, diarrhea, abnormal sense of smell, abnormal sense of taste, muscle weakness, decreased concentration/thinking ability, decreased problem-solving ability, memory loss (short- or long-term), insomnia, hair loss, palpitations or increased heart rate, joint pain, loss of appetite, dizziness or abnormal balance, chest pain, or skin rash. Analysis Population: FAS Analysis Method: A modified Poisson regression model will be applied, using the presence or absence of at least one of the specified symptoms in subjects who have not returned to their usual state of health as before COVID-19 at 3 months after the start of treatment as the response variable. The explanatory variables will be treatment group, presence or absence of COVID-19 vaccination history, severity of COVID-19 symptoms (14 symptoms) at the time of consent (less than 9 points, 9 points or more), age (continuous variable), sex, and BMI (less than 25, 25 or more). The adjusted risk ratio will be calculated and compared with the placebo group.

- - 1. Efficacy Exploratory Endpoints

Analysis Item: The proportion of subjects who have at least one of the following symptoms: malaise (fatigue), shortness of breath or dyspnea, abnormal sense of smell, or abnormal sense of taste for 2 consecutive time points (2 and 3 months) after the start of treatment, OR who have at least one of the following symptoms at 3 months: decreased concentration/thinking ability, decreased problem-solving ability, or memory loss (short- or long-term). Analysis Population: FAS Analysis Method: A modified Poisson regression model will be applied, using the presence or absence of either of the following as the response variable: "at least one of the symptoms of malaise (fatigue), shortness of breath or dyspnea, abnormal sense of smell, or abnormal sense of taste for 2 consecutive time points (2 and 3 months) after the start of treatment" OR "at least one of the symptoms of decreased concentration/thinking ability, decreased problem-solving ability, or memory loss (short- or long-term) at 3 months". The explanatory variables will be treatment group, presence or absence of COVID-19 vaccination history, severity of COVID-19 symptoms (14 symptoms) at the time of consent (less than 9 points, 9 points or more), age (continuous variable), sex, and BMI (less than 25, 25 or more). The adjusted risk ratio will be calculated and compared with the placebo group.

Analysis Item: The proportion of subjects who have at least one of the following symptoms: malaise (fatigue), shortness of breath or dyspnea, abnormal sense of smell, or abnormal sense of taste for 2 consecutive time points (2 and 3 months) after the start of treatment, OR who have at least one of the following symptoms at 3 months: decreased concentration/thinking ability, decreased problem-solving ability, or memory loss (short- or long-term), (with the symptoms being considered related or of unknown relation to COVID-19). Analysis Population: FAS Analysis Method: A modified Poisson regression model will be applied, using the presence or absence of either of the following as the response variable: "at least one of the symptoms of malaise (fatigue), shortness of breath or dyspnea, abnormal sense of smell, or abnormal sense of taste, judged by the subjects themselves as being related or of unknown relation to COVID-19, for 2 consecutive time points (2 and 3 months) after the start of treatment" OR "at least one of the symptoms of decreased concentration/thinking ability, decreased problem-solving ability, or memory loss (short- or long-term), judged by the subjects themselves as being related or of unknown relation to COVID-19, at 3 months". The explanatory variables will be treatment group, presence or absence of COVID-19 vaccination history, severity of COVID-19 symptoms (14 symptoms) at the time of consent (less than 9 points, 9 points or more), age (continuous variable), sex, and BMI (less than 25, 25 or more). The adjusted risk ratio will be calculated and compared with the placebo group.

Analysis Item: The proportion of subjects who have at least one of the following symptoms: malaise (fatigue), shortness of breath or dyspnea, abnormal sense of smell, or abnormal sense of taste for 2 consecutive time points (2 and 3 months) after the start of treatment. Analysis Population: FAS Analysis Method: A modified Poisson regression model will be applied, using the presence or absence of at least one of the symptoms of malaise (fatigue), shortness of breath or dyspnea, abnormal sense of smell, or abnormal sense of taste for 2 consecutive time points (2 and 3 months) after the start of treatment as the response variable. The explanatory variables will be treatment group, presence or absence of COVID-19 vaccination history, severity of COVID-19 symptoms (14 symptoms) at the time of consent (less than 9 points, 9 points or more), age (continuous variable), sex, and BMI (less than 25, 25 or more). The adjusted risk ratio will be calculated and compared with the placebo group.

Analysis Item: The proportion of subjects who have at least one of the following symptoms: malaise (fatigue), shortness of breath or dyspnea, abnormal sense of smell, or abnormal sense of taste for 2 consecutive time points (2 and 3 months) after the start of treatment, (with the symptoms being considered related or of unknown relation to COVID-19). Analysis Population: FAS Analysis Method: A modified Poisson regression model will be applied, using the presence or absence of at least one of the symptoms of malaise (fatigue), shortness of breath or dyspnea, abnormal sense of smell, or abnormal sense of taste, judged by the subjects themselves as being related or of unknown relation to COVID-19, for 2 consecutive time points (2 and 3 months) after the start of treatment as the response variable. The explanatory variables will be treatment group, presence or absence of COVID-19 vaccination history, severity of COVID-19 symptoms (14 symptoms) at the time of consent (less than 9 points, 9 points or more), age (continuous variable), sex, and BMI (less than 25, 25 or more). The adjusted risk ratio will be calculated and compared with the placebo group.

Analysis Item: The proportion of subjects who have each of the following symptoms: malaise (fatigue), shortness of breath or dyspnea, abnormal sense of smell, and abnormal sense of taste for 2 consecutive time points (2 and 3 months) after the start of treatment. Analysis Population: FAS Analysis Method: A modified Poisson regression model will be applied, using the presence or absence of each individual symptom of malaise (fatigue), shortness of breath or dyspnea, abnormal sense of smell, and abnormal sense of taste for 2 consecutive time points (2 and 3 months) after the start of treatment as the response variables. The explanatory variables will be treatment group, presence or absence of COVID-19 vaccination history, severity of COVID-19 symptoms (14 symptoms) at the time of consent (less than 9 points, 9 points or more), age (continuous variable), sex, and BMI (less than 25, 25 or more). The adjusted risk ratio will be calculated and compared with the placebo group.

Analysis Item: The proportion of subjects who have each of the following symptoms: malaise (fatigue), shortness of breath or dyspnea, abnormal sense of smell, and abnormal sense of taste for 2 consecutive time points (2 and 3 months) after the start of treatment, (with the symptoms being considered related or of unknown relation to COVID-19). Analysis Population: FAS Analysis Method: A modified Poisson regression model will be applied, using the presence or absence of each individual symptom of malaise (fatigue), shortness of breath or dyspnea, abnormal sense of smell, and abnormal sense of taste, judged by the subjects themselves as being related or of unknown relation to COVID-19, for 2 consecutive time points (2 and 3 months) after the start of treatment as the response variables. The explanatory variables will be treatment group, presence or absence of COVID-19 vaccination history, severity of COVID-19 symptoms (14 symptoms) at the time of consent (less than 9 points, 9 points or more), age (continuous variable), sex, and BMI (less than 25, 25 or more). The adjusted risk ratio will be calculated and compared with the placebo group.

Analysis Item: The proportion of subjects who have at least one of the 14 COVID-19 symptoms (malaise (fatigue), body aches or muscle pain, headache, chills, feverishness, runny or stuffy nose, sore throat, cough, shortness of breath or dyspnea, nausea, vomiting, diarrhea, abnormal sense of smell, or abnormal sense of taste) at both 1 month and 3 months after the start of treatment, OR who have at least one of the following symptoms at 3 months: muscle weakness, decreased concentration/thinking ability, decreased problem-solving ability, memory loss (short- or long-term), insomnia, hair loss, palpitations or increased heart rate, joint pain, loss of appetite, dizziness or abnormal balance, chest pain, or skin rash. Analysis Population: FAS Analysis Method: A modified Poisson regression model will be applied, using the presence or absence of either of the following as the response variable: "at least one of the 14 COVID-19 symptoms at both 1 month and 3 months after the start of treatment" OR "at least one of the other specified symptoms (muscle weakness, etc.) at 3 months". The explanatory variables will be treatment group, presence or absence of COVID-19 vaccination history, severity of COVID-19 symptoms (14 symptoms) at the time of consent (less than 9 points, 9 points or more), age (continuous variable), sex, and BMI (less than 25, 25 or more). The adjusted risk ratio will be calculated and compared with the placebo group.

Analysis Item: The proportion of subjects who have at least one of the 14 COVID-19 symptoms at both 1 month and 3 months after the start of treatment, OR who have at least one of the other symptoms (muscle weakness, etc.) at 3 months, (with the symptoms being considered related or of unknown relation to COVID-19). Analysis Population: FAS Analysis Method: A modified Poisson regression model will be applied, using the presence or absence of either of the following as the response variable: "at least one of the 14 COVID-19 symptoms, judged by the subjects themselves as being related or of unknown relation to COVID-19, at both 1 and 3 months after the start of treatment" OR "at least one of the other specified symptoms (muscle weakness, etc.), judged by the subjects themselves as being related or of unknown relation to COVID-19, at 3 months". The explanatory variables will be treatment group, presence or absence of COVID-19 vaccination history, severity of COVID-19 symptoms (14 symptoms) at the time of consent (less than 9 points, 9 points or more), age (continuous variable), sex, and BMI (less than 25, 25 or more). The adjusted risk ratio will be calculated and compared with the placebo group.

Analysis Item: The proportion of subjects who have at least one of the 14 COVID-19 symptoms (malaise (fatigue), body aches or muscle pain, headache, chills, feverishness, runny or stuffy nose, sore throat, cough, shortness of breath or dyspnea, nausea, vomiting, diarrhea, abnormal sense of smell, or abnormal sense of taste) at both 1 month and 3 months after the start of treatment. Analysis Population: FAS Analysis Method: A modified Poisson regression model will be applied, using the presence or absence of at least one of the 14 COVID-19 symptoms at both 1 month and 3 months after the start of treatment as the response variable. The explanatory variables will be treatment group, presence or absence of COVID-19 vaccination history, severity of COVID-19 symptoms (14 symptoms) at the time of consent (less than 9 points, 9 points or more), age (continuous variable), sex, and BMI (less than 25, 25 or more). The adjusted risk ratio will be calculated and compared with the placebo group.

Analysis Item: The proportion of subjects who have at least one of the 14 COVID-19 symptoms at both 1 month and 3 months after the start of treatment, (with the symptoms being considered related or of unknown relation to COVID-19). Analysis Population: FAS Analysis Method: A modified Poisson regression model will be applied, using the presence or absence of at least one of the 14 COVID-19 symptoms, judged by the subjects themselves as being related or of unknown relation to COVID-19, at both 1 month and 3 months after the start of treatment as the response variable. The explanatory variables will be treatment group, presence or absence of COVID-19 vaccination history, severity of COVID-19 symptoms (14 symptoms) at the time of consent (less than 9 points, 9 points or more), age (continuous variable), sex, and BMI (less than 25, 25 or more). The adjusted risk ratio will be calculated and compared with the placebo group.

Analysis Item: The proportion of subjects who have each of the 14 COVID-19 symptoms (malaise (fatigue), body aches or muscle pain, headache, chills, feverishness, runny or stuffy nose, sore throat, cough, shortness of breath or dyspnea, nausea, vomiting, diarrhea, abnormal sense of smell, or abnormal sense of taste) at both 1 month and 3 months after the start of treatment. Analysis Population: FAS Analysis Method: A modified Poisson regression model will be applied, using the presence or absence of each of the 14 COVID-19 symptoms at both 1 month and 3 months after the start of treatment as the response variables. The explanatory variables will be treatment group, presence or absence of COVID-19 vaccination history, severity of COVID-19 symptoms (14 symptoms) at the time of consent (less than 9 points, 9 points or more), age (continuous variable), sex, and BMI (less than 25, 25 or more). The adjusted risk ratio will be calculated and compared with the placebo group.

Analysis Item: The proportion of subjects who have each of the 14 COVID-19 symptoms at both 1 month and 3 months after the start of treatment, (with the symptoms being considered related or of unknown relation to COVID-19). Analysis Population: FAS Analysis Method: A modified Poisson regression model will be applied, using the presence or absence of each of the 14 COVID-19 symptoms, judged by the subjects themselves as being related or of unknown relation to COVID-19, at both 1 month and 3 months after the start of treatment as the response variables. The explanatory variables will be treatment group, presence or absence of COVID-19 vaccination history, severity of COVID-19 symptoms (14 symptoms) at the time of consent (less than 9 points, 9 points or more), age (continuous variable), sex, and BMI (less than 25, 25 or more). The adjusted risk ratio will be calculated and compared with the placebo group.

Analysis Item: The proportion of subjects who have at least one of the following symptoms at 3 months after the start of treatment: muscle weakness, decreased concentration/thinking ability, decreased problem-solving ability, memory loss (short- or long-term), insomnia, hair loss, palpitations or increased heart rate, joint pain, loss of appetite, dizziness or abnormal balance, chest pain, or skin rash. Analysis Population: FAS Analysis Method: A modified Poisson regression model will be applied, using the presence or absence of at least one of the specified symptoms at 3 months after the start of treatment as the response variable. The explanatory variables will be treatment group, presence or absence of COVID-19 vaccination history, severity of COVID-19 symptoms (14 symptoms) at the time of consent (less than 9 points, 9 points or more), age (continuous variable), sex, and BMI (less than 25, 25 or more). The adjusted risk ratio will be calculated and compared with the placebo group.

Analysis Item: The proportion of subjects who have at least one of the symptoms listed in the previous item at 3 months after the start of treatment, (with the symptoms being considered related or of unknown relation to COVID-19). Analysis Population: FAS Analysis Method: A modified Poisson regression model will be applied, using the presence or absence of at least one of the specified symptoms, judged by the subjects themselves as being related or of unknown relation to COVID-19, at 3 months after the start of treatment as the response variable. The explanatory variables will be treatment group, presence or absence of COVID-19 vaccination history, severity of COVID-19 symptoms (14 symptoms) at the time of consent (less than 9 points, 9 points or more), age (continuous variable), sex, and BMI (less than 25, 25 or more). The adjusted risk ratio will be calculated and compared with the placebo group.

Analysis Item: The proportion of subjects who have each of the following symptoms at 3 months after the start of treatment: muscle weakness, decreased concentration/thinking ability, decreased problem-solving ability, memory loss (short- or long-term), insomnia, hair loss, palpitations or increased heart rate, joint pain, loss of appetite, dizziness or abnormal balance, chest pain, or skin rash. Analysis Population: FAS Analysis Method: A modified Poisson regression model will be applied, using the presence or absence of each of the specified symptoms at 3 months after the start of treatment as the response variables. The explanatory variables will be treatment group, presence or absence of COVID-19 vaccination history, severity of COVID-19 symptoms (14 symptoms) at the time of consent (less than 9 points, 9 points or more), age (continuous variable), sex, and BMI (less than 25, 25 or more). The adjusted risk ratio will be calculated and compared with the placebo group.

Analysis Item: The proportion of subjects who have each of the symptoms listed in the previous item at 3 months after the start of treatment, (with the symptoms being considered related or of unknown relation to COVID-19). Analysis Population: FAS Analysis Method: A modified Poisson regression model will be applied, using the presence or absence of each of the specified symptoms, judged by the subjects themselves as being related or of unknown relation to COVID-19, at 3 months after the start of treatment as the response variables. The explanatory variables will be treatment group, presence or absence of COVID-19 vaccination history, severity of COVID-19 symptoms (14 symptoms) at the time of consent (less than 9 points, 9 points or more), age (continuous variable), sex, and BMI (less than 25, 25 or more). The adjusted risk ratio will be calculated and compared with the placebo group.

Analysis Item: The proportion of subjects who have not returned to their usual state of health as before COVID-19 at 3 months after the start of treatment. Analysis Population: FAS Analysis Method: A modified Poisson regression model will be applied, using whether or not the subject has returned to their usual state of health as before COVID-19 at 3 months after the start of treatment as the response variable. The explanatory variables will be treatment group, presence or absence of COVID-19 vaccination history, severity of COVID-19 symptoms (14 symptoms) at the time of consent (less than 9 points, 9 points or more), age (continuous variable), sex, and BMI (less than 25, 25 or more). The adjusted risk ratio will be calculated and compared with the placebo group.

Analysis Item: The amount of change from baseline in QOL at 3 months and 6 months after the start of treatment, respectively. Analysis Population: FAS Analysis Method: The change from baseline in utility values calculated from the EQ-5D-5L will be summarized for each group at each time point.

Analysis Item: The amount of change from baseline in work productivity score at 3 months and 6 months after the start of treatment, respectively. Analysis Population: FAS Analysis Method: The change from baseline in work productivity scores will be summarized for each group at each time point.

- - 1. Subgroup analysis for efficacy endpoints

Subgroup analysis by allocation factors (vaccination history, severity of COVID-19 symptoms (14 symptoms) at the time of consent (<9 points, ≥9 points), age, gender, BMI, and history of SARS-CoV-2 infection).

- - 1. Safety endpoints

Analysis Items ：Adverse Events, Diseases, etc.

Analysis population: SAS

Analysis method: The number of cases and the number of cases by organ category (SOC) and basic term (PT) in the Japanese version of the ICH International Glossary of Drugs (MedDRA) are tabulated for each incidence group, and the incidence rate is calculated. The Clopper-Pearson method will be used to calculate 95% confidence intervals for these incidence rates. Adverse events that are judged to have a causal relationship with the investigational drug are classified as adverse events and counted in the same manner as adverse events. In addition, the timing of occurrence, severity, treatment with the study drug, and outcome will be summarized by SOC and PT. Diseases, etc., will be tabulated in the same manner.

- 1. Criteria for interim analysis and early discontinuation

An interim analysis will be conducted once the total number of FAS patients who can be analyzed for the primary efficacy endpoint reaches 909, with the aim of discontinuing the study if the efficacy is clearly superior to that expected. An interim analysis may not be performed if a certain number of patients have already been enrolled at the time of the interim analysis.

Interim analyses will be performed by statistical analysts independent of the study; based on the results of the analyses described in Section 13.3.4, the Independent Data Monitoring Committee will make a recommendation for discontinuation of efficacy or continuation of the study. Enrollment of study subjects will remain open while the interim analysis is being conducted.

The O'Brien & Fleming type will be used to adjust for the multiplicity associated with the intermediate analysis.

The standard criteria for conducting interim analysis, such as the timing for deciding whether or not to conduct an interim analysis, and the criteria for deciding to discontinue efficacy, should be specified in advance in a separate procedure document and fixed prior to conducting an interim analysis. In addition, an interim analysis procedure document that specifies procedures and methods for conducting interim analysis under strict control of information under unblinded conditions, such as the scope of information disclosure for discontinuation of efficacy or recommendation to continue the study, should be prepared and fixed before conducting the interim analysis.

- 1. Change in statistical analysis plan

If any changes or additions to the analyses occur after the start of this study, the principal investigator shall consider their appropriateness and impact on the evaluation of this study, discuss them with the person in charge of statistical analysis, revise the research protocol, and explain in the summary report of this study the circumstances that led to the changes in the analysis plan.

1. Quality Control and Quality Assurance
   1. Quality Control Policy

This research will be conducted in compliance with the Clinical Research Act and its implementing regulations. Therefore, a quality control policy will be established to meet the requirements of these laws and regulations.

- 1. Quality Targets

Ensure that documentation required by the Clinical Research Act is prepared and maintained, and that the protection of research subjects is adhered to. A dropout rate of 20% will be allowed as the number of study subjects for whom the primary endpoint can be analyzed.

- 1. monitoring

In order to ensure that the clinical research is properly conducted from the perspective of ensuring the credibility of the clinical research and protecting the subjects of the clinical research, the principal investigator shall designate a person in charge of monitoring the progress of the research and whether the research is being conducted in accordance with the regulations and research protocol, and shall prepare a monitoring protocol. (2) The monitoring procedure is to be prepared and the monitoring is to be carried out in accordance with the monitoring manual.

The person in charge of monitoring shall conduct monitoring in accordance with the monitoring procedures and prepare a record of the monitoring (monitoring report).

The principal investigator or the administrator of the institution ensures that the monitoring personnel have access to the source documents.

- 1. audit

In order to ensure the credibility of the clinical research and the reliability of the data collected through the clinical research from the perspective of protecting the subjects of the clinical research, the principal investigator shall designate a person in charge of auditing and have him/her conduct an audit after preparing an audit protocol to determine whether the research was conducted in accordance with the legal regulations, etc. and the research protocol.

The Principal Investigator shall not allow those involved in the conduct of this research and its monitoring to perform audits.

The person in charge of the audit shall conduct the audit in accordance with the Audit Protocol.

The principal investigator or the administrator of the institution ensures that the person in charge of the audit has access to the source documents.

- 1. Response to investigations by regulatory authorities, etc.

The principal investigator or the administrator of the institution shall accept any request for investigation by the Authorized Clinical Research Review Committee or its designee, and by the Minister of Health, Labour and Welfare or its designee, and shall ensure that the source documents and other necessary materials are available for inspection.

- 1. incompatible
     1. Definition of Nonconformity

Noncompliance refers to non-compliance with the Enforcement Regulations of the Clinical Research Act, research protocols, procedure manuals, etc., and falsification or fabrication of research data. In managing nonconformity, the following procedures will be used.

- - 1. Critical Nonconformity

Serious non-compliance refers to anything that affects the human rights or safety of research subjects in clinical research and the progress of the research or the reliability of the results. For example, it refers to non-compliance with selection/exclusion criteria, discontinuation criteria, concomitant use of prohibited therapies, etc., and does not include non-compliance with the research protocol in order to avoid immediate danger to the research subjects in the clinical research or for other medically unavoidable reasons.

When the Principal Investigator becomes aware of a serious noncompliance, he/she must obtain the opinion of the Accredited Clinical Research Review Committee.

- - 1. Nonconformity Management Procedures

The principal investigator will document all nonconformities identified in this study.

Upon learning of the nonconformity, the research associate physician shall promptly report it to the principal investigator.

When the principal investigator becomes aware of a nonconformity, he/she shall promptly report it to the administrator of the implementing medical institution and notify the principal investigator.

The Principal Investigator shall promptly inform the other principal investigators of the occurrence of nonconformities, and shall make regular reports to the Accredited Clinical Research Review Committee regarding the occurrence of nonconformities and subsequent actions.

1. ethical consideration
   1. Rules and Regulations to be complied with

This research will be conducted in accordance with the ethical principles based on the Declaration of Helsinki, and in compliance with the Clinical Research Act, the enforcement regulations of the Act, and other relevant notices. The Principal Investigators and Research Assignments will conduct the protocol treatment in compliance with this research protocol.

- 1. Approval by an accredited clinical research review committee and the administrator of the implementing medical institution

The appropriateness of conducting this study will be reviewed and approved by an accredited clinical research review committee and approved by the administrator of the implementing medical institution, after the jRCT is published.

- 1. Cost sharing for research subjects in this study

1. The study drug will be provided free of charge by Shionogi, so there will be no financial burden on the research subjects.
2. Up to 10,000 yen will be transferred to the account designated by the research subject as a reward for the number of online interviews and inputs to the Study Concierge (2,000 yen each for the online interview at registration and after one week, and 1,500 yen each for inputs after one month, three months, and six months). (2,000 yen each for the online interview at registration and the online interview one week later; 1,500 yen each for the online interview one, three, and six months later; and 500 yen each for the online interview two, four, and five months later).
   1. Consent Explanatory Document and Consent of Study Subjects
      1. Procedure for Obtaining Consent

When obtaining consent from research subjects, the principal investigator shall prepare a consent explanation document and obtain approval from an accredited clinical research review committee.

The principal investigator or a research associate physician will explain the contents of the consent explanation document approved by an accredited clinical research review committee regarding this research to the research subjects in an online interview, and after confirming that the research subjects fully understand the contents of this research, they will be asked to participate in this research.

If the research subject agrees, an electronic signature by the research subject himself/herself is obtained on the consent form using MIROHA's eConsent function on the research subject's smartphone.

After confirming that the research subject has electronically signed the consent form, the principal investigator or research assistant physician will electronically sign the consent form using MIROHA's eConsent function. After confirming that both parties have electronically signed the consent form, the principal investigator or the research assistant physician will contact the research subject in person via e-mail with a link to download the consent document and the pdf file of the consent form, and will also store the pdf file of the consent form. The electronically signed consent form stored in MiROHA shall be considered the original.

However, if the research subject who received the explanation agrees to participate in the research but has difficulty signing the consent form electronically using the eConsent function of MiROHA, the consent form shall be signed by the research subject and the date of signature after confirming that a record of the research subject's review of the consent document and pre-signed consent form is on MiROHA. The consent document shall be signed by the Principal Investigator or a Research Assigning Physician at after confirming that a written document (in any format) with the subject's signature and the date of signature is available in MiROHA and is reviewed by the Research Subject during the online interview. In this case, a consent form (in paper form) signed by the principal investigator or a subinvestigator who provided the explanation to the research subject will be sent to the research subject at a later date, and the consent form signed and returned by the research subject will be retained as the original.

Research subjects may withdraw their consent of their own free will at any time, even after they have given their consent to participate in this study. If the subject wishes to withdraw consent, the subject should contact the principal investigator or a research associate by telephone or e-mail. The principal investigator or research associate physician will record the withdrawal of consent in MIROHA.

If the principal investigator or a subinvestigator revises the consent document regarding information that may affect the research subject's willingness to participate in the research for which consent has already been obtained, he/she will explain this to the research subject and obtain consent again using the revised consent document that has been reviewed and approved by an accredited clinical research review committee.

- - 1. Items to be included in the consent document

The consent document shall include the following

1. The name of the research and the approval of the administrator of the implementing medical institution for the conduct of said research, and the submission of an implementation plan to the Minister of Health, Labour and Welfare.
2. Name of the medical institution and the name and title of the principal investigator (including the name of the collaborating institution and the name of the principal investigator)
3. Purpose and Significance of this Study
4. Outline of Pharmaceuticals, etc.
5. Methods (including the purpose of use of information obtained from research subjects) and duration of this research
6. Reason for selection as research subject
7. Burden and anticipated risks and benefits to the research subjects
8. That consent to conduct or continue this research may be withdrawn at any time.
9. (2) That the research subjects will not be treated unfavorably by refusing to consent to the implementation or continuation of this research or by withdrawing their consent.
10. Methods of Disclosure of Information on this Study
11. (iii) a statement that the Research Subjects, etc. may, upon request of the Research Subjects, etc., obtain or inspect the research protocol and materials related to the methods of this research to the extent that this does not hinder the protection of the personal information of other Research Subjects, etc. or the securing of originality of the said research, and the method of obtaining or inspecting such materials.
12. Handling of personal information, etc. (including the method, if any, by which specific individuals cannot be identified)
13. Methods of information storage and disposal
14. Sources of funding for this research, etc., conflicts of interest related to research at the research institution and personal earnings, etc., and the status of conflicts of interest related to research by the researcher and others.
15. (2) Responding to consultations, complaints, and inquiries from research subjects, etc. and other parties concerned
16. Financial Burden or Gratuities to Research Subjects, etc.
17. Existence and details of other treatment methods and the anticipated benefits and disadvantages of other treatment methods.
18. Existence or non-existence of Compensation for Study-Related Injury caused by this research and the details of such compensation
19. (Matters to be reviewed by the Accredited Clinical Research Review Committee that conducts the business of reviewing and giving an opinion on the Specified Clinical Research and other matters concerning the Accredited Clinical Research Review Committee pertaining to said Specified Clinical Research)
20. (Contents of the Agreement stipulated in Article 32 of the Clinical Research Act)
21. Other matters necessary for the implementation of specific clinical research
    1. Consultation service from research subjects

Questions from the research subjects and their concerned parties will be handled by the principal investigator or a research associate.

- 1. Anticipated benefits and disadvantages to the research subjects
     1. Projected Profit

In all study subjects, symptomatic treatment for COVID-19 symptoms will be provided as needed, thus providing the same level of therapeutic benefit as in usual medical care. Since information on post-illness symptoms, quality of life, and labor productivity will be collected according to the study protocol, study subjects will have a more detailed picture of their health status, etc., than in usual medical care.

In addition, a detailed study of the therapeutic effect of encitrelvir fumarate on COVID-19 in this study may allow for future evaluation of more optimal treatment options.

- - 1. Anticipated disadvantages

The physical and mental burden on study subjects may increase compared to usual medical care, as they enter answers about post-illness symptoms, quality of life, and work productivity into an application installed on their smartphones.

Taking encitrelvir may cause the side effects listed in section 10.6.

- - 1. Comprehensive evaluation of benefits and disadvantages and measures to minimize disadvantages

The adverse effects of encitrelvir are transient, no serious adverse reactions have been reported, and the disadvantages are unlikely to outweigh the benefits. In order to minimize the disadvantages, safety confirmation based on online interviews will be conducted after completion of dosing, and signs of adverse events, etc. will be investigated. Prior to participation in the study, the research subjects will be fully informed and their willingness to participate will be confirmed. The principal investigator and co-principal investigator, Shionogi, will collect safety information and take appropriate actions such as revising the research protocol as necessary.

- 1. Confidentiality of research subjects (protection of personal information)

The contract research organization shall explain and obtain consent from patients when they enter their personal information (name, cell phone number, e-mail address, address, etc.) on the recruitment website in order for the contract research organization to view the personal information entered by the patients on the recruitment website for the purpose of conducting the contract research. The contract research organization will strictly manage the storage of patients' personal information to prevent it from being leaked outside the organization.

When handling raw data and consent forms related to the implementation of this research, we will give due consideration to the protection of the confidentiality of research subjects. Research subjects who have given their consent will be given a research subject identification code. The research subject identification code will consist of numerical symbols that are unrelated to information that can identify specific individuals, such as initials and medical record IDs, etc. The research subject identification code will be used when preparing documents related to this study, such as case report forms, so that specific individuals cannot be identified. The principal investigator will strictly manage the storage of correspondence sheets, etc. to prevent the leakage of personal information, such as the names of research subjects, to outside parties. When publishing the results of the research, information that can identify research subjects should not be included.

1. Compensation for Study-Related Injury

In the event that a health hazard occurs to a research subject as a result of the implementation of this research, the principal investigator and subinvestigator will recommend that the research subject consult a local physician and take other necessary measures so that the research subject can receive appropriate diagnosis, treatment, and necessary measures immediately.

The Principal Investigator shall purchase clinical research insurance (indemnity insurance) with the following coverage to compensate for damage to the health of research subjects, and shall provide compensation in accordance with the payment terms of the clinical research insurance (indemnity insurance).

(1) Compensation for death or disability of the research subject

(2) Medical expenses and medical allowances required for the treatment of health problems of Research Subjects. However, in this case, health insurance shall be applied to the treatment, and the compensation shall be the medical allowance for the Research Subject's out-of-pocket expenses for treatment and medical allowance for expenses other than treatment expenses.

The principal investigator or research assistant physician will state in the consent explanation document that compensation will be provided in the event of health problems, and will also prepare a document outlining the compensation and provide it to the research subjects at the time of consent explanation.

1. Discontinuation or termination of the entire clinical research
   1. Criteria for discontinuation

The Principal Investigator will discontinue or suspend the research as necessary if any of the following apply

1. When new significant information is obtained that may adversely affect the safety of research subjects or the conduct of this study, such as when anticipated adverse events (e.g., illnesses) significantly exceed those anticipated at the time of planning.
2. When it is judged to be extremely difficult to achieve the target number of research subjects, such as when the enrollment of research subjects is significantly slower than planned
3. If an opinion is received from an accredited clinical research review committee that this study should be discontinued
4. Other circumstances necessitating the discontinuation or suspension of this research
   1. Abort Procedure

The Principal Investigator will promptly notify the Principal Investigator of the decision to discontinue the entire study when it is found that the discontinuation criteria are met.

The principal investigator or subinvestigator will explain the discontinuation of the research to the research subjects and, to the extent possible, will conduct the observations, examinations, and evaluations planned for this research and take alternative treatment or other medical measures as necessary.

The Principal Investigator shall submit a notice of discontinuation to the Authorized Clinical Research Review Committee and notify the Minister of Health, Labour and Welfare within 10 days of the decision to discontinue the entire study.

- 1. Termination Criteria

The date when the Principal Investigator publishes the summary of the summary report by recording it in the jRCT shall be the date when this study is terminated.

1. Disclosure of research information and publication of results
   1. Register your study

Prior to the conduct of this study, the participants will be enrolled in the jRCT. The research protocol will be updated as appropriate according to changes in the research protocol and the progress of the study.

- 1. Publication of Research Results
     1. Key Evaluators Report

The principal investigator shall prepare a report on the primary endpoints within one year, in principle, from the date the period for collecting data on the primary endpoints ends, obtain the opinion of the accredited clinical research review committee, and submit the report to the administrator of the implementing medical institution. The report will be made public by recording it in jRCT within one month from the date on which the accredited clinical research review committee expressed its opinion.

The principal investigator will promptly report to the administrator of the implementing medical institution that he/she has made a public announcement and provide information to other principal investigators to that effect. The other principal investigators will promptly report the details of such information to the administrator of the implementing medical institution.

Since the preparation of the main evaluation item report for this study is scheduled to coincide with the preparation of the summary report, the main evaluation item report will be deemed to have been prepared with the preparation of the summary report.

- - 1. general report

The Principal Investigator shall prepare a summary report and summary of the summary report, in principle, within one year from the date of completion of the period for collecting data on all assessment items.

The general report shall include, at a minimum, the following

(1) Background information on subjects of clinical research (age, gender, etc.)

(2) Information on the progress according to the design of the clinical research (e.g., number of subjects)

(3) Summary of outbreaks of diseases, etc.

(4) Data analysis and results of primary and secondary endpoints

The principal investigator shall ask the authorized clinical research review committee for its opinion on the summary report and its outline, and submit it to the administrator of the implementing medical institution. Within one month from the date on which the accredited clinical research review committee has expressed its opinion, the summary of said summary report, research plan and statistical analysis plan (if prepared) shall be made public by recording them in the jRCT.

The principal investigator will promptly report to the administrator of the implementing medical institution that he/she has made a public announcement and provide information to other principal investigators to that effect. The other principal investigators will promptly report the details of such information to the administrator of the implementing medical institution.

- - 1. Publication of conferences, etc.

The results obtained from this research will be promptly made public through conference presentations and article submissions. When publishing the results, will take necessary measures to protect the human rights and interests of the research subjects and other related parties before publishing them. Conference presenters and authors of papers will be selected based on their contribution to this research.

1. Change Management
   1. Changes to documents approved by an accredited clinical research review committee

If any changes are made from the documents approved by the approved Clinical Research Review Committee, an application for change should be submitted to the approved Clinical Research Review Committee. When an opinion is given by the Accredited Clinical Research Review Committee, promptly report the contents of the opinion to the administrator of the implementing medical institution.

- 1. Change of implementation plan

(2) When the Principal Investigator changes the implementation plan (except for minor changes specified by an Ordinance of the Ministry of Health, Labour and Welfare), the Principal Investigator shall obtain the opinion of the Certified Clinical Research Review Committee as stated in said implementation plan, and submit the amended implementation plan and a notification form according to Form 2 in advance. (2) When a principal investigator intends to change the implementation plan (except for minor changes specified by an Ordinance of the Ministry of Health, Labour and Welfare), he/she shall obtain the opinion of the approved clinical research review committee described in said implementation plan and submit the amended implementation plan and a notification form according to Form 2 in advance.

- 1. Minor changes to the implementation plan

(2) When the Principal Investigator makes a minor change to the research plan in accordance with Article 42 of the Ordinance for Enforcement of the Clinical Research Act, the Principal Investigator shall, within 10 days from the date of such change, notify the details of the change to the Authorized Clinical Research Review Committee described in the relevant research plan, and notify the Minister of Health, Labour and Welfare of such change.

1. conflict of interest
   1. Sources of funding for this study

The project will be funded by Shionogi .

This study is conducted from a medical perspective and is not for the benefit or convenience of the company concerned.

- 1. Conflicts of Interest Management

This study will be conducted at each implementing medical institution after receiving fact-checking and preparing a conflict of interest management plan, and will be managed appropriately by obtaining the opinions of an accredited clinical research review committee.

The conflict of interest situation for this study is shown in Appendix 1.

1. Periodic reports to the Accredited Clinical Research Review Committee and the Minister of Health, Labour and Welfare

The Principal Investigator shall report the following matters concerning the implementation of the Specified Clinical Research to the Administrator of the Operating Medical Institution, and then to the Authorized Clinical Research Review Committee and the Minister of Health, Labour and Welfare.

The principal investigator will promptly inform the other principal investigators that he/she has made a periodic report to an accredited clinical research review committee. The other principal investigators will promptly report the details of such information to the administrator of the implementing medical institution.

- 1. Periodic reports to the Accredited Clinical Research Review Board
     1. Matters to be reported in periodic reports

(1) Number of research subjects who participated in this study

(2) Outbreak and subsequent progress of diseases, etc. related to this research

(3) Occurrence of non-compliance with this Ministerial Ordinance or the Research Protocol pertaining to this research and subsequent actions taken

(4) Evaluation of the safety and scientific validity of the study

(5) Matters concerning the involvement of manufacturers and distributors of pharmaceuticals, etc. in this research

- - 1. Periodic Reporting Period

Periodic reports to the Authorized Clinical Research Review Committee shall, in principle, be made every year, starting from the date of submission of the implementation plan to the Minister of Health, Labour and Welfare, and within two months after the expiration of the relevant period.

- 1. Periodic reports to the Minister of Health, Labor and Welfare
     1. Matters to be reported in periodic reports

(1) Name of the approved clinical research review committee as stated in the implementation plan

(2) Appropriateness of the continuation of this study by the approved clinical research review committee

(3) Number of research subjects who participated in this study

- - 1. Periodic Reporting Period

Periodic reports to the Minister of Health, Labour and Welfare shall be made within one month from the date on which the Accredited Clinical Research Review Committee has expressed its opinion.

1. Methods of storage and disposal of materials, records, etc.
   1. Retention of original documents

Source Materials" means the original records and data concerning clinical findings, observations, and other activities obtained through the application and treatment of Drugs and Other Products to Research Subjects.

The principal investigator or administrator of the institution will retain the following materials, including source documents and documents specific to this study, for investigation or audit by an accredited clinical research review committee and regulatory authorities or their designees.

These materials include signed and dated consent forms pdf files, etc.

In addition, the principal investigator or administrator of the institution will retain the essential documents to be kept until the date five years have elapsed after the cessation or termination of the study .

When the principal investigator amends any of the above documents or records, he/she shall record the name of the person who made the amendment and the date of the amendment, and retain the amended record together with the amended record.

- 1. Retention of documents of record as required by law

The principal investigator will properly store records and materials related to the deliberations of this study for a period of five years after the discontinuation or termination of this study to prevent leakage, mix-up, theft, or loss of such records and materials. Documents to be kept for this research are as follows.

(1) Documents that identify the research subject(s)

(2) Documents describing matters relating to medical treatment and examination of research subjects

(3) Documents describing matters related to participation in this study

(4) Documents describing matters relating to the administration of the drug, etc. under study to the research subjects.

(5) Documents describing matters pertaining to the review opinion work on this research received from the accredited clinical research review committee.

(6) Documents pertaining to the research protocol, implementation plan, explanation to research subjects, and their consent

(7) Summary report and other documents (or copies) prepared by the principal investigator pursuant to the provisions of the Enforcement Regulations of the Clinical Research Act

(8) Documents related to monitoring and auditing

(9) Original documents, etc., excluding (1)～ (4) above

(10) Contract for the implementation of this research

(11) Documents describing an outline of the drugs and other products to be used in the study

(12) Records (quantity and date) of acquisition of pharmaceuticals and other products subject to this study

(13) Records of the disposal of pharmaceuticals and other products that were the subject of this research (if disposed of)

(14) Other documents necessary to conduct this research in addition to the above

When amending these records, the principal investigator shall record the name of the amender and the date of the amendment, and retain the amended records together with the amended records.

- 1. Secondary Use of Information

Information obtained in this study may be used for different research purposes (secondary use). This should be stated in the consent and explanation document, and consent should be obtained after explaining this to the research subjects. In the case of secondary use, a new research protocol should be prepared, and the research should be conducted after obtaining approval from the Ethical Review Committee that is to hear opinions on the research in question.

- 1. Disposal Procedures and Methods

When disposing of information obtained from research subjects in this study, records and materials related to the deliberations of this study, the principal investigator will take necessary measures to ensure that specific individuals cannot be identified.

The administrator of the implementing medical institution shall retain the information, records, and materials to be retained by the principal investigator until notified by the investigator that such retention is no longer necessary.

1. Ownership of Data and Results

The attribution of the results of this research shall be stipulated in the contract between the fund provider, Shionogi & Co. and The University of Osaka﻿. After the completion of this research, a clinical research completion report shall be submitted to the fund provider, Shionogi & Co. In addition, if the Principal Investigator wishes to release information obtained in this study, he/she shall obtain the consent of Shionogi & Co. in accordance with the contract.

1. Committees, etc. to be established in this study
   1. Independent Data Monitoring Committee

An Independent Data Monitoring Committee will be established to evaluate the need for efficacy discontinuation following the results of the interim analysis of this study.

Independent Data Monitoring Committee members will be appointed on the basis of their research history and achievements in the area of this study, experience in the development of similar drugs, and ability to ensure independence from parties involved in the conduct of this clinical research (e.g., principal investigators and other persons engaged in this research, certified clinical research review committee members, and administrators of the implementing healthcare institutions).

1. References

| [1] | Health and Labour Sciences Special Research Project, Fukunaga Group, "Fundamental study to understand the actual situation of long-term complications of novel coronavirus infection (COVID-19) and to elucidate the pathophysiology: A comprehensive research report," 1 6 2022. [Online]. Available: https://www.mhlw.go.jp/content/10900000/000945990.pdf. [Accessed on: 30 5 2023]. |
| --- | --- |
| [2] | MHLW Special Research Project, Yokoyama Group, "Investigation on the actual condition of COVID-19 sequelae (for patients with moderate or severe disease or more): A comprehensive research report," 1 6 2022. Available: https://www.mhlw.go.jp/content/10900000/000945990.pdf. [Accessed on: 30 5 2023]. |
| [3] | Department of Infection Control Medicine, The University of Osaka﻿ Graduate School of Medicine. et al, "Results of Post-Coronary Survey," 14 12 2022. [Online]. Available: https://www.city.toyonaka.osaka.jp/kenko/covid19_support/koronakouisyousien.files/tyousakekka3.pdf. [Accessed on: 30 5 2023]. |
| [4] | Ministry of Health, Labour and Welfare, "Survey of Antibody Availability of New-type Coronavirus Using Residual Blood Samples for Testing at the Second Blood Donation," [online]. Available: https://www.mhlw.go.jp/content/10906000/001070846.pdf. [Accessed on: 30 5 2023]. |
| [5] | Xie Y, et al. "Association of treatment with nirmatrelvir and the risk of post-COVID-19 condition," JAMA Intern Med. 2023;e 230743. doi: 10.1001/jamainternmed.2023.0743. |
| [6] | Ying, H., et al, "Encytrelvir fumarate may suppress COVID-19 post-affect symptoms (Long COVID)," Precision Medicine. 2023;6:291-299, 2023. |
| [7] | Tsampasian V, et al. "Risk factors associated with post-COVID-19 condition: a systematic review and meta-analysis," JAMA Intern Med. 2023;e230750. doi: 10.1001/jamainternmed.2023.0750. |

**Appendix 1 (Conflict of Interest Matters)**

Title of the study: of encitrelvir fumarate in the treatment of COVID-19 post-illness symptoms

Principal Investigator：Kenshi Kutsuna, Department of Infection Control, The University of Osaka﻿ Hospital

COI (involvement in research) regarding pharmaceutical companies, etc. involved in this research

| COI with Shionogi & Co. |
| --- |
| Provision of research funds, etc.  Provision of goods: test drugs  Provision of services: Provision of necessary information and planning for the preparation of various documents such as research protocols based on collaborative research agreements. |

COI to be disclosed between the subject drug pharmaceutical companies, etc. involved in this research and the principal investigators/associated investigators of the implementing medical institution.

| COI with Shionogi & Co. | |
| --- | --- |
| Name of medical institution | the number of people |
| The University of Osaka﻿ Hospital | 10 |

　　　　　　　　　　　　　　　　　　　　　　　　　　(as of November 26, 2024)
