# Supplementary material for: Research to evaluate safety and impact of long COVID intervention with Ensitrelvir for National Cohort (RESILIENCE Study): A protocol for a randomized, double-blind, placebo-controlled trial
Source: PLoS One. 2025 Nov 3;20(11):e0335609. doi: 10.1371/journal.pone.0335609 (PMC12582476; doi:10.1371/journal.pone.0335609)
Supplement: S2 File — The original full study protocol in Japanese. (DOCX) [file pone.0335609.s002.docx]

特定臨床研究計画書

エンシトレルビル フマル酸のCOVID-19罹患後症状に対する有効性の検証

Research Evaluation of Safety and Impact in Long COVID Intervention with Ensitrelvir for National Cohort Efficacy

（RESILIENCE study）

| 研究代表医師 | 忽那　賢志  大阪大学医学部附属病院　感染制御部  TEL：06-6879-5111 |
| --- | --- |
| 研究計画書番号 | COVID-ENT-001 |
| 作成日 | 2025年7月25日 |
| 版数 | 第4.0版 |

版管理

| 版番号 | 作成日・改訂日 |
| --- | --- |
| 第1版 | 2023年11月13日 |
| 第1.1版 | 2023年12月28日 |
| 第2.0版 | 2024年5月21日 |
| 第2.1版 | 2024年7月31日 |
| 第2.2版 | 2024年8月9日 |
| 第2.3版 | 2024年10月28日 |
| 第2.4版 | 2024年11月21日 |
| 第3.0版 | 2024年11月26日 |
| 第3.1版 | 2025年1月21日 |
| 第3.2版 | 2025年4月28日 |
| 第3.3版 | 2025年6月2日 |
| 第4.0版 | 2025年7月25日 |

**概要**

1. **臨床研究の目的及び内容**

| 研究の目的 | 軽症COVID-19患者を対象にして、エンシトレルビル フマル酸を5日間投与した時の罹患後症状におけるエンシトレルビルのプラセボに対する優越性を検証する。また、エンシトレルビルの安全性を評価する。 |
| --- | --- |
| 研究予定期間 | jRCT公表日～2026年12月31日 |
| 計画研究対象者数 | 登録する研究対象者数として合計2,000例  エンシトレルビル群：1,000例  プラセボ群：1,000例 |
| 試験デザイン | 多施設、無作為割付、二重盲検、並行群間比較試験 |
| 対象疾患名 | COVID-19 |
| 選択基準 | （１）核酸増幅法（PCR 法、LAMP法等）、抗原定性検査又は抗原定量検査で研究対象者紹介医療機関において COVID-19と診断された者  （２）同意取得時に新型コロナウイルス感染症（COVID-19）診療の手引き（第10.0版）での重症度分類が軽症の者  （３）発症から72時間以内に試験薬の服用開始が期待できる者  （４）研究対象者紹介医療機関での確定診断時の体温が37.0℃以上の者  （５）妊娠する可能性のある女性では、試験薬の服用期間中及び最終服用後2週間以内に性交渉を行う場合にパートナーと共に適切な避妊を行うことが可能な者  （６）同意取得時の年齢が18歳以上である者  （７）本研究への参加について文書による同意が本人から得られる者 |
| 除外基準 | （１）COVID-19の重症化リスクが高い者  （２）今回のCOVID-19を発症してから、又は同意取得日前15日以内にCOVID-19への効能効果を有する抗ウイルス薬（レムデシビル、モルヌピラビル、ニルマトレルビル／リトナビル、エンシトレルビル フマル酸等）、中和抗体薬（カシリビマブ／イムデビマブ、ソトロビマブ、チキサゲビマブ／シルガビマブ等）の投与を受けた者  （３）同意取得前2週間以内に抗IL-6抗体製剤（トシリズマブ等）、JAK阻害薬（バリシチニブ等。外用を除く）、内服、坐薬又は注射用のステロイド薬の投与を受けた者  （４）今回のCOVID-19を発症してからCOVID-19治療薬の臨床試験に参加したことがある者  （５）スマートフォンを利用した研究責任医師又は研究分担医師とのオンライン面談が不可能な者  （６）スマートフォンを利用した患者報告アウトカム収集システムへの入力が不可能な者  （７）エンシトレルビル製剤の成分に対し過敏症の既往歴のある患者  （８）エンシトレルビル フマル酸の併用禁忌となっている薬剤を投与中の者、又は試験薬の投与期間中及び最終投与後2週間以内に投与予定がある者  （９）腎機能又は肝機能障害のある患者で、コルヒチンを投与中の者  （１０）妊娠又は妊娠している可能性のある女性（妊娠の可能性については、月経歴、及び最近の性行為を確認したうえで判断する）  （１１）授乳中の女性  （１２） 重度の肝機能障害を有する者  （１３） 免疫不全患者及び透析中の者  （１４）他の感染症を併存している者  （１５） 他の介入研究に参加中の者又は本研究に参加したことのある者  （１６） 過去1年以内に医薬品の治験に参加したことがある者  （１７） その他の理由で、研究責任医師又は研究分担医師が研究対象者として不適当と判断した者 |
| プロトコル治療 | 1. エンシトレルビル群  エンシトレルビルとして1日目は375mgを、2日目から5日目は125mgを1日1回経口投与する。  2. プラセボ群  プラセボを1日目は3錠を、2日目から5日目は1錠を1日1回経口投与する。 |
| 中止基準 | 1. 研究対象者ごとの試験薬服用の中止  （１）有害事象が発現し、本研究の継続により研究対象者の健康に容認できないリスクが生じると研究責任医師又は研究分担医師が判断した場合  （２）研究対象者から試験薬服用をやめたいとの申し出があった場合  （３）エンシトレルビル フマル酸の併用禁忌となっている薬剤の投与が必要になった場合  （４）その他のCOVID-19治療薬投与や呼吸療法が必要になった場合、又は併存疾患のために当該疾患の効能も有するCOVID-19治療薬の投与が必要になった場合  （５）研究対象者として不適切であることが判明した場合  （６）女性研究対象者の妊娠が判明した場合  （７）その他、研究責任医師又は研究分担医師が介入を中止すべきであると判断した場合  2. 研究対象者ごとの研究の中止  （１）研究対象者から研究中止の申し出があった場合  （２）スマートフォンの故障等、研究対象者の都合で必要な観察の今後の実施が不可能であることが判明した場合  （３）追跡期間中に研究対象者がCOVID-19に再度罹患したことが判明した場合  （４）試験薬服用前に研究対象者として不適切であることが判明した場合  （５）その他、研究責任医師又は研究分担医師が研究を中止すべきであると判断した場合  3.　研究全体の中止  （１）予想される有害事象（疾病等）が計画時の想定を著しく超える場合等、研究対象者の安全性又は本研究の実施に悪影響を及ぼす可能性のある新たな重大な情報を入手した場合  （２）研究対象者の登録が計画と比較して著しく遅い場合等、目標とする研究対象者数を達成することが極めて困難であると判断される場合  （３）認定臨床研究審査委員会から本研究を中止すべき旨の意見を受けた場合  （４）その他、本研究の中止又は中断を必要とする状況が発生した場合 |
| 有効性主要評価項目 | 「治療開始から1ヵ月及び3ヵ月の2時点両方で、けん怠感（疲労感）、息切れ又は呼吸困難感、嗅覚の異常、味覚の異常のいずれかの症状がある」又は「3ヵ月時点で集中力・思考力の低下、課題解決力の低下、物忘れ（短期又は長期）のいずれかの症状がある」研究対象者の割合 |
| 有効性副次評価項目 | （１）「治療開始から1ヵ月及び3ヵ月の2時点両方で、けん怠感（疲労感）、息切れ又は呼吸困難感、嗅覚の異常、味覚の異常のいずれかの症状がある」又は「3ヵ月時点で集中力・思考力の低下、課題解決力の低下、物忘れ（短期又は長期）のいずれかの症状がある」研究対象者の割合（COVID-19と関連がある、又は関連不明）  （２）「治療開始から1ヵ月及び3ヵ月及び6ヵ月の3時点全てで、けん怠感（疲労感）、息切れ又は呼吸困難感、嗅覚の異常、味覚の異常のいずれかの症状がある」又は「6ヵ月時点で集中力・思考力の低下、課題解決力の低下、物忘れ（短期又は長期）のいずれかの症状がある」研究対象者の割合  （３）治療開始から1ヵ月及び3ヵ月及び6ヵ月の3時点全てで、けん怠感（疲労感）、息切れ又は呼吸困難感、嗅覚の異常、味覚の異常のいずれかの症状がある」又は「6ヵ月時点で集中力・思考力の低下、課題解決力の低下、物忘れ（短期又は長期）のいずれかの症状がある」研究対象者の割合（COVID-19と関連がある、又は関連不明）  （４）治療開始から1ヵ月及び3ヵ月の2時点両方で、けん怠感（疲労感）、息切れ又は呼吸困難感、嗅覚の異常、味覚の異常のいずれかの症状がある研究対象者の割合  （５）治療開始から1ヵ月及び3ヵ月の2時点両方で、けん怠感（疲労感）、息切れ又は呼吸困難感、嗅覚の異常、味覚の異常のいずれかの症状がある研究対象者の割合（COVID-19と関連がある、又は関連不明）  （６）治療開始から1ヵ月及び3ヵ月の2時点両方で、けん怠感（疲労感）、息切れ又は呼吸困難感、嗅覚の異常、味覚の異常の各症状がある研究対象者の割合  （７）治療開始から1ヵ月及び3ヵ月の2時点両方で、けん怠感（疲労感）、息切れ又は呼吸困難感、嗅覚の異常、味覚の異常の各症状がある研究対象者の割合（COVID-19と関連がある、又は関連不明）  （８）治療開始から3ヵ月時点で集中力・思考力の低下、課題解決力の低下、物忘れ（短期又は長期）のいずれかの症状がある研究対象者の割合  （９）治療開始から3ヵ月時点で集中力・思考力の低下、課題解決力の低下、物忘れ（短期又は長期）のいずれかの症状がある研究対象者の割合（COVID-19と関連がある、又は関連不明）  （10）治療開始から3ヵ月時点で集中力・思考力の低下、課題解決力の低下、物忘れ（短期又は長期）の各症状がある研究対象者の割合  （11）治療開始から3ヵ月時点で集中力・思考力の低下、課題解決力の低下、物忘れ（短期又は長期）の各症状がある研究対象者の割合（COVID-19と関連がある、又は関連不明）  （12）治療開始から3ヵ月時点でCOVID-19 罹患前同様の通常の健康状態に戻っておらず、COVID-19の14症状（けん怠感（疲労感）、体の痛み又は筋肉痛、頭痛、悪寒、熱っぽさ、鼻水もしくは鼻づまり、喉の痛み、咳、息切れ又は呼吸困難、吐き気、嘔吐、下痢、嗅覚の異常、味覚の異常）のいずれかの症状がある研究対象者の割合  （13）治療開始から3ヵ月時点でCOVID-19 罹患前同様の通常の健康状態に戻っておらず、神経学的4症状（集中力・思考力の低下、課題解決力の低下、物忘れ（短期又は長期）、不眠）のいずれかの症状がある研究対象者の割合  （14）治療開始から3ヵ月時点でCOVID-19 罹患前同様の通常の健康状態に戻っておらず、けん怠感（疲労感）、体の痛み又は筋肉痛、頭痛、悪寒、熱っぽさ、鼻水もしくは鼻づまり、喉の痛み、咳、息切れ又は呼吸困難、吐き気、嘔吐、下痢、嗅覚の異常、味覚の異常、筋力低下、集中力・思考力の低下、課題解決力の低下、物忘れ（短期又は長期）、不眠、脱毛、動悸又は心拍数の増加、関節痛、食欲不振、めまい又は平衡感覚の異常、胸痛、皮膚の発疹のいずれかの症状がある研究対象者の割合 |
| 有効性探索評価項目 | （１）「治療開始から2ヵ月後及び3ヵ月の2時点連続で、けん怠感（疲労感）、息切れ又は呼吸困難感、嗅覚の異常、味覚の異常のいずれかの症状がある」又は「3ヵ月時点で集中力・思考力の低下、課題解決力の低下、物忘れ（短期又は長期）のいずれかの症状がある」研究対象者の割合  （２）「治療開始から2ヵ月後及び3ヵ月の2時点連続で、けん怠感（疲労感）、息切れ又は呼吸困難感、嗅覚の異常、味覚の異常のいずれかの症状がある」又は「3ヵ月時点で集中力・思考力の低下、課題解決力の低下、物忘れ（短期又は長期）のいずれかの症状がある」研究対象者の割合（COVID-19と関連がある、又は関連不明）  （３）治療開始から2ヵ月及び3ヵ月の2時点連続で、けん怠感（疲労感）、息切れ又は呼吸困難感、嗅覚の異常、味覚の異常のいずれかの症状がある研究対象者の割合  （４）治療開始から2ヵ月及び3ヵ月の2時点連続で、けん怠感（疲労感）、息切れ又は呼吸困難感、嗅覚の異常、味覚の異常のいずれかの症状がある研究対象者の割合（COVID-19と関連がある、又は関連不明）  （５）治療開始から2ヵ月及び3ヵ月の2時点連続で、けん怠感（疲労感）、息切れ又は呼吸困難感、嗅覚の異常、味覚の異常の各症状がある研究対象者の割合  （６）治療開始から2ヵ月及び3ヵ月の2時点連続で、けん怠感（疲労感）、息切れ又は呼吸困難感、嗅覚の異常、味覚の異常の各症状がある研究対象者の割合（COVID-19と関連がある、又は関連不明）  （7）「治療開始から1ヵ月及び3ヵ月の2時点両方でCOVID-19の14症状（けん怠感（疲労感）、体の痛み又は筋肉痛、頭痛、悪寒、熱っぽさ、鼻水もしくは鼻づまり、喉の痛み、咳、息切れ又は呼吸困難、吐き気、嘔吐、下痢、嗅覚の異常、味覚の異常）のいずれかがある」又は「3ヵ月時点で筋力低下、集中力・思考力の低下、課題解決力の低下、物忘れ（短期又は長期）、不眠、脱毛、動悸又は心拍数の増加、関節痛、食欲不振、めまい又は平衡感覚の異常、胸痛、皮膚の発疹のいずれかの症状がある」研究対象者の割合  （8）「治療開始から1ヵ月及び3ヵ月の2時点両方でCOVID-19の14症状（けん怠感（疲労感）、体の痛み又は筋肉痛、頭痛、悪寒、熱っぽさ、鼻水もしくは鼻づまり、喉の痛み、咳、息切れ又は呼吸困難、吐き気、嘔吐、下痢、嗅覚の異常、味覚の異常）のいずれかがある」又は「3ヵ月時点で筋力低下、集中力・思考力の低下、課題解決力の低下、物忘れ（短期又は長期）、不眠、脱毛、動悸又は心拍数の増加、関節痛、食欲不振、めまい又は平衡感覚の異常、胸痛、皮膚の発疹のいずれかの症状がある」研究対象者の割合（COVID-19と関連がある、又は関連不明）  （9）治療開始から1ヵ月及び3ヵ月の2時点両方で COVID-19の14症状（けん怠感（疲労感）、体の痛み又は筋肉痛、頭痛、悪寒、熱っぽさ、鼻水もしくは鼻づまり、喉の痛み、咳、息切れ又は呼吸困難、吐き気、嘔吐、下痢、嗅覚の異常、味覚の異常）のいずれかの症状がある研究対象者の割合  （10）治療開始から1ヵ月及び3ヵ月の2時点両方で COVID-19の14症状（けん怠感（疲労感）、体の痛み又は筋肉痛、頭痛、悪寒、熱っぽさ、鼻水もしくは鼻づまり、喉の痛み、咳、息切れ又は呼吸困難、吐き気、嘔吐、下痢、嗅覚の異常、味覚の異常）のいずれかの症状がある研究対象者の割合（COVID-19と関連がある、又は関連不明）  （11）治療開始から1ヵ月及び3ヵ月の2時点両方で、 COVID-19の14症状（けん怠感（疲労感）、体の痛み又は筋肉痛、頭痛、悪寒、熱っぽさ、鼻水もしくは鼻づまり、喉の痛み、咳、息切れ又は呼吸困難、吐き気、嘔吐、下痢、嗅覚の異常、味覚の異常）の各症状がある研究対象者の割合  （12）治療開始から1ヵ月及び3ヵ月の2時点両方で、 COVID-19の14症状（けん怠感（疲労感）、体の痛み又は筋肉痛、頭痛、悪寒、熱っぽさ、鼻水もしくは鼻づまり、喉の痛み、咳、息切れ又は呼吸困難、吐き気、嘔吐、下痢、嗅覚の異常、味覚の異常）の各症状がある研究対象者の割合（COVID-19と関連がある、又は関連不明）  （13）治療開始から3ヵ月時点で筋力低下、集中力・思考力の低下、課題解決力の低下、物忘れ（短期又は長期）、不眠、脱毛、動悸又は心拍数の増加、関節痛、食欲不振、めまい又は平衡感覚の異常、胸痛、皮膚の発疹のいずれかの症状がある研究対象者の割合  （14）治療開始から3ヵ月時点で筋力低下、集中力・思考力の低下、課題解決力の低下、物忘れ（短期又は長期）、不眠、脱毛、動悸又は心拍数の増加、関節痛、食欲不振、めまい又は平衡感覚の異常、胸痛、皮膚の発疹のいずれかの症状がある研究対象者の割合（COVID-19と関連がある、又は関連不明）  （15）治療開始から3ヵ月時点で筋力低下、集中力・思考力の低下、課題解決力の低下、物忘れ（短期又は長期）、不眠、脱毛、動悸又は心拍数の増加、関節痛、食欲不振、めまい又は平衡感覚の異常、胸痛、皮膚の発疹の各症状がある研究対象者の割合  （16）治療開始から3ヵ月時点で筋力低下、集中力・思考力の低下、課題解決力の低下、物忘れ（短期又は長期）、不眠、脱毛、動悸又は心拍数の増加、関節痛、食欲不振、めまい又は平衡感覚の異常、胸痛、皮膚の発疹の各症状がある研究対象者の割合（COVID-19と関連がある、又は関連不明）  （17）治療開始から3ヵ月時点でCOVID-19 罹患前同様の通常の健康状態に戻っていない研究対象者の割合  （18）治療開始から3ヵ月後及び6ヵ月後のそれぞれの時点における、 QOL のベースライン からの変化量  （19）治療開始から3ヵ月後及び6ヵ月後のそれぞれの時点における、労働生産性のベースラインからの変化量 |
| 安全性評価項目 | （１）有害事象の発現例数及び件数とその割合  （２）重篤な有害事象の発現例数及び件数とその割合 |

1. **研究の概略図（シェーマ）**

6ヵ月後

↓

服用5日目

↓

服用開始

↓

罹患後症状の確認

割付

エンシトレルビル フマル酸群

罹患後症状の確認

プラセボ

1. **観察・検査・評価のスケジュール**

研究責任医師又は研究分担医師は、「観察・検査・評価スケジュール」に従って、データを収集する。

|  | 前観察期 | 治療期 | | 追跡期間 | | | | | | | |
| --- | --- | --- | --- | --- | --- | --- | --- | --- | --- | --- | --- |
|  | 登録時 | 試験薬 服用 開始日 | 1週後^*2^ | | 1ヵ月後 | 2ヵ月後 | 3ヵ月後 | 4ヵ月後 | 5ヵ月後 | ６ヵ月後 | 中止時 |
| Visit | 1 | - | 2 | | 3 | 4 | 5 | 6 | 7 | 8 |  |
| Day |  | 0 | 7 | | 28 | 56 | 84 | 112 | 140 | 168 |  |
| 許容範囲 | －^*1^ | ― | －2～+5 | | ±7 | ±7 | ±7 | ±7 | ±7 | ±7 |  |
| 同意取得 | ● |  |  | |  |  |  |  |  |  |  |
| 登録及び割付 | ● |  |  | |  |  |  |  |  |  |  |
| 研究対象者の背景 | ● |  |  | |  |  |  |  |  |  |  |
| 身長・体重 | ● |  |  | |  |  |  |  |  |  |  |
| 併存疾患 | ● |  |  | |  |  |  |  |  |  |  |
| 既往疾患 | ● |  |  | |  |  |  |  |  |  |  |
| 試験薬の服用開始 |  | ● |  | |  |  |  |  |  |  |  |
| 試験薬服用記録 |  |  |  | |  |  |  |  |  |  |  |
| オンライン面談 | ● |  | ● | |  |  |  |  |  |  |  |
| 重症化リスク因子の有無 | ● |  |  | |  |  |  |  |  |  |  |
| 併用薬の確認 | ● |  | ● | |  |  |  |  |  |  |  |
| 妊娠の確認 | ● |  |  | |  |  |  |  |  |  |  |
| 罹患後症状 |  |  |  | | ● | ● | ● | ● | ● | ● | 〇 |
| QOL |  | ● |  | | ● | ● | ● | ● | ● | ● | 〇 |
| 労働生産性 |  | ● |  | | ● | ● | ● | ● | ● | ● | 〇 |
| 医療機関の受診や薬の処方について |  |  |  | | ● | ● | ● | ● | ● | ● | 〇 |
| COVID-19再罹患について |  |  |  | | ● | ● | ● | ● | ● | ● | 〇 |
| 有害事象^*3^ |  |  |  | |  |  |  |  |  |  |  |

●：必須項目、〇：可能な限り実施する項目

*1：発症から72時間以内に試験薬が服用開始できるように対応すること。

*2：試験薬服用又は研究が中止された場合、可能な限りオンライン面談を実施する。

*3：有害事象の収集は、Day 0より開始し、試験薬最終投与の2週間後まで実施する。

**目次**

[1. 略語・語句の定義 1](#_Toc183598251)

[1.1. 略語 1](#_Toc183598252)

[1.2. 語句の定義 1](#_Toc183598253)

[2. 研究の背景 2](#_Toc183598254)

[2.1. 対象疾患名 2](#_Toc183598255)

[2.2. 対象疾患の概念 2](#_Toc183598256)

[2.3. 対象疾患の疫学 2](#_Toc183598257)

[2.4. 標準治療 2](#_Toc183598258)

[2.5. COVID-19罹患後症状 3](#_Toc183598259)

[2.6. 本研究で検討する医薬品等 4](#_Toc183598260)

[2.7. 本研究を実施する意義 4](#_Toc183598261)

[3. 研究の目的及び評価項目 5](#_Toc183598262)

[3.1. 研究の目的 5](#_Toc183598263)

[3.2. 有効性主要評価項目 5](#_Toc183598264)

[3.3. 有効性副次評価項目 5](#_Toc183598265)

[3.4. 有効性探索評価項目 6](#_Toc183598266)

[3.5. 安全性評価項目 8](#_Toc183598267)

[4. 試験デザイン 8](#_Toc183598268)

[4.1. 試験デザイン 8](#_Toc183598269)

[4.2. 本研究の計画研究対象者数 9](#_Toc183598270)

[4.3. 本研究の予定実施期間 10](#_Toc183598271)

[5. 研究対象者の選定 11](#_Toc183598272)

[5.1. 選択基準 11](#_Toc183598273)

[5.2. 除外基準 11](#_Toc183598274)

[6. 研究の対象となる医薬品等 14](#_Toc183598275)

[6.1. 研究の対象となる医薬品等の概要 14](#_Toc183598276)

[6.1.1. 被験薬 14](#_Toc183598277)

[6.1.1. 対照薬 14](#_Toc183598278)

[6.2. 研究の対象となる医薬品等の品質の確保 14](#_Toc183598279)

[7. 研究対象者への医薬品等の適用方法（プロトコル治療） 16](#_Toc183598280)

[7.1. 研究対象の医薬品等の適用方法 16](#_Toc183598281)

[7.1.1. エンシトレルビル群 16](#_Toc183598282)

[7.1.2. プラセボ群 16](#_Toc183598283)

[7.1.3. 研究対象の医薬品等の適用方法の設定根拠 16](#_Toc183598284)

[7.2. 併用薬・併用療法 16](#_Toc183598285)

[7.3. 併用禁止薬 16](#_Toc183598286)

[8. 時点ごとの観察、検査及び評価 18](#_Toc183598287)

[8.1. 観察・検査・評価スケジュール 18](#_Toc183598288)

[8.1.1. 登録時（Visit 1） 18](#_Toc183598289)

[8.1.2. 試験薬服用開始日 18](#_Toc183598290)

[8.1.3. 1週後（Visit 2） 18](#_Toc183598291)

[8.1.4. 1ヵ月後（Visit 3）、2ヵ月後（Visit 4）、3ヵ月後（Visit 5）、4ヵ月後（Visit 6）、5ヵ月後（Visit 7）、6ヵ月後（Visit 8） 18](#_Toc183598292)

[8.1.5. 追跡期間中止時 18](#_Toc183598293)

[8.2. スタディーカレンダー 19](#_Toc183598294)

[9. 観察・検査及び評価の手順 20](#_Toc183598295)

[9.1. 本研究で使用するDecentralized Clinical Trials支援システム 20](#_Toc183598296)

[9.2. 研究対象者の募集 20](#_Toc183598297)

[9.3. 研究対象候補者登録 20](#_Toc183598298)

[9.4. 同意取得 20](#_Toc183598299)

[9.5. 登録、割付及び試験薬の送付 20](#_Toc183598300)

[9.5.1. 登録、割付及び試験薬の送付の手順 20](#_Toc183598301)

[9.5.2. 割付手順の作成及び保管 21](#_Toc183598302)

[9.5.3. 緊急キーオープン 21](#_Toc183598303)

[9.6. 試験薬服用記録 21](#_Toc183598304)

[9.7. 研究対象者の背景 21](#_Toc183598305)

[9.8. 身長・体重 22](#_Toc183598306)

[9.9. 併存疾患 22](#_Toc183598307)

[9.10. 既往疾患 22](#_Toc183598308)

[9.11. 重症化リスク因子の有無 22](#_Toc183598309)

[9.12. 併用薬の確認 22](#_Toc183598310)

[9.13. 妊娠の有無 22](#_Toc183598311)

[9.14. 罹患後症状 22](#_Toc183598312)

[9.15. QOL 22](#_Toc183598313)

[9.16. 労働生産性 22](#_Toc183598314)

[9.17. 医療機関の受診や薬の処方について 22](#_Toc183598315)

[9.18. COVID-19再罹患について 23](#_Toc183598316)

[9.19. 研究対象者ごとの中止及び終了の基準 23](#_Toc183598317)

[9.19.1. 研究対象者ごとの中止 23](#_Toc183598318)

[9.19.2. 研究対象者ごとの中止の手順 23](#_Toc183598319)

[9.19.3. 研究対象者ごとの終了 24](#_Toc183598320)

[10. 有害事象 25](#_Toc183598321)

[10.1. 有害事象の定義 25](#_Toc183598322)

[10.2. 有害事象の収集期間 25](#_Toc183598323)

[10.3. 自覚症状の確認 25](#_Toc183598324)

[10.4. 有害事象の評価 25](#_Toc183598325)

[10.4.1. 有害事象名 25](#_Toc183598326)

[10.4.2. 発現日 25](#_Toc183598327)

[10.4.3. 重症度 26](#_Toc183598328)

[10.4.4. 重篤性 26](#_Toc183598329)

[10.4.5. 研究との因果関係 26](#_Toc183598330)

[10.4.6. 試験薬との因果関係 27](#_Toc183598331)

[10.4.7. 転帰日 27](#_Toc183598332)

[10.4.8. 転帰 27](#_Toc183598333)

[10.5. 有害事象が発現した場合の措置 28](#_Toc183598334)

[10.5.1. 研究対象者への処置 28](#_Toc183598335)

[10.5.2. 有害事象の追跡調査 28](#_Toc183598336)

[10.5.3. 資金提供企業への報告 28](#_Toc183598337)

[10.6. 本研究で予想される有害事象 28](#_Toc183598338)

[11. 疾病等 30](#_Toc183598339)

[11.1. 疾病等の定義 30](#_Toc183598340)

[11.2. 重篤な疾病等の認定臨床研究審査委員会への報告手順 30](#_Toc183598341)

[11.3. 重篤な疾病等の厚生労働大臣への報告手順 31](#_Toc183598342)

[12. データマネジメント 32](#_Toc183598343)

[12.1. データマネジメント計画 32](#_Toc183598344)

[12.2. 症例報告書 32](#_Toc183598345)

[13. 統計的事項 33](#_Toc183598346)

[13.1. 解析集団の定義 33](#_Toc183598347)

[13.2. データの取り扱い 33](#_Toc183598348)

[13.2.1. 規定された許容範囲外の測定値の取扱い 33](#_Toc183598349)

[13.2.2. 欠測値の取扱い 33](#_Toc183598350)

[13.3. 解析方法 33](#_Toc183598351)

[13.3.1. 研究対象者の背景 33](#_Toc183598352)

[13.3.2. 試験薬服用の状況 33](#_Toc183598353)

[13.3.3. 併用薬 34](#_Toc183598354)

[13.3.4. 有効性主要評価項目 34](#_Toc183598355)

[13.3.5. 有効性副次評価項目 34](#_Toc183598356)

[13.3.6. 有効性探索評価項目 37](#_Toc183598357)

[13.3.7. 有効性評価項目に対するサブグループ解析 40](#_Toc183598358)

[13.3.8. 安全性評価項目 40](#_Toc183598359)

[13.4. 中間解析及び早期中止に関する基準 40](#_Toc183598360)

[13.5. 統計解析計画の変更 40](#_Toc183598361)

[14. 品質管理及び品質保証 41](#_Toc183598362)

[14.1. 品質管理方針 41](#_Toc183598363)

[14.2. 品質目標 41](#_Toc183598364)

[14.3. モニタリング 41](#_Toc183598365)

[14.4. 監査 41](#_Toc183598366)

[14.5. 規制当局等による調査への対応 41](#_Toc183598367)

[14.6. 不適合 42](#_Toc183598368)

[14.6.1. 不適合の定義 42](#_Toc183598369)

[14.6.2. 重大な不適合 42](#_Toc183598370)

[14.6.3. 不適合の管理手順 42](#_Toc183598371)

[15. 倫理的配慮 43](#_Toc183598372)

[15.1. 遵守すべき諸規則 43](#_Toc183598373)

[15.2. 認定臨床研究審査委員会及び実施医療機関の管理者の承認 43](#_Toc183598374)

[15.3. 本研究における研究対象者の費用負担 43](#_Toc183598375)

[15.4. 同意説明文書及び研究対象者の同意 43](#_Toc183598376)

[15.4.1. 同意取得手順 43](#_Toc183598377)

[15.4.2. 同意説明文書に記載すべき事項 44](#_Toc183598378)

[15.5. 研究対象者からの相談窓口 45](#_Toc183598379)

[15.6. 研究対象者の予想される利益及び不利益 45](#_Toc183598380)

[15.6.1. 予想される利益 45](#_Toc183598381)

[15.6.2. 予想される不利益 45](#_Toc183598382)

[15.6.3. 利益と不利益の総合評価及び不利益を最小化する対策 45](#_Toc183598383)

[15.7. 研究対象者の秘密保持（個人情報の保護） 45](#_Toc183598384)

[16. 健康被害に対する補償 47](#_Toc183598385)

[17. 臨床研究全体の中止又は終了 48](#_Toc183598386)

[17.1. 中止の基準 48](#_Toc183598387)

[17.2. 中止の手順 48](#_Toc183598388)

[17.3. 終了の基準 48](#_Toc183598389)

[18. 研究の情報公開及び結果公表 49](#_Toc183598390)

[18.1. 研究の登録 49](#_Toc183598391)

[18.2. 研究結果の公表 49](#_Toc183598392)

[18.2.1. 主要評価項目報告書 49](#_Toc183598393)

[18.2.2. 総括報告書 49](#_Toc183598394)

[18.2.3. 学会等の公表 49](#_Toc183598395)

[19. 変更管理 51](#_Toc183598396)

[19.1. 認定臨床研究審査委員会で承認された書類の変更 51](#_Toc183598397)

[19.2. 実施計画の変更 51](#_Toc183598398)

[19.3. 実施計画の軽微な変更 51](#_Toc183598399)

[20. 利益相反 52](#_Toc183598400)

[20.1. 本研究に関する資金源 52](#_Toc183598401)

[20.2. 利益相反管理 52](#_Toc183598402)

[21. 認定臨床研究審査委員会、厚生労働大臣に対する定期報告 53](#_Toc183598403)

[21.1. 認定臨床研究審査委員会に対する定期報告 53](#_Toc183598404)

[21.1.1. 定期報告における報告事項 53](#_Toc183598405)

[21.1.2. 定期報告の時期 53](#_Toc183598406)

[21.2. 厚生労働大臣に対する定期報告 53](#_Toc183598407)

[21.2.1. 定期報告における報告事項 53](#_Toc183598408)

[21.2.2. 定期報告の時期 53](#_Toc183598409)

[22. 資料及び記録等の保管並びに廃棄方法 54](#_Toc183598410)

[22.1. 原資料の保管 54](#_Toc183598411)

[22.2. 法で定める記録文書の保管 54](#_Toc183598412)

[22.3. 情報の二次利用について 55](#_Toc183598413)

[22.4. 廃棄の手順及び方法 55](#_Toc183598414)

[23. 研究結果の帰属 56](#_Toc183598415)

[24. 本研究で設置する委員会等 57](#_Toc183598416)

[24.1. 独立データモニタリング委員会 57](#_Toc183598417)

[25. 引用文献 62](#_Toc183598431)

1. 略語・語句の定義
   1. 略語

| 略語 | 完全型 | 日本語表記 |
| --- | --- | --- |
| COVID-19 | Coronavirus Disease 2019 | 新型コロナウイルス感染症 |
| SARS-CoV-2 | Severe acute respiratory syndrome coronavirus 2 | 新型コロナウイルス |

- 1. 語句の定義

1. 研究責任医師

臨床研究法に規定する臨床研究を実施する者をいい、実施医療機関において臨床研究に係る業務を統括する医師をいう。

1. 研究代表医師

多施設共同研究を実施する場合に、複数の実施医療機関の研究責任医師を代表する研究責任医師をいう。

1. 研究分担医師

実施医療機関において、研究責任医師の指導の下に臨床研究に係る業務を分担する医師をいう。

1. モニタリング

臨床研究に対する信頼性の確保及び研究対象者の保護の観点から臨床研究が適正に行われていることを確保するため、本研究の進捗状況並びに本研究が臨床研究法、同法施行規則及び研究計画書に従って行われているかどうかについて、研究代表医師が特定の者を指定して行わせる調査をいう。

1. 監査

臨床研究に対する信頼性の確保及び研究対象者の保護の観点から臨床研究により収集された資料の信頼性を確保するため、本研究が臨床研究法、同法施行規則及び研究計画書に従って行われたかどうかについて、研究代表医師が特定の者を指定して行わせる調査をいう。

1. 研究協力者

実施医療機関において、研究責任医師又は研究分担医師の指導の下にこれらの者の研究に係る業務に協力する薬剤師、看護師その他の医療関係者をいう。

1. 研究対象者紹介医療機関

研究対象者の候補となる患者を実施医療機関に紹介する医療機関

1. 研究の背景
   1. 対象疾患名

新型コロナウイルス感染症（COVID-19）

- 1. 対象疾患の概念

COVID-19は新型コロナウイルス（SARS-CoV-2）による呼吸器感染症である。接触感染・飛沫感染・エアロゾル感染の3つの感染経路によって伝播し、SARS-CoV-2がヒト細胞のACE2受容体に結合し感染が成立する。COVID-19診療の手引き 10.0版には、重症度分類として、以下のように軽症、中等症I、中等症II、重症の分類が定められている。


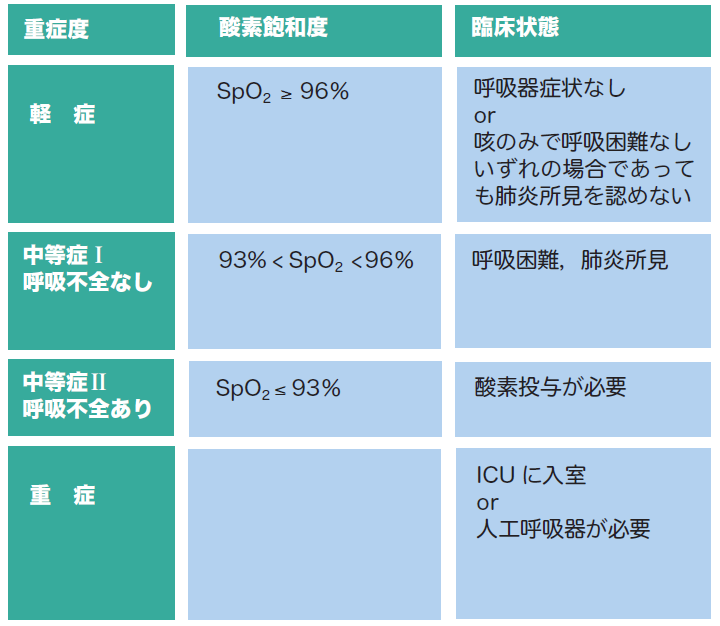


- 1. 対象疾患の疫学

2023年5月9日時点での日本国内のCOVID-19感染者数は累計33,803,572 人である（厚生労働省発表）。流行初期は小児の感染者は少ないことが特徴であったが、変異株が出現して以降は小児の感染も一般的となっている。女性よりも男性、若年者よりも高齢者の方が重症化しやすいが、罹患リスクには差はないと考えられている。

- 1. 標準治療

COVID-19に対する治療薬として、本邦では2023年8月22日時点で以下の薬剤が承認されている。

| 抗ウイルス薬 | レムデシビル（ベクルリー）、モルヌピラビル（ラゲブリオ）、ニルマトレルビル/リトナビル（パキロビッド）、エンシトレルビル フマル酸（ゾコーバ） |
| --- | --- |
| 中和抗体薬 | カシリビマブ/イムデミマブ（ロナプリーブ）、ソトロビマブ（ゼビュディ）チキサゲビマブ／シルガビマブ（エバシェルド） |
| 抗炎症薬・免疫調整薬 | デキサメタゾン（デキサート等）、バリシチニブ（オルミエント）、トシリズマブ（アクテムラ） |

一般名（商品名）

COVID-19診療の手引き 10.0版では、軽症、中等症Iには抗ウイルス薬や中和抗体薬が、中等症II以上ではデキサメタゾン、トシリズマブ、バリシチニブの使用が推奨されている。

本研究の対象である軽症患者に対する通常診療としては、対症療法が中心となる。高齢者や基礎疾患のある重症化リスクの高い患者では、ニルマトレルビル/リトナビル、レムデシビル、モルヌピラビルなどが使用されることがある。2023年12月時点では、エンシトレルビル フマル酸の投与により重症化を抑制できるというエビデンスはないが、重症化リスクのない軽症患者の有症状期間を約1日短縮させる効果があるため、重症化リスクの低い軽症患者に用いられる。

- 1. COVID-19罹患後症状

COVID-19罹患後症状とは、感染性は消失したにもかかわらず、他に明らかな原因がなく、急性期から持続する症状、あるいは経過の途中から新たに、又は再び生じて持続する症状全般を言う。代表的な症状は、けん怠感・疲労感、関節痛、筋肉痛、咳、喀痰、息切れ、胸痛、脱毛、記憶障害、集中力低下、頭痛、抑うつ、嗅覚障害、味覚障害、動悸、下痢、腹痛、睡眠障害、筋力低下である。

罹患後症状の病態機序は不明な点が多く、ウイルスに感染した組織（特に肺）への直接的な障害、微量なウイルスによる持続感染、ウイルス感染後の免疫調整不全による炎症の進行、ウイルスによる血液凝固能亢進と血栓症による血管損傷・虚血、ウイルス感染によるレニン・アンジオテンシン系の調整不全等があげられている。単一の病態ではなく、これらのいくつかが複合的に絡み合ったものが罹患後症状として現れている症例もあると考えられている。

日本で実施された、COVID-19と診断され入院歴のある患者1,066例を対象とした追跡調査では、診断12ヵ月後でも罹患者全体の30%程度に1つ以上の罹患後症状が認められた [1]。中等症以上の患者1,003例を対象とした研究では、筋力低下やけん怠感等の症状は時間とともに頻度が低下したが、12ヵ月後においていずれも約5～10%で認められ、何らかの罹患後症状は13.6%に残存していた [2]。また、大阪大学と豊中市が共同で実施した罹患後症状に関する観察研究（中等症II又は重症患者は約5%）では、アンケートの回答が得られた4,047例中47.7%が何らかの罹患後症状を経験しており、発症30日後で5.2%、60日後で3.7%の者がけん怠感、咳、脱毛等の罹患後症状を訴えていた [3]。

2023年2月に日本赤十字社で献血した16歳〜69歳の13,121名を対象に実施した抗ヌクレオカプシド抗体陽性率の調査では、4割以上の人がSARS-CoV-2に感染している可能性が示されており [4]、誰がいつ感染してもおかしくない状況である一方で、罹患後症状の予防法や治療法は確立されていないことが大きな課題となっている。

COVID-19に対する標準治療となっている薬剤のうち、ニルマトレルビル/リトナビルを発症から5日以内に内服した患者では、内服しなかった患者と比較して罹患後症状のリスクが26％減ったという医療保険データを用いた研究結果が発表され [5]、急性期における抗ウイルス薬の投与が罹患後症状のリスク低減につながる可能性が示唆されている。しかし、ニルマトレルビル/リトナビルは重症化リスクのある患者のみに適応となっているために投与対象が限られる。

- 1. 本研究で検討する医薬品等

エンシトレルビル フマル酸は、塩野義製薬株式会社によって創製されたSARS-CoV-2による感染症治療薬である。本剤は、SARS-CoV-2遺伝子にコードされるポリタンパク質のプロセシング及びウイルス複製に必須である3C-likeプロテアーゼを阻害することで、SARS-CoV-2に対する抗ウイルス効果を発揮する。

12 歳以上70 歳未満のCOVID-19患者を対象に、1日目はエンシトレルビル375 mgを、2日目～5日目は125 mgを1 日1 回経口投与するプラセボ対照無作為化二重盲検並行群間試験（国際共同第2/3相試験［T1221試験］第3相パート）では、参加した1,215 例（日本人662 例）のうち、COVID-19の症状発現から無作為化割付までの時間が72時間未満であった690 例において、5 症状（けん怠感又は疲労感、熱っぽさ又は発熱、鼻水又は鼻づまり、喉の痛み、咳）が快復するまでの時間の中央値はエンシトレルビル群で167.9 時間、プラセボ群で192.2 時間であった（p=0.0407）。副作用発現頻度は24.5％（148/604 例）であり、主な副作用は高比重リポ蛋白の減少であった（18.4％）。

2022年11月に緊急承認医薬品として承認を取得した。

- 1. 本研究を実施する意義

上記の臨床試験での罹患後症状についての探索的評価（COVID-19に特徴的な14症状のベースライン時のスコアが中央値以上の患者集団における投与開始後169日目までのサブ解析）では、14症状が発症した割合はプラセボ群で27%、エンシトレルビル群が15％（相対リスク低下率45%）、集中力低下や不眠等の神経系の4症状が発症した割合はプラセボ群で43%、エンシトレルビル群で29%（相対リスク低下率33%）であることが示された [6]。しかし、主要評価項目ではなく探索的評価であるために、より検証的な研究結果が望まれる。

エンシトレルビル フマル酸は重症化リスクのない患者にも処方可能な抗ウイルス薬であり、罹患後症状の発症抑制にも有効であることがより信頼性の高い研究デザインによって示されれば、罹患後症状という社会的課題の解決に近づくことができる可能性がある。

1. 研究の目的及び評価項目
   1. 研究の目的

軽症COVID-19患者を対象にして、エンシトレルビル フマル酸を5日間投与した時の罹患後症状におけるエンシトレルビルのプラセボに対する優越性を検証する。また、エンシトレルビルの安全性を評価する。

- 1. 有効性主要評価項目

「治療開始から1ヵ月及び3ヵ月の2時点両方で、けん怠感（疲労感）、息切れ又は呼吸困難感、嗅覚の異常、味覚の異常のいずれかの症状がある」又は「3ヵ月時点で集中力・思考力の低下、課題解決力の低下、物忘れ（短期又は長期）のいずれかの症状がある」研究対象者の割合

【主要評価項目の設定根拠】

罹患後症状のうち発現頻度が高い症状に限定して評価することとした。また、WHOにおけるCOVID-19罹患後症状の定義が「少なくとも2ヵ月以上持続し、また他の疾患による症状として説明がつかないものである。通常はCOVID-19の発症から3ヵ月経った時点にもみられる」となっていることから、治療開始から3ヵ月後に評価することとし、他の疾患による症状としても発生し得るCOVID-19急性期症状については治療開始から1ヵ月時点と3ヵ月時点の両方で症状が存在することを条件とした。

- 1. 有効性副次評価項目

1. 「治療開始から1ヵ月及び3ヵ月の2時点両方で、けん怠感（疲労感）、息切れ又は呼吸困難感、嗅覚の異常、味覚の異常のいずれかの症状がある」又は「3ヵ月時点で集中力・思考力の低下、課題解決力の低下、物忘れ（短期又は長期）のいずれかの症状がある」研究対象者の割合（COVID-19と関連がある、又は関連不明）
2. 「治療開始から1ヵ月及び3ヵ月及び6ヵ月の3時点全てで、けん怠感（疲労感）、息切れ又は呼吸困難感、嗅覚の異常、味覚の異常のいずれかの症状がある」又は「6ヵ月時点で集中力・思考力の低下、課題解決力の低下、物忘れ（短期又は長期）のいずれかの症状がある」研究対象者の割合
3. 「治療開始から1ヵ月及び3ヵ月及び6ヵ月の3時点全てで、けん怠感（疲労感）、息切れ又は呼吸困難感、嗅覚の異常、味覚の異常のいずれかの症状がある」又は「6ヵ月時点で集中力・思考力の低下、課題解決力の低下、物忘れ（短期又は長期）のいずれかの症状がある」研究対象者の割合（COVID-19と関連がある、又は関連不明）
4. 治療開始から1ヵ月及び3ヵ月の2時点両方で、けん怠感（疲労感）、息切れ又は呼吸困難感、嗅覚の異常、味覚の異常のいずれかの症状がある研究対象者の割合
5. 治療開始から1ヵ月及び3ヵ月の2時点両方で、けん怠感（疲労感）、息切れ又は呼吸困難感、嗅覚の異常、味覚の異常のいずれかの症状がある研究対象者の割合（COVID-19と関連がある、又は関連不明）
6. 治療開始から1ヵ月及び3ヵ月の2時点両方で、けん怠感（疲労感）、息切れ又は呼吸困難感、嗅覚の異常、味覚の異常の各症状がある研究対象者の割合
7. 治療開始から1ヵ月及び3ヵ月の2時点両方で、けん怠感（疲労感）、息切れ又は呼吸困難感、嗅覚の異常、味覚の異常の各症状がある研究対象者の割合（COVID-19と関連がある、又は関連不明）
8. 治療開始から3ヵ月時点で集中力・思考力の低下、課題解決力の低下、物忘れ（短期又は長期）のいずれかの症状がある研究対象者の割合
9. 治療開始から3ヵ月時点で集中力・思考力の低下、課題解決力の低下、物忘れ（短期又は長期）のいずれかの症状がある研究対象者の割合（COVID-19と関連がある、又は関連不明）
10. 治療開始から3ヵ月時点で集中力・思考力の低下、課題解決力の低下、物忘れ（短期又は長期）の各症状がある研究対象者の割合
11. 治療開始から3ヵ月時点で集中力・思考力の低下、課題解決力の低下、物忘れ（短期又は長期）の各症状がある研究対象者の割合（COVID-19と関連がある、又は関連不明）
12. 治療開始から3ヵ月時点でCOVID-19 罹患前同様の通常の健康状態に戻っておらず、COVID-19の14症状（けん怠感（疲労感）、体の痛み又は筋肉痛、頭痛、悪寒、熱っぽさ、鼻水もしくは鼻づまり、喉の痛み、咳、息切れ又は呼吸困難、吐き気、嘔吐、下痢、嗅覚の異常、味覚の異常）のいずれかの症状がある研究対象者の割合
13. 治療開始から3ヵ月時点でCOVID-19 罹患前同様の通常の健康状態に戻っておらず、神経学的4症状（集中力・思考力の低下、課題解決力の低下、物忘れ（短期又は長期）、不眠）のいずれかの症状がある研究対象者の割合
14. 治療開始から3ヵ月時点でCOVID-19 罹患前同様の通常の健康状態に戻っておらず、けん怠感（疲労感）、体の痛み又は筋肉痛、頭痛、悪寒、熱っぽさ、鼻水もしくは鼻づまり、喉の痛み、咳、息切れ又は呼吸困難、吐き気、嘔吐、下痢、嗅覚の異常、味覚の異常、筋力低下、集中力・思考力の低下、課題解決力の低下、物忘れ（短期又は長期）、不眠、脱毛、動悸又は心拍数の増加、関節痛、食欲不振、めまい又は平衡感覚の異常、胸痛、皮膚の発疹のいずれかの症状がある研究対象者の割合

【副次評価項目の設定根拠】

1. 主要評価項目の症状のうち、研究対象者自身がCOVID-19と関連がある、又は関連不明と判断した罹患後症状に対するエンシトレルビル フマル酸の効果を評価するため。
2. 主要評価項目で設定した時点よりも長期における罹患後症状へのエンシトレルビル フマル酸の効果を評価するため。
3. （２）のうち、研究対象者自身がCOVID-19と関連がある、又は関連不明と判断した罹患後症状に対するエンシトレルビル フマル酸の効果を評価するため。
4. 急性期から持続している罹患後症状へのエンシトレルビル フマル酸の効果を評価するため。
5. （４）のうち、研究対象者自身がCOVID-19と関連がある、又は関連不明と判断した罹患後症状に対するエンシトレルビル フマル酸の効果を評価するため。
6. 急性期から持続している各罹患後症状へのエンシトレルビル フマル酸の効果を評価するため。
7. （６）のうち、研究対象者自身がCOVID-19と関連がある、又は関連不明と判断した罹患後症状に対するエンシトレルビル フマル酸の効果を評価するため。
8. 神経学的な罹患後症状へのエンシトレルビル フマル酸の効果を評価するため。
9. （８）のうち、研究対象者自身がCOVID-19と関連がある、又は関連不明と判断した罹患後症状に対するエンシトレルビル フマル酸の効果を評価するため。
10. 神経学的な各罹患後症状へのエンシトレルビル フマル酸の効果を評価するため
11. （１０）のうち、研究対象者自身がCOVID-19と関連がある、又は関連不明と判断した罹患後症状に対するエンシトレルビル フマル酸の効果を評価するため。
12. 通常状態に戻っていない患者における急性期症状と関連する罹患後症状へのエンシトレルビル フマル酸の効果を評価するため。
13. 通常状態に戻っていない患者における神経学的な罹患後症状へのエンシトレルビル フマル酸の効果を評価するため。
14. 通常状態に戻っていない患者における全般的な罹患後症状へのエンシトレルビル フマル酸の効果を評価するため。
    1. 有効性探索評価項目
15. 「治療開始から2ヵ月後及び3ヵ月の2時点連続で、けん怠感（疲労感）、息切れ又は呼吸困難感、嗅覚の異常、味覚の異常のいずれかの症状がある」又は「3ヵ月時点で集中力・思考力の低下、課題解決力の低下、物忘れ（短期又は長期）のいずれかの症状がある」研究対象者の割合
16. 「治療開始から2ヵ月後及び3ヵ月の2時点連続で、けん怠感（疲労感）、息切れ又は呼吸困難感、嗅覚の異常、味覚の異常のいずれかの症状がある」又は「3ヵ月時点で集中力・思考力の低下、課題解決力の低下、物忘れ（短期又は長期）のいずれかの症状がある」研究対象者の割合（COVID-19と関連がある、又は関連不明）
17. 治療開始から2ヵ月及び3ヵ月の2時点連続で、けん怠感（疲労感）、息切れ又は呼吸困難感、嗅覚の異常、味覚の異常のいずれかの症状がある研究対象者の割合
18. 治療開始から2ヵ月及び3ヵ月の2時点連続で、けん怠感（疲労感）、息切れ又は呼吸困難感、嗅覚の異常、味覚の異常のいずれかの症状がある研究対象者の割合（COVID-19と関連がある、又は関連不明）
19. 治療開始から2ヵ月及び3ヵ月の2時点連続で、けん怠感（疲労感）、息切れ又は呼吸困難感、嗅覚の異常、味覚の異常の各症状がある研究対象者の割合
20. 治療開始から2ヵ月及び3ヵ月の2時点連続で、けん怠感（疲労感）、息切れ又は呼吸困難感、嗅覚の異常、味覚の異常の各症状がある研究対象者の割合（COVID-19と関連がある、又は関連不明）
21. 「治療開始から1ヵ月及び3ヵ月の2時点両方でCOVID-19の14症状（けん怠感（疲労感）、体の痛み又は筋肉痛、頭痛、悪寒、熱っぽさ、鼻水もしくは鼻づまり、喉の痛み、咳、息切れ又は呼吸困難、吐き気、嘔吐、下痢、嗅覚の異常、味覚の異常）のいずれかがある」又は「3ヵ月時点で筋力低下、集中力・思考力の低下、課題解決力の低下、物忘れ（短期又は長期）、不眠、脱毛、動悸又は心拍数の増加、関節痛、食欲不振、めまい又は平衡感覚の異常、胸痛、皮膚の発疹のいずれかの症状がある」研究対象者の割合
22. 「治療開始から1ヵ月及び3ヵ月の2時点両方でCOVID-19の14症状（けん怠感（疲労感）、体の痛み又は筋肉痛、頭痛、悪寒、熱っぽさ、鼻水もしくは鼻づまり、喉の痛み、咳、息切れ又は呼吸困難、吐き気、嘔吐、下痢、嗅覚の異常、味覚の異常）のいずれかがある」又は「3ヵ月時点で筋力低下、集中力・思考力の低下、課題解決力の低下、物忘れ（短期又は長期）、不眠、脱毛、動悸又は心拍数の増加、関節痛、食欲不振、めまい又は平衡感覚の異常、胸痛、皮膚の発疹のいずれかの症状がある」研究対象者の割合（COVID-19と関連がある、又は関連不明）
23. 治療開始から1ヵ月及び3ヵ月の2時点両方で COVID-19の14症状（けん怠感（疲労感）、体の痛み又は筋肉痛、頭痛、悪寒、熱っぽさ、鼻水もしくは鼻づまり、喉の痛み、咳、息切れ又は呼吸困難、吐き気、嘔吐、下痢、嗅覚の異常、味覚の異常）のいずれかの症状がある研究対象者の割合
24. 治療開始から1ヵ月及び3ヵ月の2時点両方で COVID-19の14症状（けん怠感（疲労感）、体の痛み又は筋肉痛、頭痛、悪寒、熱っぽさ、鼻水もしくは鼻づまり、喉の痛み、咳、息切れ又は呼吸困難、吐き気、嘔吐、下痢、嗅覚の異常、味覚の異常）のいずれかの症状がある研究対象者の割合（COVID-19と関連がある、又は関連不明）
25. 治療開始から1ヵ月及び3ヵ月の2時点両方で、 COVID-19の14症状（けん怠感（疲労感）、体の痛み又は筋肉痛、頭痛、悪寒、熱っぽさ、鼻水もしくは鼻づまり、喉の痛み、咳、息切れ又は呼吸困難、吐き気、嘔吐、下痢、嗅覚の異常、味覚の異常）の各症状がある研究対象者の割合
26. 治療開始から1ヵ月及び3ヵ月の2時点両方で、 COVID-19の14症状（けん怠感（疲労感）、体の痛み又は筋肉痛、頭痛、悪寒、熱っぽさ、鼻水もしくは鼻づまり、喉の痛み、咳、息切れ又は呼吸困難、吐き気、嘔吐、下痢、嗅覚の異常、味覚の異常）の各症状がある研究対象者の割合（COVID-19と関連がある、又は関連不明）
27. 治療開始から3ヵ月時点で筋力低下、集中力・思考力の低下、課題解決力の低下、物忘れ（短期又は長期）、不眠、脱毛、動悸又は心拍数の増加、関節痛、食欲不振、めまい又は平衡感覚の異常、胸痛、皮膚の発疹のいずれかの症状がある研究対象者の割合
28. 治療開始から3ヵ月時点で筋力低下、集中力・思考力の低下、課題解決力の低下、物忘れ（短期又は長期）、不眠、脱毛、動悸又は心拍数の増加、関節痛、食欲不振、めまい又は平衡感覚の異常、胸痛、皮膚の発疹のいずれかの症状がある研究対象者の割合（COVID-19と関連がある、又は関連不明）
29. 治療開始から3ヵ月時点で筋力低下、集中力・思考力の低下、課題解決力の低下、物忘れ（短期又は長期）、不眠、脱毛、動悸又は心拍数の増加、関節痛、食欲不振、めまい又は平衡感覚の異常、胸痛、皮膚の発疹の各症状がある研究対象者の割合
30. 治療開始から3ヵ月時点で筋力低下、集中力・思考力の低下、課題解決力の低下、物忘れ（短期又は長期）、不眠、脱毛、動悸又は心拍数の増加、関節痛、食欲不振、めまい又は平衡感覚の異常、胸痛、皮膚の発疹の各症状がある研究対象者の割合（COVID-19と関連がある、又は関連不明）
31. 治療開始から3ヵ月時点でCOVID-19 罹患前同様の通常の健康状態に戻っていない研究対象者の割合
32. 治療開始から3ヵ月後及び6ヵ月後のそれぞれの時点における、 QOLのベースラインからの変化量
33. 治療開始から3ヵ月後及び6ヵ月後のそれぞれの時点における、労働生産性のベースラインからの変化量

【探索評価項目の設定根拠】

1. 症状継続期間を短くした設定した場合の罹患後症状へのエンシトレルビル フマル酸の効果を探索するため。
2. （１）のうち、研究対象者自身がCOVID-19と関連がある、又は関連不明と判断した罹患後症状に対するエンシトレルビル フマル酸の効果を評価するため。
3. 症状継続期間を短くした設定した場合の急性期から持続している罹患後症状へのエンシトレルビル フマル酸の効果を探索するため。
4. （３）のうち、研究対象者自身がCOVID-19と関連がある、又は関連不明と判断した罹患後症状に対するエンシトレルビル フマル酸の効果を評価するため。
5. 症状継続期間を短くした設定した場合の急性期から持続している各罹患後症状へのエンシトレルビル フマル酸の効果を探索するため。
6. （５）のうち、研究対象者自身がCOVID-19と関連がある、又は関連不明と判断した罹患後症状に対するエンシトレルビル フマル酸の効果を評価するため。
7. 広範な罹患後症状へのエンシトレルビル フマル酸の効果を探索するため。
8. （７）のうち、研究対象者自身がCOVID-19と関連がある、又は関連不明と判断した罹患後症状に対するエンシトレルビル フマル酸の効果を評価するため。
9. 広範な急性期症状と関連する罹患後症状へのエンシトレルビル フマル酸の効果を探索するため。
10. （９）のうち、研究対象者自身がCOVID-19と関連がある、又は関連不明と判断した罹患後症状に対するエンシトレルビル フマル酸の効果を評価するため。
11. 広範な急性期症状と関連する各罹患後症状へのエンシトレルビル フマル酸の効果を探索するため。
12. （11）のうち、研究対象者自身がCOVID-19と関連がある、又は関連不明と判断した罹患後症状に対するエンシトレルビル フマル酸の効果を評価するため。
13. その他の広範な罹患後症状へのエンシトレルビル フマル酸の効果を探索するため。
14. （13）のうち、研究対象者自身がCOVID-19と関連がある、又は関連不明と判断した罹患後症状に対するエンシトレルビル フマル酸の効果を評価するため。
15. その他の広範な各罹患後症状へのエンシトレルビル フマル酸の効果を探索するため。
16. （15）のうち、研究対象者自身がCOVID-19と関連がある、又は関連不明と判断した罹患後症状に対するエンシトレルビル フマル酸の効果を評価するため。
17. 研究計画書で評価する症状以外の罹患後症状が原因で通常状態に戻っていない患者におけるエンシトレルビル フマル酸の効果を評価するため。
18. エンシトレルビル フマル酸のQOLに対する効果を評価するため。
19. エンシトレルビル フマル酸の労働生産性スコアに対する効果を評価するため。
    1. 安全性評価項目

（１）有害事象の発現例数及び件数とその割合

（２）重篤な有害事象の発現例数及び件数とその割合

【安全性評価項目の設定根拠】

（１）（２）エンシトレルビル フマル酸の安全性を評価するため。

1. 試験デザイン
   1. 試験デザイン

下記の２群による、多施設、無作為割付、二重盲検、並行群間比較試験。

1. エンシトレルビル群
2. プラセボ群

割付にあたり、以下を割付因子とした層別置換ブロック法を適用する。

・COVID-19ワクチン接種歴の有無

・同意取得時のCOVID-19症状（14症状）の重症度（9点未満、9点以上）

6ヵ月後

↓

服用5日目

↓

服用開始

↓

罹患後症状の確認

割付

エンシトレルビル フマル酸群

罹患後症状の確認

プラセボ

【試験デザインの設定根拠】

群間の患者背景の偏りを最小限とし比較可能性を高めるために、無作為割付を行う。COVID-19ワクチン接種が罹患後症状のリスクを減少させる可能性を示唆する報告があること [6]、また、国際共同第2/3相試験［T1221試験］第3相パートでの探索的解析においてベースラインのCOVID-19症状（14症状）の重症度*が9点以上の群では9点未満より罹患後症状の発現が多いことが示されたことからこれらを割付因子とした。主要評価項目は研究対象者の自覚症状であり、割付群がわかることが評価に影響を与える可能性があるために盲検化を実施する。

＊けん怠感 [疲労感]、筋肉痛又は体の痛み、頭痛、悪寒/発汗、熱っぽさ又は発熱、鼻水又は鼻づまり、喉の痛み、咳、息切れ、吐き気、嘔吐、下痢を4段階(0：症状なし、1：軽度、2：中等度、3：重度）で点数化し、味覚異常、嗅覚異常を3段階(0：通常どおり、1：通常に比べて感じない、2：全く感じない）で点数化した場合の合計。

新型コロナウイルス感染症　診療の手引き（第10.0版）において、重症化リスクの低い患者では抗ウイルス薬を用いない治療も選択肢に含まれており、本研究の対象者（軽症患者）でのプラセボ投与は可能だと考える。罹患後症状に対してはいまだ確立した予防法や治療法がなく、対照とできる既存薬はない。以上よりプラセボを対照と設定することは倫理的に許容されると考える。

- 1. 本研究の計画研究対象者数

登録する研究対象者数として合計2,000例

エンシトレルビル群：1,000例

プラセボ群：1,000例

【計画研究対象者数の設定根拠】

エンシトレルビル フマル酸の国際共同第2/3相試験［T1221試験］第3相パートの日本人ITT集団において、COVID-19罹患後に「治療開始から1ヵ月及び3ヵ月の2時点両方で、けん怠感（疲労感）、息切れ又は呼吸困難感、嗅覚の異常、味覚の異常のいずれかの症状がある」又は「3ヵ月時点で集中力・思考力の低下、課題解決力の低下、物忘れ（短期又は長期）のいずれかの症状がある」研究対象者の割合はプラセボ群で22.4%、エンシトレルビル群で15.4%、相対リスクは68.9%であった。

また、研究計画時点直近に実施中であるエンシトレルビル フマル酸の国際共同第3相試験［SCORPIO-HR 試験］において、盲検下における2023年10月時点の実薬とプラセボの両群合わせた罹患後症状の発症率は12.4%であった。罹患後症状の発症率は流行株によって変化していることを考慮し、全体発症率としては本発症率を参照した。全体発症率を12.4%、相対リスクを70%と仮定すると、罹患後症状の発症率はプラセボ群で14.6%、エンシトレルビル群で10.2%と算出された。

帰無仮説をプラセボ群とエンシトレルビル群で罹患後症状の発症率が同じとし、対立仮説をプラセボ群とエンシトレルビル群で罹患後症状の発症率が同じではないとする。母比率の差の検定を行い必要症例数を算出した。

[中間解析を実施する場合]

中間解析実施時点での情報分数を50%とし、α消費関数に基づくO’Brien–Flemming 境界を用いた有効性中止基準を採用する想定の下で、非曝露群におけるアウトカムの割合を14.6%、相対リスクを保守的に70%、有意水準を両側5％、検出力を80%と設定した際に必要な症例数は1,818例と算出された。約1割が脱落することを想定し目標症例数は2,000例と設定した。

[中間解析を実施しない場合]

非曝露群におけるアウトカムの割合を14.6%、相対リスクを70%、有意水準を両側5％、検出力を80%と設定した際に必要な症例数は1,784例と算出された。約1割が脱落することを想定し目標症例数は2,000例と設定した。

- 1. 本研究の予定実施期間

1. 研究予定期間

jRCT公表日～2026年12月31日

1. 登録予定期間

jRCT公表日～2025年12月31日

1. 観察予定期間

jRCT公表日～2026年6月30日

1. 研究対象者の選定

選択基準をすべて満たし、除外基準に抵触しない患者を研究対象者とする。

- 1. 選択基準

1. 核酸増幅法（PCR 法、LAMP法等）、抗原定性検査又は抗原定量検査で研究対象者紹介医療機関においてCOVID-19と診断された者
2. 同意取得時に新型コロナウイルス感染症（COVID-19）診療の手引き（第10.0版）での重症度分類が軽症の者
3. 発症*から72時間以内に試験薬の服用開始が期待できる者
   *COVID-19の14症状(けん怠感 [疲労感]、筋肉痛又は体の痛み、頭痛、悪寒/発汗、熱っぽさ又は発熱、鼻水又は鼻づまり、喉の痛み、咳、息切れ [呼吸困難]、吐き気、嘔吐、下痢、味覚異常、嗅覚異常)のいずれか1項目以上を有したと患者が判断した時点
4. 研究対象者紹介医療機関での確定診断時の体温が37.0℃以上の者
5. 妊娠する可能性のある女性では、試験薬の服用期間中及び最終服用後2週間以内に性交渉を行う場合にパートナーと共に適切な避妊を行うことが可能な者
6. 同意取得時の年齢が18歳以上である者
7. 本研究への参加について文書による同意が本人から得られる者

【選択基準の設定根拠】

（１）～（３）エンシトレルビル フマル酸の有効性評価に適切な研究対象者を選定するため。

（４）国際共同第2/3相試験［T1221試験］第3相パートのサブグループ解析で、体温が37℃以上であった被験者での罹患後症状の発生頻度が高いことが示されたため。

（５）エンシトレルビル フマル酸は、動物実験で、ウサギの胎児に催奇形性が認められており、人での影響はわかっていないものの、妊娠中に服用することで、胎児奇形を起こす可能性があるため。最終服用後の避妊期間は、健康成人女性を対象とした試験におけるエンシトレルビル フマル酸の半減期の中央値（51.4時間）及び最大値（66.4時間）の5倍に相当する。

（６）（７）適切な同意が得られた成人を対象に研究を実施するため。

- 1. 除外基準

1. COVID-19の重症化リスクが高い者
2. 今回のCOVID-19を発症してから、又は同意取得日前15日以内にCOVID-19への効能効果を有する抗ウイルス薬（レムデシビル、モルヌピラビル、ニルマトレルビル／リトナビル、エンシトレルビル フマル酸等）、中和抗体薬（カシリビマブ／イムデビマブ、ソトロビマブ、チキサゲビマブ／シルガビマブ等）の投与を受けた者
3. 同意取得前2週間以内に抗IL-6抗体製剤（トシリズマブ等）、JAK阻害薬（バリシチニブ等。外用を除く）、内服、坐薬又は注射用のステロイド薬の投与を受けた者
4. 今回のCOVID-19を発症してからCOVID-19治療薬の臨床試験に参加したことがある者
5. スマートフォンを利用した研究責任医師又は研究分担医師とのオンライン面談が不可能な者
6. スマートフォンを利用した患者報告アウトカム収集システムへの入力が不可能な者
7. エンシトレルビル製剤の成分に対し過敏症の既往歴のある患者
8. エンシトレルビル フマル酸の併用禁忌となっている薬剤（7.3項参照）を投与中の者、又は試験薬の投与期間中及び最終投与後2週間以内に投与予定がある者
9. 腎機能又は肝機能障害のある患者で、コルヒチンを投与中の者
10. 妊娠又は妊娠している可能性のある女性（妊娠の可能性については、月経歴、及び最近の性行為を確認したうえで判断する）
11. 授乳中の女性
12. 重度の肝機能障害を有する者
13. 免疫不全患者及び透析中の者

「一般社団法人日本感染症学会　ワクチン委員会・COVID-19ワクチン・タスクフォース　COVID-19ワクチンに関する提言（第7版）」の表37を参考とする．

1. 他の感染症を併存している者
2. 他の介入研究に参加中の者又は本研究に参加したことのある者
3. 過去1年以内に医薬品の治験に参加したことがある者
4. その他の理由で、研究責任医師又は研究分担医師が研究対象者として不適当と判断した者

【除外基準の設定根拠】

（１）新型コロナウイルス感染症（COVID-19）診療の手引き（第10.0版）では、重症化リスクが高い患者に対してニルマトレルビル／リトナビル、レムデシビル又はモルヌピラビルの投与が推奨されているため。

（２）～（４）COVID-19治療薬の投与による有効性評価への影響を避けるために設定した。また、COVID-19への効能を持っていない抗IL-6抗体製剤、JAK阻害薬（外用を除く）、内服、坐薬又は注射用のステロイド薬も有効性評価に影響を及ぼす可能性があるため。

（５）研究責任医師又は研究分担医師が研究対象者とオンライン面談を行うため。

（６）患者報告アウトカム収集システムを使用するため。

（７）～（１０）エンシトレルビル フマル酸の投与禁忌であり、研究対象者の安全性を考慮したため。

（１１）エンシトレルビル フマル酸の投与が推奨されておらず、乳児の安全性を考慮したため。（１２）エンシトレルビル フマル酸の投与が推奨されておらず、研究対象者の安全性を考慮したため。

（１３）免疫能の低下によるCOVID-19の重症化リスクがあり、プラセボ投与時の安全性を考慮したため。

（１４）他の感染症の併存によって発熱等の症状がある場合、有効性評価が困難となるため。

（１５）本研究及び参加中の介入研究に対して予測不能な影響を与える可能性があるため。

（１６）エンシトレルビル フマル酸は、強いCYP3A阻害作用を有し、また、P-gp、BCRP、OATP1B1及びOATP1B3阻害作用を有し、治験薬との未知の相互作用がみられる可能性があるため。半減期が長い治験薬があることを想定して保守的に「過去1年以内」とした。

（１７）研究責任医師又は研究分担医師が上記以外の理由で本研究には不適当と判断した者を除外するため。

1. 研究の対象となる医薬品等
   1. 研究の対象となる医薬品等の概要

本研究では以下の試験薬（被験薬及び対照薬）を用いる。

| 試験薬の名称 | エンシトレルビル フマル酸 125mg/Placebo |
| --- | --- |
| 製造番号又は製造記号 | CF21014 |
| 外見 | 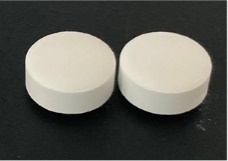  直径約9.0mm、厚さ約4.9mmの素錠  被験薬と対照薬の外見等は同じであり識別不能 |
| 保管の方法 | 室温保存 |

- - 1. 被験薬

| 医薬品医療機器等法における未承認、  適応外、承認内の別 | | □　未承認 | □　適応外 | ■　承認内 |
| --- | --- | --- | --- | --- |
| 一般名称（国内外で未承認の場合は開発コードを記載する） | | エンシトレルビル フマル酸 | | |
| 販売名（海外製品の場合は国名も記載する） | | ゾコーバ錠125 mg | | |
| 製造販売会社名 | | 塩野義製薬株式会社 | | |
| 承認番号 | | 30400AMX00205000 | | |
| 被験薬等提供者 | 名称 | 塩野義製薬株式会社 | | |
|  | 所在地 | 大阪市中央区道修町3-1-8 | | |

- - 1. 対照薬

| 医薬品医療機器等法における未承認、  適応外、承認内の別 | | ■　未承認 | □　適応外 | □　承認内 |
| --- | --- | --- | --- | --- |
| 一般名称（国内外で未承認の場合は開発コードを記載する） | | プラセボ | | |
| 被験薬等提供者 | 名称 | 塩野義製薬株式会社 | | |
|  | 所在地 | 大阪市中央区道修町3-1-8 | | |

- 1. 研究の対象となる医薬品等の品質の確保

研究代表医師は、以下に掲げる事項を実施する。

1　医薬品等の成分、分量、規格及び研究方法、性能並びに構造に関する事項、製造等を行う方法に関する事項、医薬品等の包装・表示に関する事項、臨床研究における使用方法その他必要な事項について記載した文書を作成又は入手し、保存する。

2　医薬品等の製造年月日、製造番号又は製造記号その他の当該医薬品等の製造に関して記録し、保存する。

3　医薬品等の入手に関して、その数量及び年月日を記録し、保存する。

4　研究対象者ごとに、医薬品等の使用に関して、その数量及び年月日を記録し、保存する。

5　医薬品等の処分に関して、その数量及び年月日を記録し、保存する。

6　臨床研究に用いる医薬品等の品質が不良である等の情報を得たときには、その検証を行い、臨床研究の停止等の講ずる措置について、認定臨床研究審査委員会に報告する。また、その記録を作成する。

7 臨床研究に用いる医薬品等の品質が不良である等の理由により、医薬品等の回収が必要と判断したときは、速やかに認定臨床研究審査委員会に報告するとともに、以下の業務を行う。

（ア）研究責任医師に対し、医薬品等の使用中止と回収の指示を速やかに行う。

（イ）回収の内容、原因究明の結果及び改善措置を記載した回収処理記録を作成し、保存する。

研究責任医師は、以下に掲げる事項を実施する。

1　研究代表医師から通知を受けた場合には、研究分担医師等に対し、医薬品等の使用中止と回収の指示を速やかに行う。

1. 研究対象者への医薬品等の適用方法（プロトコル治療）
   1. 研究対象の医薬品等の適用方法
      1. エンシトレルビル群

エンシトレルビルとして1日目は375mgを、2日目から5日目は125mgを1日1回経口投与する。

- - 1. プラセボ群

プラセボを1日目は3錠を、2日目から5日目は1錠を1日1回経口投与する。

- - 1. 研究対象の医薬品等の適用方法の設定根拠

エンシトレルビル フマル酸の承認用法・用量に従った。

- 1. 併用薬・併用療法

COVID-19の症状への対症療法（解熱鎮痛薬や鎮咳薬等）及び罹患後症状への対症療法の実施は可とする。

併存疾患に対しては、通常診療として併用薬投与や併用療法を行う。

- 1. 併用禁止薬

試験薬の投与期間中及び最終投与後2週間以内は以下の薬剤の使用を禁止する。

1. エンシトレルビル フマル酸の併用禁忌となっている薬剤（2025年5月改訂（第22版）では以下のとおり。最新の添付文書を確認すること）

ピモジド、キニジン硫酸塩水和物、ベプリジル塩酸塩水和物、チカグレロル、エプレレノン、エルゴタミン酒石酸塩・無水カフェイン・イソプロピルアン チピリン、エルゴメトリンマレイン酸塩、メチルエルゴメトリンマレイン酸塩、ジヒドロエルゴタミンメシル酸塩、シンバスタチン、トリアゾラム、アナモレリン塩酸塩、イバブラジン塩酸塩、ベネトクラクス〔再発又は難治性の慢性リンパ 性白血病（小リンパ球性リンパ腫を含む）の用量漸増期〕、イブルチニブ、ブロナンセリン、ルラシドン塩酸塩、アゼルニジピン、アゼルニジピン・オルメサルタン メドキソミル、スボレキサント、ダリドレキサント塩酸塩、タダラフィル、マシテンタン・タダラフィル、バルデナフィル塩酸塩水和物、ロミタピドメシル酸塩、リファブチン、フィネレノン、ボクロスポリン、ロナファルニブ、マバカムテン、リバーロキサバン、リオシグアト、アパルタミド、カルバマゼピン、エンザルタミド、ミトタン、フェニトイン、ホスフェニトインナトリウム水和物、リファンピシン、セイヨウオトギリソウ（St.John'sWort、セント・ ジョーンズ・ワート）含有食品

1. COVID-19への効能効果を有する抗ウイルス薬（レムデシビル、モルヌピラビル、ニルマトレルビル／リトナビル等）、中和抗体薬（ソトロビマブ、カシリビマブ／イムデビマブ、チキサゲビマブ／シルガビマブ等）
2. 抗IL-6抗体製剤（トシリズマブ等）、JAK阻害薬（バリシチニブ等。外用を除く）、内服、坐薬又は注射用のステロイド薬

【併用禁止薬の設定根拠】

（１）研究対象者の安全を確保するため。

（２）（３）本研究での有効性評価に影響する可能性があるため。

1. 時点ごとの観察、検査及び評価

研究責任医師又は研究分担医師は、以下の観察・検査・評価スケジュールに従って、データを収集する。

- 1. 観察・検査・評価スケジュール
     1. 登録時（Visit 1）
- 研究対象者の背景
- 身長・体重
- 併存疾患
- 既往疾患
- オンライン面談
- 重症化リスク因子の有無
- 併用薬の確認
- 妊娠の確認
  - 1. 試験薬服用開始日
- QOL
- 労働生産性
  - 1. 1週後（Visit 2）
- オンライン面談
- 試験薬の服用状況
- 併用薬の確認
- 妊娠の確認
- 有害事象
  - 1. 1ヵ月後（Visit 3）、2ヵ月後（Visit 4）、3ヵ月後（Visit 5）、4ヵ月後（Visit 6）、5ヵ月後（Visit 7）、6ヵ月後（Visit 8）
- 罹患後症状
- QOL
- 労働生産性
- COVID-19再罹患について
  - 1. 追跡期間中止時
- 罹患後症状（可能な限り）
- QOL（可能な限り）
- 労働生産性スコア（可能な限り）
- COVID-19再罹患について（可能な限り）
  1. スタディーカレンダー

|  | 前観察期 | 治療期 | | 追跡期間 | | | | | | | |
| --- | --- | --- | --- | --- | --- | --- | --- | --- | --- | --- | --- |
|  | 登録時 | 試験薬 服用 開始日 | 1週後^*2^ | | 1ヵ月後 | 2ヵ月後 | 3ヵ月後 | 4ヵ月後 | 5ヵ月後 | ６ヵ月後 | 中止時 |
| Visit | 1 | - | 2 | | 3 | 4 | 5 | 6 | 7 | 8 |  |
| Day |  | 0 | 7 | | 28 | 56 | 84 | 112 | 140 | 168 |  |
| 許容範囲 | －^*1^ | ― | －2～+5 | | ±7 | ±7 | ±7 | ±7 | ±7 | ±7 |  |
| 同意取得 | ● |  |  | |  |  |  |  |  |  |  |
| 登録及び割付 | ● |  |  | |  |  |  |  |  |  |  |
| 研究対象者の背景 | ● |  |  | |  |  |  |  |  |  |  |
| 身長・体重 | ● |  |  | |  |  |  |  |  |  |  |
| 併存疾患 | ● |  |  | |  |  |  |  |  |  |  |
| 既往疾患 | ● |  |  | |  |  |  |  |  |  |  |
| 試験薬の服用開始 |  | ● |  | |  |  |  |  |  |  |  |
| 試験薬服用記録 |  |  |  | |  |  |  |  |  |  |  |
| オンライン面談 | ● |  | ● | |  |  |  |  |  |  |  |
| 重症化リスク因子の有無 | ● |  |  | |  |  |  |  |  |  |  |
| 併用薬の確認 | ● |  | ● | |  |  |  |  |  |  |  |
| 妊娠の確認 | ● |  |  | |  |  |  |  |  |  |  |
| 罹患後症状 |  |  |  | | ● | ● | ● | ● | ● | ● | 〇 |
| QOL |  | ● |  | | ● | ● | ● | ● | ● | ● | 〇 |
| 労働生産性 |  | ● |  | | ● | ● | ● | ● | ● | ● | 〇 |
| 医療機関の受診や薬の処方について |  |  |  | | ● | ● | ● | ● | ● | ● | 〇 |
| COVID-19再罹患について |  |  |  | | ● | ● | ● | ● | ● | ● | 〇 |
| 有害事象^*3^ |  |  |  | |  |  |  |  |  |  |  |

●：必須項目、〇：可能な限り実施する項目

*1：発症から72時間以内に試験薬が服用開始できるように対応すること。

*2：試験薬服用又は研究が中止された場合、可能な限りオンライン面談を実施する。

*3：有害事象の収集は、Day 0より開始し、試験薬最終投与の2週間後まで実施する。

1. 観察・検査及び評価の手順
   1. 本研究で使用するDecentralized Clinical Trials支援システム

| システム名（提供会社） | 概要 |
| --- | --- |
| MiROHA  （株式会社MICIN） | ブラウザでシステムにアクセスし、研究責任医師、研究分担医師及び研究対象者がオンライン面談、eConsentに使用する。  また、研究責任医師及び研究分担医師がデータの記録に使用する。 |
| スタディ・コンシェルジュ  （株式会社Buzzreach） | 研究対象者のスマートフォンにインストールしたアプリを用いて、研究対象者が服用状況、罹患後症状、QOL、労働生産性等について記録する。 |

- 1. 研究対象者の募集

COVID-19疑いの患者が受診した場合、研究対象者紹介医療機関の医師は、核酸増幅法（PCR 法、LAMP法等）、抗原定性検査又は抗原定量検査でCOVID-19の確定診断を行う。COVID-19確定診断された患者に対し、必要に応じ対症療法を行ったうえで、本研究について研究責任医師又は研究分担医師の説明を受ける意思があるかどうかを患者に確認する。説明を受ける意思がある患者に研究対象者募集サイトを紹介する。患者は募集サイトに氏名、携帯電話番号、電子メールアドレス、住所、オンライン面談希望日時等を入力する。募集サイト入力後に発信される「入力確認メール」には同意説明文書を閲覧できるリンクを表示し、オンライン面談前に患者が同意説明文書を読めるようにする。

研究責任医師、研究分担医師又は研究協力者は募集サイトでオンライン面談希望日時を確認し、オンライン面談時刻を決定後、患者に伝達する。

- 1. 研究対象候補者登録

研究責任医師、研究分担医師又は研究協力者は、MiROHAのeConsent画面で患者の氏名及び電子メールアドレスを登録する。

- 1. 同意取得

研究責任医師又は研究分担医師は、患者にオンライン面談して研究内容を説明し、患者から同意を取得する（詳細な同意取得の手順は15.4.1項参照）。

- 1. 登録、割付及び試験薬の送付
     1. 登録、割付及び試験薬の送付の手順

1. 研究責任医師又は研究分担医師は、同意を取得した研究対象者について選択基準及び除外基準をもとに適格性を評価し、適格と判断された研究対象者についてWeb割付システムに必要事項を入力する。
2. Web割付システムにて当該研究対象者に投与する試験薬が決定される。
3. 試験薬配送センターは、割付けられた試験薬を研究対象者が指定する場所に送付する。
4. 研究対象者は、試験薬を受領した旨をスタディ・コンシェルジュに記録する。
   - 1. 割付手順の作成及び保管

割付責任者が割付手順を作成し、研究対象者の割付情報を管理する。

割付情報は安全な場所に保管され、研究対象者、研究代表医師、研究責任医師及び研究分担医師を含め、全ての関係者が入手できないようにし、盲検性を維持する。

研究全体のキーオープンは、最終解析のためのデータが固定された後に実施する。中間解析は独立データモニタリング委員会にて実施する。その際、割付の情報は独立データモニタリング委員会のみに提出し、研究対象者、研究責任医師及び研究分担医師を含めた全ての関係者は入手しない。

- - 1. 緊急キーオープン

1. 緊急キーオープンは研究対象者に重篤な有害事象等が発生し、適切な医学的治療を施すため研究対象者への処置内容を特定する必要が生じた場合等、緊急時にのみ許容される。
2. 緊急キーオープンが必要になった場合、割付手順に従い緊急キーオープンを行う。
   1. 試験薬服用記録

研究対象者は、各服用日での試験薬服用の有無及び服用時間（初回服用日のみ）をスタディ・コンシェルジュに記録する。

- 1. 研究対象者の背景

以下の調査・評価項目を確認する。

- 同意年月日
- 生年月日
- 性別
- 民族、人種
- COVID-19発症日時
- COVID-19確定診断（診断日、検査方法）
- 研究対象者紹介医療機関での確定診断時の体温
- 同意取得時点のCOVID-19の14症状（けん怠感 [疲労感]、筋肉痛又は体の痛み、頭痛、悪寒/発汗、熱っぽさ又は発熱、鼻水又は鼻づまり、喉の痛み、咳、息切れ、吐き気、嘔吐、下痢、味覚異常及び嗅覚異常)の程度
- ワクチン接種歴（接種有の場合は接種回数、最後に接種した日）
- 喫煙状況
  1. 身長・体重

研究対象者から身長、体重を聴取する。

・身長：cm表記（整数）

・体重：kg表記（整数）

身長と体重から統計解析時にBMIを算出する。

- 1. 併存疾患

同意取得時点でCOVID-19と同時に併存している別の疾患を確認する。

- 1. 既往疾患

同意取得前1年以内に入院を要した疾患及び同意取得前12週間以内に来院を要した疾患を確認する。

COVID-19罹患の有無、罹患時期を確認する。

- 1. 重症化リスク因子の有無

年齢、性別、BMI、併存疾患等の情報から、重症化リスク因子の有無を評価する。

- 1. 併用薬の確認

併用薬（薬剤名、服用期間、使用理由）を研究対象者に確認する。

- 1. 妊娠の有無

女性では、問診にて妊娠の有無を確認する。

- 1. 罹患後症状

研究対象者は、罹患後症状の重症度等（付録1）をスタディ・コンシェルジュに入力する

- 1. QOL

研究対象者は、EQ-5D-5L（付録2）の回答をスタディ・コンシェルジュに入力する。

- 1. 労働生産性

研究対象者は、Work Productivity and Activity Impairment Questionnaire：General Health日本語版（付録3）の回答をスタディ・コンシェルジュに入力する。

- 1. 医療機関の受診や薬の処方について

研究対象者は、医療機関の受診や薬の処方に関する質問（付録4）の回答をスタディ・コンシェルジュに入力する。

- 1. COVID-19再罹患について

研究対象者は、COVID-19再罹患に関する質問（付録5）の回答をスタディ・コンシェルジュに入力する。

- 1. 研究対象者ごとの中止及び終了の基準
     1. 研究対象者ごとの中止

研究責任医師又は研究分担医師は、研究対象者の登録後に以下の事項に該当することが判明した場合、当該研究対象者に対する試験薬服用を中止する。試験薬中止症例は所定の中止時評価を行い、追跡期間に移行する。

1. 有害事象が発現し、本研究の継続により研究対象者の健康に容認できないリスクが生じると研究責任医師又は研究分担医師が判断した場合
2. 研究対象者から試験薬服用をやめたいとの申し出があった場合
3. エンシトレルビル フマル酸の併用禁忌となっている薬剤の投与が必要になった場合
4. その他のCOVID-19治療薬投与や呼吸療法が必要になった場合、又は併存疾患のために当該疾患の効能も有するCOVID-19治療薬の投与が必要になった場合
5. 研究対象者として不適切であることが判明した場合
6. 女性研究対象者の妊娠が判明した場合
7. その他、研究責任医師又は研究分担医師が介入を中止すべきであると判断した場合

研究責任医師又は研究分担医師は、研究対象者の登録後に以下の事項に該当することが判明した場合、当該研究対象者に対する研究を中止する。

1. 研究対象者から研究中止の申し出があった場合
2. スマートフォンの故障等、研究対象者の都合で必要な観察の今後の実施が不可能であることが判明した場合
3. 追跡期間中に研究対象者がCOVID-19に再度罹患したことが判明した場合
4. 試験薬服用前に研究対象者として不適切であることが判明した場合
5. その他、研究責任医師又は研究分担医師が研究を中止すべきであると判断した場合
   - 1. 研究対象者ごとの中止の手順

研究責任医師又は研究分担医師は、中止基準に該当することが判明した場合には、ただちにその旨を研究対象者に説明し、必要に応じ代替治療等の処置を講じる。

治療期での中止の場合、研究責任医師又は研究分担医師は、可能な限り本研究で計画されている観察・評価をVisit 2のオンライン面談時に実施し、中止理由とともにその結果を症例報告書に記録する。

追跡期間での中止の場合、研究対象者は、可能な限り本研究で計画されている評価を実施し、中止理由とともにスタディ・コンシェルジュに入力する。

- - 1. 研究対象者ごとの終了

本研究計画書に記載した計画したすべての観察・検査・評価が完了することをもって、当該研究対象者の終了とする。

1. 有害事象
   1. 有害事象の定義

有害事象とは、研究対象者に生じた、あらゆる好ましくない或いは意図しない徴候（臨床検査値の異常を含む）、症状又は病気のことであり、本研究との因果関係の有無は問わない。研究開始前から認められ、投与後にその症状が増悪した場合も有害事象として扱う。

- 1. 有害事象の収集期間

有害事象の収集は、試験薬の服用開始時より開始し、試験薬最終投与の2週間後まで実施する。

- 1. 自覚症状の確認

研究責任医師又は研究分担医師は、オンライン面談時に研究対象者から自覚症状の発現の有無を確認する。

- 1. 有害事象の評価

研究責任医師又は研究分担医師は、収集期間中に観察された有害事象について以下を症例報告書に記録する。有害事象が複数観察された場合には、事象ごとに記録する。

1. 有害事象名
2. 発現日
3. 重症度
4. 重篤性（非重篤、重篤）
5. 研究との因果関係
6. 試験薬との因果関係
7. 転帰日
8. 転帰
   - 1. 有害事象名

各事象を診断名で記載する。その診断名に随伴する徴候（臨床検査値の異常、心電図の異常所見を含む）及び症状は別の有害事象としては記載しない。診断名が不明な場合は、適宜、その徴候又は症状を有害事象として記載する。

- - 1. 発現日

有害事象の発現日を以下の規準で判断する。

| 有害事象 | 発現日 |
| --- | --- |
| 徴候、症状、疾患（診断名）の場合 | 研究対象者又は研究責任医師又は研究分担医師が最初に有害事象の徴候、症状に気付いた日を記載する |
| 無症候性の疾患の場合 | 診断のために検査が実施され、診断が確定した日を記載する  検査所見から陳旧性の所見が見られる場合や、発生時期がおおよそ推定できる場合でも、診断が確定した日を記載する |
| 併存疾患の悪化の場合 | 研究対象者又は研究責任医師又は研究分担医師が最初に疾患、症状の悪化に気付いた日を記載する |
| 本研究の対象となる医薬品等を適用開始後の検査で異常となった場合 | 臨床的に問題があると判断される検査値異常が認められた検査日を記載する |
| 本研究の対象となる医薬品等を適用開始する時点の検査で異常が見られ、その後の検査で悪化した場合 | 検査値の推移より医学的判断で明らかな上昇、低下、増加、減少が認められた検査日を記載する |

- - 1. 重症度

有害事象の重症度を次のように分類する。

| 軽　度 | 一過性で容易に耐えられるもの |
| --- | --- |
| 中等度 | 通常の活動に支障をきたす程度のもの |
| 高　度 | 通常の活動を不可能にする程度のもの |

- - 1. 重篤性

有害事象の重症度に関わらず、次のいずれかに該当するものを重篤と判定する。

1. 死亡
2. 死亡につながるおそれ
3. 治療のために医療機関への入院又は入院期間の延長
4. 障害
5. 障害につながるおそれ
6. 上記に準じて重篤
7. 後世代における先天性の疾病又は異常
   - 1. 研究との因果関係

本研究の実施と有害事象との因果関係を次のように分類する。なお、因果関係なしと判定した場合は、判定理由を症例報告書に記録する。

| 関連あり | 有害事象の原因が本研究の実施によるものであることが合理的に説明できるもの |
| --- | --- |
| 関連なし | 有害事象の原因が本研究の実施によるものであることが合理的に説明できないもの |

合理的に説明できるものの事例については、10.4.6項参照。

- - 1. 試験薬との因果関係

試験薬と有害事象との因果関係を次のように分類する。なお、因果関係なしと判定した場合は、判定理由を症例報告書に記録する。

| 関連あり | 有害事象の原因が試験薬であることが合理的に説明できるもの |
| --- | --- |
| 関連なし | 有害事象の原因が試験薬であることが合理的に説明できないもの |

有害事象の原因が試験薬であることが合理的に説明できるものとは、例えば、類似の有害事象の報告がある等試験薬の薬理作用から説明できる場合、投与量の増減が有害事象の発現や重篤度に影響する場合、あるいは既往歴/合併症や併用薬等の情報は可能な限り収集し、それらを検討しても、試験薬以外の要因から説明ができない場合等を言う。

- - 1. 転帰日

症状が消失又は回復した日、又は有害事象の転帰を判定した日とする。転帰が「死亡」の場合には、死亡日とする。

- - 1. 転帰

有害事象の転帰を次のように分類する

| 区分 | 判断規準 |
| --- | --- |
| 回復 | ・症状の消失又は回復  ・検査値の正常化又は投与前の検査値への回復 |
| 軽快 | ・症状がほぼ消失  ・検査値の改善が認められたが、正常化又は投与前の検査値に回復していない  ・当該有害事象が直接の死因でない死亡例で、当該有害事象が軽快のまま死亡 |
| 未回復 | ・症状や検査値に変化がない  ・観察できた期間の最後の日の症状、所見や検査値が発現時の重症度より悪化  ・不可逆性の先天異常  ・当該有害事象が直接の死因でない死亡例で、当該有害事象が未回復のまま死亡 |
| 回復したが後遺症あり | ・日常生活に支障をきたす程度の機能不全が残った |
| 死亡 | ・死亡と当該有害事象との間に直接の関連性が認められた  「直接の関連性が認められた」とは、当該有害事象が死亡の原因になった、又は当該有害事象が明らかに死亡に寄与したことをさす  ・同一症例で見られた直接の死因ではないと判定（判断、推定）される有害事象の転帰については、死亡としない |
| 不明 | ・発現日以降の経過が、転院、転居等により本研究計画書に記載されている追跡が不可能となった |

- 1. 有害事象が発現した場合の措置
     1. 研究対象者への処置

有害事象の発現に際しては、研究責任医師及び研究分担医師は、当該研究対象者を紹介した医療機関の受診を研究対象者に推奨する。必要に応じ専門医師による診断・治療を受けさせることにより解決及び原因究明に努める。

- - 1. 有害事象の追跡調査

研究期間中に発現した有害事象は回復するか、又は臨床上必要がないと判断されるまで、可能な限り電話で追跡する。

- - 1. 資金提供企業への報告

有害事象が発現した場合には、研究責任医師又は研究分担医師は、資金提供者である塩野義製薬業株式会社が指定する手順に従い報告する。報告にあたっては、研究対象者を特定できる情報を含まないようにする。

- 1. 本研究で予想される有害事象

エンシトレルビル フマル酸の添付文書（2024年7月改訂（第14版））に記載されている副作用は以下の通り。研究責任医師又は研究分担医師は試験薬の投与にあたり、最新の添付文書を確認する。

(1) 重大な副作用

ショック、アナフィラキシー（頻度不明）

(2) その他の副作用

| 種類＼頻度 | 5%以上 | 1～5%未満 | 1%未満 | 頻度不明 |
| --- | --- | --- | --- | --- |
| 過敏症 |  |  | 発疹 | そう痒 |
| 消化器 |  |  | 悪心、嘔吐、下痢、腹部不快感 |  |
| 精神神経系 |  |  | 頭痛 |  |
| 代謝 |  |  | 脂質異常症 |  |
| その他 | HDLコレステロール低下（16.6%） | トリグリセリド上昇、ビリルビン上昇、血中コレステロール低下 | 血清鉄上昇 |  |

1. 疾病等
   1. 疾病等の定義

疾病等とは、本研究の実施に起因するものと疑われる疾病、障害若しくは死亡又は感染症に加え、臨床検査値の異常や諸症状を含む。

- 1. 重篤な疾病等の認定臨床研究審査委員会への報告手順

以下の1．から3．の報告対象となる疾病等が発生した場合は、担当医は速やかに研究責任医師に伝える。研究責任医師は、次に掲げる事項を知ったときは、それぞれに定める期間内に実施医療機関の管理者に報告した上で、研究代表医師に通知し、研究代表医師は認定臨床研究審査委員会に報告するとともに、他の研究責任医師にその旨を情報提供する。情報提供を受けた他の研究責任医師は、速やかにその内容を実施医療機関の管理者に報告する。

1．次のうち、本研究の実施によるものと疑われるものであって予測できないもの：7日

イ．死亡

ロ．死亡につながる恐れのある疾病等

2．次に掲げる事項：15日

イ．次のうち、本研究の実施によるものと疑われるもの（1に掲げるものを除く）

（１）死亡

（２）死亡につながる恐れのある疾病等

ロ．次のうち、本研究の実施によるものと疑われるものであって予測できないもの

（１）治療のために医療機関への入院又は入院期間の延長が必要とされる疾病等

（２）障害

（３）障害につながるおそれのある疾病等

（４）（１）から（３）まで並びに死亡及び死亡につながるおそれのある疾病等に準じて重篤である疾病等

（５）後世代における先天性の疾病又は異常

3．次のうち、本研究の実施によるものと疑われるもの（前号ロに掲げるもの及び効果安全性評価委員会が設置された特定臨床研究において発生したものを除く。）：30日

イ．治療のために医療機関への入院又は入院期間の延長が必要とされる疾病等

ロ．障害

ハ．障害につながるおそれのある疾病等

ニ．イからハまで並びに死亡及び死亡につながるおそれのある疾病等に準じて重篤である疾病等

ホ．後世代における先天性の疾病又は異常

4．本研究の実施によるものと疑われるもの（上記1～3以外）：法第十七条第一項の規定による認定臨床研究審査委員会への定期報告を行うとき

●認定臨床研究審査委員会への報告対象となる疾病等

| 疾病等 | 予測できないもの | 予測できるもの |
| --- | --- | --- |
| a.　死亡 | 7日 | 15日 |
| b.　死亡につながるおそれのある疾病等 | 7日 | 15日 |
| c.　治療のために医療機関への入院又は入院期間の延長が必要とされる疾病等 | 15日 | 30日 |
| d.　障害 | 15日 | 30日 |
| e.　障害につながるおそれのある疾病等 | 15日 | 30日 |
| f.　cからeまで並びに死亡及び死亡につながるおそれのある疾病等に準じて重篤である疾病等 | 15日 | 30日 |
| g.　後世代における先天性の疾病又は異常 | 15日 | 30日 |
| h.　その他の疾病等 | 定期報告 | 定期報告 |

研究責任医師又は研究分担医師は、転帰の変更等、疾病等の報告内容に変更が生じた場合、実施医療機関の管理者及び研究代表医師に通知し、研究代表医師は認定臨床研究審査委員会に報告する。

- 1. 重篤な疾病等の厚生労働大臣への報告手順

研究代表医師は、11.2項の1及び2のロの事項を知った時には、同項の表に定める期間内に、jRCTの疾病等報告画面より厚生労働大臣に報告する。

1. データマネジメント
   1. データマネジメント計画

症例報告書での欠測値、未記入値、誤記が疑われる記載等に関して、データマネジメント担当者は、研究責任医師又は研究分担医師に問い合わせする。研究責任医師又は研究分担医師による回答又はデータ修正の内容を確認する。

- 1. 症例報告書

研究責任医師又は研究分担医師は、同意を取得したすべての研究対象者について症例報告書を作成する。

症例報告書の作成にあたっては、MiROHA及びWeb割付システムに入力する。

症例報告書の変更又は修正は、変更又は修正前後の情報、変更又は修正者、変更又は修正日及びその理由を記録した監査証跡として記録される。

研究責任医師は、症例報告書に入力された全データに関する正確性と信頼性について全責任を負う。

症例報告書及びスタディ・コンシェルジュに入力されたすべてのデータを原資料とする。

1. 統計的事項

詳細なデータの取扱い及び解析内容については、別途「統計解析計画書」に定める。尚、統計解析計画書は中間解析時までに固定する。

- 1. 解析集団の定義

本研究では、解析対象集団として「最大の解析対象集団（Full Analysis Set：FAS）」及び「安全性解析対象集団（Safety Analysis Set：SAS）」の2種類の解析対象集団を設ける。

FASは、試験薬を少なくとも1回以上服用し、1時点以上の観察、検査及び評価を実施した研究対象者と定義する。

SASは、試験薬を少なくとも1回以上服用した研究対象者と定義する。

- 1. データの取り扱い

データの取扱いは以下のとおりとする。なお、統計解析担当責任者は研究代表医師及びデータマネジメント担当者と協議の上、データ固定前にすべての研究対象者に関するデータの解析上の取扱いを決定する。

- - 1. 規定された許容範囲外の測定値の取扱い

評価項目の測定許容範囲から逸脱したデータに関しては、統計解析担当責任者が研究代表医師及びデータマネジメント担当者と協議の上でその採否を決定する。なお、該当範囲内に複数のデータが存在する場合には、規定評価日との日数差の絶対値を算出し、絶対値が最小のものをその評価時期のデータとして採用する。なお、絶対値が同じ場合には個別（評価項目別）に検討する。

- - 1. 欠測値の取扱い

欠測したデータについては補完しない。

- 1. 解析方法
     1. 研究対象者の背景

解析項目　　：研究対象者の背景

解析対象集団：FAS、SAS

解析方法　　：人口統計学的及びその他の特性について、記述統計量を用いて要約する。

連続変数については研究対象者数、平均値、標準偏差、最小値、中央値、最大値を算出する。カテゴリ変数については研究対象者数、頻度及び割合を算出する。

- - 1. 試験薬服用の状況

解析項目　　：試験薬服用の状況

解析対象集団：FAS、SAS

解析方法　　：試験薬を服用した期間、服用中止の有無、服用中止理由に関して要約する。

- - 1. 併用薬

解析項目　　：併用薬

解析対象集団：FAS、SAS

解析方法　　：併用薬に関して要約する。

- - 1. 有効性主要評価項目

解析項目　　：「治療開始から1ヵ月及び3ヵ月の2時点両方で、けん怠感（疲労感）、息切れ又は呼吸困難感、嗅覚の異常、味覚の異常のいずれかの症状がある」又は「3ヵ月時点で集中力・思考力の低下、課題解決力の低下、物忘れ（短期又は長期）のいずれかの症状がある」研究対象者の割合

解析対象集団：FAS

解析方法　　：「治療開始から1ヵ月及び3ヵ月の2時点両方で、けん怠感（疲労感）、息切れ又は呼吸困難感、嗅覚の異常、味覚の異常のいずれかの症状がある」又は「3ヵ月時点で集中力・思考力の低下、課題解決力の低下、物忘れ（短期又は長期）のいずれかの症状がある」のいずれかの有無を応答変数とし、治療薬群、COVID-19ワクチン接種歴の有無、同意取得時のCOVID-19症状（14症状）の重症度（9点未満、9点以上）、年齢（連続値）、性別、BMI（25未満、25以上）を説明変数とした修正ポアソン回帰モデルを適用する。調整リスク比を算出しプラセボ群と比較する。

- - 1. 有効性副次評価項目

解析項目　　：「治療開始から1ヵ月及び3ヵ月の2時点両方で、けん怠感（疲労感）、息切れ又は呼吸困難感、嗅覚の異常、味覚の異常のいずれかの症状がある」又は「3ヵ月時点で集中力・思考力の低下、課題解決力の低下、物忘れ（短期又は長期）のいずれかの症状がある」研究対象者の割合（COVID-19と関連がある、又は関連不明）

解析対象集団：FAS

解析方法　　：「治療開始から1ヵ月及び3ヵ月の2時点両方で、研究対象者自身がCOVID-19と関連がある、又は関連不明と判断したけん怠感（疲労感）、息切れ又は呼吸困難感、嗅覚の異常、味覚の異常のいずれかの症状がある」又は「3ヵ月時点で研究対象者自身がCOVID-19と関連がある、又は関連不明と判断した集中力・思考力の低下、課題解決力の低下、物忘れ（短期又は長期）のいずれかの症状がある」のいずれかの有無を応答変数とし、治療薬群、COVID-19ワクチン接種歴の有無、同意取得時のCOVID-19症状（14症状）の重症度（9点未満、9点以上）、年齢（連続値）、性別、BMI（25未満、25以上）を説明変数とした修正ポアソン回帰モデルを適用する。調整リスク比を算出しプラセボ群と比較する。

解析項目　　：「治療開始から1ヵ月及び3ヵ月及び6ヵ月の3時点全てで、けん怠感（疲労感）、息切れ又は呼吸困難感、嗅覚の異常、味覚の異常のいずれかの症状がある」又は「6ヵ月時点で集中力・思考力の低下、課題解決力の低下、物忘れ（短期又は長期）のいずれかの症状がある」研究対象者の割合

解析対象集団：FAS

解析方法　　：「治療開始から1ヵ月及び3ヵ月及び6ヵ月の3時点全てで、けん怠感（疲労感）、息切れ又は呼吸困難感、嗅覚の異常、味覚の異常のいずれかの症状がある」又は「6ヵ月時点で集中力・思考力の低下、課題解決力の低下、物忘れ（短期又は長期）のいずれかの症状がある」のいずれかの有無を応答変数とし、治療薬群、COVID-19ワクチン接種歴の有無、同意取得時のCOVID-19症状（14症状）の重症度（9点未満、9点以上）、年齢（連続値）、性別、BMI（25未満、25以上）を説明変数とした修正ポアソン回帰モデルを適用する。調整リスク比を算出しプラセボ群と比較する。

解析項目　　：「治療開始から1ヵ月及び3ヵ月及び6ヵ月の3時点全てで、けん怠感（疲労感）、息切れ又は呼吸困難感、嗅覚の異常、味覚の異常のいずれかの症状がある」又は「6ヵ月時点で集中力・思考力の低下、課題解決力の低下、物忘れ（短期又は長期）のいずれかの症状がある」研究対象者の割合（COVID-19と関連がある、又は関連不明）

解析対象集団：FAS

解析方法　　：「治療開始から1ヵ月及び3ヵ月及び6ヵ月の3時点全てで、研究対象者自身がCOVID-19と関連がある、又は関連不明と判断したけん怠感（疲労感）、息切れ又は呼吸困難感、嗅覚の異常、味覚の異常のいずれかの症状がある」又は「6ヵ月時点で研究対象者自身がCOVID-19と関連がある、又は関連不明と判断した集中力・思考力の低下、課題解決力の低下、物忘れ（短期又は長期）のいずれかの症状がある」のいずれかの有無を応答変数とし、治療薬群、COVID-19ワクチン接種歴の有無、同意取得時のCOVID-19症状（14症状）の重症度（9点未満、9点以上）、年齢（連続値）、性別、BMI（25未満、25以上）を説明変数とした修正ポアソン回帰モデルを適用する。調整リスク比を算出しプラセボ群と比較する。

解析項目　　：治療開始から1ヵ月及び3ヵ月の2時点両方で、けん怠感（疲労感）、息切れ又は呼吸困難感、嗅覚の異常、味覚の異常のいずれかの症状がある研究対象者の割合

解析対象集団：FAS

解析方法　　：治療開始から1ヵ月及び3ヵ月の2時点両方で、けん怠感（疲労感）、息切れ又は呼吸困難感、嗅覚の異常、味覚の異常のいずれかの症状の有無を応答変数とし、治療薬群、COVID-19ワクチン接種歴の有無、同意取得時のCOVID-19症状（14症状）の重症度（9点未満、9点以上）、年齢（連続値）、性別、BMI（25未満、25以上）を説明変数とした修正ポアソン回帰モデルを適用する。調整リスク比を算出しプラセボ群と比較する。

解析項目　　：治療開始から1ヵ月及び3ヵ月の2時点両方で、けん怠感（疲労感）、息切れ又は呼吸困難感、嗅覚の異常、味覚の異常のいずれかの症状がある研究対象者の割合（COVID-19と関連がある、又は関連不明）

解析対象集団：FAS

解析方法　　：治療開始から1ヵ月及び3ヵ月の2時点両方で、研究対象者自身がCOVID-19と関連がある、又は関連不明と判断したけん怠感（疲労感）、息切れ又は呼吸困難感、嗅覚の異常、味覚の異常のいずれかの症状の有無を応答変数とし、治療薬群、COVID-19ワクチン接種歴の有無、同意取得時のCOVID-19症状（14症状）の重症度（9点未満、9点以上）、年齢（連続値）、性別、BMI（25未満、25以上）を説明変数とした修正ポアソン回帰モデルを適用する。調整リスク比を算出しプラセボ群と比較する。

解析項目　　：治療開始から1ヵ月及び3ヵ月の2時点両方で、けん怠感（疲労感）、息切れ又は呼吸困難感、嗅覚の異常、味覚の異常の各症状がある研究対象者の割合

解析対象集団：FAS

解析方法　　：治療開始から1ヵ月及び3ヵ月の2時点両方で、けん怠感（疲労感）、息切れ又は呼吸困難感、嗅覚の異常、味覚の異常の各症状それぞれの有無を応答変数とし、治療薬群、COVID-19ワクチン接種歴の有無、同意取得時のCOVID-19症状（14症状）の重症度（9点未満、9点以上）、年齢（連続値）、性別、BMI（25未満、25以上）を説明変数とした修正ポアソン回帰モデルを適用する。調整リスク比を算出しプラセボ群と比較する。

解析項目　　：治療開始から1ヵ月及び3ヵ月の2時点両方で、けん怠感（疲労感）、息切れ又は呼吸困難感、嗅覚の異常、味覚の異常の各症状がある研究対象者の割合（COVID-19と関連がある、又は関連不明）

解析対象集団：FAS

解析方法　　：治療開始から1ヵ月及び3ヵ月の2時点両方で、研究対象者自身がCOVID-19と関連がある、又は関連不明と判断したけん怠感（疲労感）、息切れ又は呼吸困難感、嗅覚の異常、味覚の異常の各症状それぞれの有無を応答変数とし、治療薬群、COVID-19ワクチン接種歴の有無、同意取得時のCOVID-19症状（14症状）の重症度（9点未満、9点以上）、年齢（連続値）、性別、BMI（25未満、25以上）を説明変数とした修正ポアソン回帰モデルを適用する。調整リスク比を算出しプラセボ群と比較する。

解析項目　　：治療開始から3ヵ月時点で集中力・思考力の低下、課題解決力の低下、物忘れ（短期又は長期）のいずれかの症状がある研究対象者の割合

解析対象集団：FAS

解析方法　　：治療開始から3ヵ月時点で集中力・思考力の低下、課題解決力の低下、物忘れ（短期又は長期）のいずれかの症状の有無を応答変数とし、治療薬群、COVID-19ワクチン接種歴の有無、同意取得時のCOVID-19症状（14症状）の重症度（9点未満、9点以上）、年齢（連続値）、性別、BMI（25未満、25以上）を説明変数とした修正ポアソン回帰モデルを適用する。調整リスク比を算出しプラセボ群と比較する。

解析項目　　：治療開始から3ヵ月時点で集中力・思考力の低下、課題解決力の低下、物忘れ（短期又は長期）のいずれかの症状がある研究対象者の割合（COVID-19と関連がある、又は関連不明）

解析対象集団：FAS

解析方法　　：治療開始から3ヵ月時点で研究対象者自身がCOVID-19と関連がある、又は関連不明と判断した集中力・思考力の低下、課題解決力の低下、物忘れ（短期又は長期）のいずれかの症状の有無を応答変数とし、治療薬群、COVID-19ワクチン接種歴の有無、同意取得時のCOVID-19症状（14症状）の重症度（9点未満、9点以上）、年齢（連続値）、性別、BMI（25未満、25以上）を説明変数とした修正ポアソン回帰モデルを適用する。調整リスク比を算出しプラセボ群と比較する。

解析項目　　：治療開始から3ヵ月時点で集中力・思考力の低下、課題解決力の低下、物忘れ（短期又は長期）の各症状がある研究対象者の割合

解析対象集団：FAS

解析方法　　：治療開始から3ヵ月時点で集中力・思考力の低下、課題解決力の低下、物忘れ（短期又は長期）の各症状の有無を応答変数とし、治療薬群、COVID-19ワクチン接種歴の有無、同意取得時のCOVID-19症状（14症状）の重症度（9点未満、9点以上）、年齢（連続値）、性別、BMI（25未満、25以上）を説明変数とした修正ポアソン回帰モデルを適用する。調整リスク比を算出しプラセボ群と比較する。

解析項目　　：治療開始から3ヵ月時点で集中力・思考力の低下、課題解決力の低下、物忘れ（短期又は長期）の各症状がある研究対象者の割合（COVID-19と関連がある、又は関連不明）

解析対象集団：FAS

解析方法　　：治療開始から3ヵ月時点で研究対象者自身がCOVID-19と関連がある、又は関連不明と判断した集中力・思考力の低下、課題解決力の低下、物忘れ（短期又は長期）の各症状の有無を応答変数とし、治療薬群、COVID-19ワクチン接種歴の有無、同意取得時のCOVID-19症状（14症状）の重症度（9点未満、9点以上）、年齢（連続値）、性別、BMI（25未満、25以上）を説明変数とした修正ポアソン回帰モデルを適用する。調整リスク比を算出しプラセボ群と比較する。

解析項目　　：治療開始から3ヵ月時点でCOVID-19 罹患前同様の通常の健康状態に戻っておらず、COVID-19の14症状（けん怠感（疲労感）、体の痛み又は筋肉痛、頭痛、悪寒、熱っぽさ、鼻水もしくは鼻づまり、喉の痛み、咳、息切れ又は呼吸困難、吐き気、嘔吐、下痢、嗅覚の異常、味覚の異常）のいずれかの症状がある研究対象者の割合

解析対象集団：FAS

解析方法　　：治療開始から3ヵ月時点でCOVID-19 罹患前同様の通常の健康状態に戻っておらず、COVID-19の14症状（けん怠感（疲労感）、体の痛み又は筋肉痛、頭痛、悪寒、熱っぽさ、鼻水もしくは鼻づまり、喉の痛み、咳、息切れ又は呼吸困難、吐き気、嘔吐、下痢、嗅覚の異常、味覚の異常）のいずれかの症状の有無を応答変数とし、治療薬群、COVID-19ワクチン接種歴の有無、同意取得時のCOVID-19症状（14症状）の重症度（9点未満、9点以上）、年齢（連続値）、性別、BMI（25未満、25以上）を説明変数とした修正ポアソン回帰モデルを適用する。調整リスク比を算出しプラセボ群と比較する。

解析項目　　：治療開始から3ヵ月時点でCOVID-19 罹患前同様の通常の健康状態に戻っておらず、神経学的4症状（集中力・思考力の低下、課題解決力の低下、物忘れ（短期又は長期）、不眠）のいずれかの症状がある研究対象者の割合

解析対象集団：FAS

解析方法　　：治療開始から3ヵ月時点でCOVID-19 罹患前同様の通常の健康状態に戻っておらず、神経学的4症状（集中力・思考力の低下、課題解決力の低下、物忘れ（短期又は長期）、不眠）のいずれかの症状の有無を応答変数とし、治療薬群、COVID-19ワクチン接種歴の有無、同意取得時のCOVID-19症状（14症状）の重症度（9点未満、9点以上）、年齢（連続値）、性別、BMI（25未満、25以上）を説明変数とした修正ポアソン回帰モデルを適用する。調整リスク比を算出しプラセボ群と比較する。

解析項目　　：治療開始から3ヵ月時点でCOVID-19 罹患前同様の通常の健康状態に戻っておらず、けん怠感（疲労感）、体の痛み又は筋肉痛、頭痛、悪寒、熱っぽさ、鼻水もしくは鼻づまり、喉の痛み、咳、息切れ又は呼吸困難、吐き気、嘔吐、下痢、嗅覚の異常、味覚の異常、筋力低下、集中力・思考力の低下、課題解決力の低下、物忘れ（短期又は長期）、不眠、脱毛、動悸又は心拍数の増加、関節痛、食欲不振、めまい又は平衡感覚の異常、胸痛、皮膚の発疹のいずれかの症状がある研究対象者の割合

解析対象集団：FAS

解析方法　　：治療開始から3ヵ月時点でCOVID-19 罹患前同様の通常の健康状態に戻っておらず、けん怠感（疲労感）、体の痛み又は筋肉痛、頭痛、悪寒、熱っぽさ、鼻水もしくは鼻づまり、喉の痛み、咳、息切れ又は呼吸困難、吐き気、嘔吐、下痢、嗅覚の異常、味覚の異常、筋力低下、集中力・思考力の低下、課題解決力の低下、物忘れ（短期又は長期）、不眠、脱毛、動悸又は心拍数の増加、関節痛、食欲不振、めまい又は平衡感覚の異常、胸痛、皮膚の発疹のいずれかの症状のいずれかの有無を応答変数とし、治療薬群、COVID-19ワクチン接種歴の有無、同意取得時のCOVID-19症状（14症状）の重症度（9点未満、9点以上）、年齢（連続値）、性別、BMI（25未満、25以上）を説明変数とした修正ポアソン回帰モデルを適用する。調整リスク比を算出しプラセボ群と比較する。

- - 1. 有効性探索評価項目

解析項目　　：「治療開始から2ヵ月後及び3ヵ月の2時点連続で、けん怠感（疲労感）、息切れ又は呼吸困難感、嗅覚の異常、味覚の異常のいずれかの症状がある」又は「3ヵ月時点で集中力・思考力の低下、課題解決力の低下、物忘れ（短期又は長期）のいずれかの症状がある」研究対象者の割合

解析対象集団：FAS

解析方法　　：「治療開始から2ヵ月後及び3ヵ月の2時点連続で、けん怠感（疲労感）、息切れ又は呼吸困難感、嗅覚の異常、味覚の異常のいずれかの症状がある」又は「3ヵ月時点で集中力・思考力の低下、課題解決力の低下、物忘れ（短期又は長期）のいずれかの症状がある」のいずれかの有無を応答変数とし、治療薬群、COVID-19ワクチン接種歴の有無、同意取得時のCOVID-19症状（14症状）の重症度（9点未満、9点以上）、年齢（連続値）、性別、BMI（25未満、25以上）を説明変数とした修正ポアソン回帰モデルを適用する。調整リスク比を算出しプラセボ群と比較する。

解析項目　　：「治療開始から2ヵ月後及び3ヵ月の2時点連続で、けん怠感（疲労感）、息切れ又は呼吸困難感、嗅覚の異常、味覚の異常のいずれかの症状がある」又は「3ヵ月時点で集中力・思考力の低下、課題解決力の低下、物忘れ（短期又は長期）のいずれかの症状がある」研究対象者の割合（COVID-19と関連がある、又は関連不明）

解析対象集団：FAS

解析方法　　：「治療開始から2ヵ月後及び3ヵ月の2時点連続で、研究対象者自身がCOVID-19と関連がある、又は関連不明と判断したけん怠感（疲労感）、息切れ又は呼吸困難感、嗅覚の異常、味覚の異常のいずれかの症状がある」又は「3ヵ月時点で研究対象者自身がCOVID-19と関連がある、又は関連不明と判断した集中力・思考力の低下、課題解決力の低下、物忘れ（短期又は長期）のいずれかの症状がある」のいずれかの有無を応答変数とし、治療薬群、COVID-19ワクチン接種歴の有無、同意取得時のCOVID-19症状（14症状）の重症度（9点未満、9点以上）、年齢（連続値）、性別、BMI（25未満、25以上）を説明変数とした修正ポアソン回帰モデルを適用する。調整リスク比を算出しプラセボ群と比較する。

解析項目　　：治療開始から2ヵ月及び3ヵ月の2時点連続で、けん怠感（疲労感）、息切れ又は呼吸困難感、嗅覚の異常、味覚の異常のいずれかの症状がある研究対象者の割合

解析対象集団：FAS

解析方法　　：治療開始から2ヵ月及び3ヵ月の2時点連続で、けん怠感（疲労感）、息切れ又は呼吸困難感、嗅覚の異常、味覚の異常のいずれかの症状の有無を応答変数とし、治療薬群、COVID-19ワクチン接種歴の有無、同意取得時のCOVID-19症状（14症状）の重症度（9点未満、9点以上）、年齢（連続値）、性別、BMI（25未満、25以上）を説明変数とした修正ポアソン回帰モデルを適用する。調整リスク比を算出しプラセボ群と比較する。

解析項目　　：治療開始から2ヵ月及び3ヵ月の2時点連続で、けん怠感（疲労感）、息切れ又は呼吸困難感、嗅覚の異常、味覚の異常のいずれかの症状がある研究対象者の割合（COVID-19と関連がある、又は関連不明）

解析対象集団：FAS

解析方法　　：治療開始から2ヵ月及び3ヵ月の2時点連続で、研究対象者自身がCOVID-19と関連がある、又は関連不明と判断したけん怠感（疲労感）、息切れ又は呼吸困難感、嗅覚の異常、味覚の異常のいずれかの症状の有無を応答変数とし、治療薬群、COVID-19ワクチン接種歴の有無、同意取得時のCOVID-19症状（14症状）の重症度（9点未満、9点以上）、年齢（連続値）、性別、BMI（25未満、25以上）を説明変数とした修正ポアソン回帰モデルを適用する。調整リスク比を算出しプラセボ群と比較する。

解析項目　　：治療開始から2ヵ月及び3ヵ月の2時点連続で、けん怠感（疲労感）、息切れ又は呼吸困難感、嗅覚の異常、味覚の異常の各症状がある研究対象者の割合

解析対象集団：FAS

解析方法　　：治療開始から2ヵ月及び3ヵ月の2時点連続で、けん怠感（疲労感）、息切れ又は呼吸困難感、嗅覚の異常、味覚の異常の各症状の有無を応答変数とし、治療薬群、COVID-19ワクチン接種歴の有無、同意取得時のCOVID-19症状（14症状）の重症度（9点未満、9点以上）、年齢（連続値）、性別、BMI（25未満、25以上）を説明変数とした修正ポアソン回帰モデルを適用する。調整リスク比を算出しプラセボ群と比較する。

解析項目　　：治療開始から2ヵ月及び3ヵ月の2時点連続で、けん怠感（疲労感）、息切れ又は呼吸困難感、嗅覚の異常、味覚の異常の各症状がある研究対象者の割合（COVID-19と関連がある、又は関連不明）

解析対象集団：FAS

解析方法　　：治療開始から2ヵ月及び3ヵ月の2時点連続で、研究対象者自身がCOVID-19と関連がある、又は関連不明と判断したけん怠感（疲労感）、息切れ又は呼吸困難感、嗅覚の異常、味覚の異常の各症状の有無を応答変数とし、治療薬群、COVID-19ワクチン接種歴の有無、同意取得時のCOVID-19症状（14症状）の重症度（9点未満、9点以上）、年齢（連続値）、性別、BMI（25未満、25以上）を説明変数とした修正ポアソン回帰モデルを適用する。調整リスク比を算出しプラセボ群と比較する。

解析項目　　：「治療開始から1ヵ月及び3ヵ月の2時点両方でCOVID-19の14症状（けん怠感（疲労感）、体の痛み又は筋肉痛、頭痛、悪寒、熱っぽさ、鼻水もしくは鼻づまり、喉の痛み、咳、息切れ又は呼吸困難、吐き気、嘔吐、下痢、嗅覚の異常、味覚の異常）のいずれかがある」又は「3ヵ月時点で筋力低下、集中力・思考力の低下、課題解決力の低下、物忘れ（短期又は長期）、不眠、脱毛、動悸又は心拍数の増加、関節痛、食欲不振、めまい又は平衡感覚の異常、胸痛、皮膚の発疹のいずれかの症状がある」研究対象者の割合

解析対象集団：FAS

解析方法　　：「治療開始から1ヵ月及び3ヵ月の2時点両方でCOVID-19の14症状（けん怠感（疲労感）、体の痛み又は筋肉痛、頭痛、悪寒、熱っぽさ、鼻水もしくは鼻づまり、喉の痛み、咳、息切れ又は呼吸困難、吐き気、嘔吐、下痢、嗅覚の異常、味覚の異常）のいずれかがある」又は「3ヵ月時点で筋力低下、集中力・思考力の低下、課題解決力の低下、物忘れ（短期又は長期）、不眠、脱毛、動悸又は心拍数の増加、関節痛、食欲不振、めまい又は平衡感覚の異常、胸痛、皮膚の発疹のいずれかの症状がある」のいずれかの有無を応答変数とし、治療薬群、COVID-19ワクチン接種歴の有無、同意取得時のCOVID-19症状（14症状）の重症度（9点未満、9点以上）、年齢（連続値）、性別、BMI（25未満、25以上）を説明変数とした修正ポアソン回帰モデルを適用する。調整リスク比を算出しプラセボ群と比較する。

解析項目　　：「治療開始から1ヵ月及び3ヵ月の2時点両方でCOVID-19の14症状（けん怠感（疲労感）、体の痛み又は筋肉痛、頭痛、悪寒、熱っぽさ、鼻水もしくは鼻づまり、喉の痛み、咳、息切れ又は呼吸困難、吐き気、嘔吐、下痢、嗅覚の異常、味覚の異常）のいずれかがある」又は「3ヵ月時点で筋力低下、集中力・思考力の低下、課題解決力の低下、物忘れ（短期又は長期）、不眠、脱毛、動悸又は心拍数の増加、関節痛、食欲不振、めまい又は平衡感覚の異常、胸痛、皮膚の発疹のいずれかの症状がある」研究対象者の割合（COVID-19と関連がある、又は関連不明）

解析対象集団：FAS

解析方法　　：「治療開始から1ヵ月及び3ヵ月の2時点両方で研究対象者自身がCOVID-19と関連がある、又は関連不明と判断したCOVID-19の14症状（けん怠感（疲労感）、体の痛み又は筋肉痛、頭痛、悪寒、熱っぽさ、鼻水もしくは鼻づまり、喉の痛み、咳、息切れ又は呼吸困難、吐き気、嘔吐、下痢、嗅覚の異常、味覚の異常）のいずれかがある」又は「3ヵ月時点で研究対象者自身がCOVID-19と関連がある、又は関連不明と判断した筋力低下、集中力・思考力の低下、課題解決力の低下、物忘れ（短期又は長期）、不眠、脱毛、動悸又は心拍数の増加、関節痛、食欲不振、めまい又は平衡感覚の異常、胸痛、皮膚の発疹のいずれかの症状がある」のいずれかの有無を応答変数とし、治療薬群、COVID-19ワクチン接種歴の有無、同意取得時のCOVID-19症状（14症状）の重症度（9点未満、9点以上）、年齢（連続値）、性別、BMI（25未満、25以上）を説明変数とした修正ポアソン回帰モデルを適用する。調整リスク比を算出しプラセボ群と比較する。

解析項目　　：治療開始から1ヵ月及び3ヵ月の2時点両方で COVID-19の14症状（けん怠感（疲労感）、体の痛み又は筋肉痛、頭痛、悪寒、熱っぽさ、鼻水もしくは鼻づまり、喉の痛み、咳、息切れ又は呼吸困難、吐き気、嘔吐、下痢、嗅覚の異常、味覚の異常）のいずれかの症状がある研究対象者の割合

解析対象集団：FAS

解析方法　　：治療開始から1ヵ月及び3ヵ月の2時点両方で COVID-19の14症状（けん怠感（疲労感）、体の痛み又は筋肉痛、頭痛、悪寒、熱っぽさ、鼻水もしくは鼻づまり、喉の痛み、咳、息切れ又は呼吸困難、吐き気、嘔吐、下痢、嗅覚の異常、味覚の異常）のいずれかの症状の有無を応答変数とし、治療薬群、COVID-19ワクチン接種歴の有無、同意取得時のCOVID-19症状（14症状）の重症度（9点未満、9点以上）、年齢（連続値）、性別、BMI（25未満、25以上）を説明変数とした修正ポアソン回帰モデルを適用する。調整リスク比を算出しプラセボ群と比較する。

解析項目　　：治療開始から1ヵ月及び3ヵ月の2時点両方で COVID-19の14症状（けん怠感（疲労感）、体の痛み又は筋肉痛、頭痛、悪寒、熱っぽさ、鼻水もしくは鼻づまり、喉の痛み、咳、息切れ又は呼吸困難、吐き気、嘔吐、下痢、嗅覚の異常、味覚の異常）のいずれかの症状がある研究対象者の割合（COVID-19と関連がある、又は関連不明）

解析対象集団：FAS

解析方法　　：治療開始から1ヵ月及び3ヵ月の2時点両方で研究対象者自身がCOVID-19と関連がある、又は関連不明と判断したCOVID-19の14症状（けん怠感（疲労感）、体の痛み又は筋肉痛、頭痛、悪寒、熱っぽさ、鼻水もしくは鼻づまり、喉の痛み、咳、息切れ又は呼吸困難、吐き気、嘔吐、下痢、嗅覚の異常、味覚の異常）のいずれかの症状の有無を応答変数とし、治療薬群、COVID-19ワクチン接種歴の有無、同意取得時のCOVID-19症状（14症状）の重症度（9点未満、9点以上）、年齢（連続値）、性別、BMI（25未満、25以上）を説明変数とした修正ポアソン回帰モデルを適用する。調整リスク比を算出しプラセボ群と比較する。

解析項目　　：治療開始から1ヵ月及び3ヵ月の2時点両方で、 COVID-19の14症状（けん怠感（疲労感）、体の痛み又は筋肉痛、頭痛、悪寒、熱っぽさ、鼻水もしくは鼻づまり、喉の痛み、咳、息切れ又は呼吸困難、吐き気、嘔吐、下痢、嗅覚の異常、味覚の異常）の各症状がある研究対象者の割合

解析対象集団：FAS

解析方法　　：治療開始から1ヵ月及び3ヵ月の2時点両方で、 COVID-19の14症状（けん怠感（疲労感）、体の痛み又は筋肉痛、頭痛、悪寒、熱っぽさ、鼻水もしくは鼻づまり、喉の痛み、咳、息切れ又は呼吸困難、吐き気、嘔吐、下痢、嗅覚の異常、味覚の異常）の各症状の有無を応答変数とし、治療薬群、COVID-19ワクチン接種歴の有無、同意取得時のCOVID-19症状（14症状）の重症度（9点未満、9点以上）、年齢（連続値）、性別、BMI（25未満、25以上）を説明変数とした修正ポアソン回帰モデルを適用する。調整リスク比を算出しプラセボ群と比較する。

解析項目　　：治療開始から1ヵ月及び3ヵ月の2時点両方で、 COVID-19の14症状（けん怠感（疲労感）、体の痛み又は筋肉痛、頭痛、悪寒、熱っぽさ、鼻水もしくは鼻づまり、喉の痛み、咳、息切れ又は呼吸困難、吐き気、嘔吐、下痢、嗅覚の異常、味覚の異常）の各症状がある研究対象者の割合（COVID-19と関連がある、又は関連不明）

解析対象集団：FAS

解析方法　　：治療開始から1ヵ月及び3ヵ月の2時点両方で、 研究対象者自身がCOVID-19と関連がある、又は関連不明と判断したCOVID-19の14症状（けん怠感（疲労感）、体の痛み又は筋肉痛、頭痛、悪寒、熱っぽさ、鼻水もしくは鼻づまり、喉の痛み、咳、息切れ又は呼吸困難、吐き気、嘔吐、下痢、嗅覚の異常、味覚の異常）の各症状の有無を応答変数とし、治療薬群、COVID-19ワクチン接種歴の有無、同意取得時のCOVID-19症状（14症状）の重症度（9点未満、9点以上）、年齢（連続値）、性別、BMI（25未満、25以上）を説明変数とした修正ポアソン回帰モデルを適用する。調整リスク比を算出しプラセボ群と比較する。

解析項目　　：治療開始から3ヵ月時点で筋力低下、集中力・思考力の低下、課題解決力の低下、物忘れ（短期又は長期）、不眠、脱毛、動悸又は心拍数の増加、関節痛、食欲不振、めまい又は平衡感覚の異常、胸痛、皮膚の発疹のいずれかの症状がある研究対象者の割合

解析対象集団：FAS

解析方法　　：治療開始から3ヵ月時点で筋力低下、集中力・思考力の低下、課題解決力の低下、物忘れ（短期又は長期）、不眠、脱毛、動悸又は心拍数の増加、関節痛、食欲不振、めまい又は平衡感覚の異常、胸痛、皮膚の発疹のいずれかの症状の有無を応答変数とし、治療薬群、COVID-19ワクチン接種歴の有無、同意取得時のCOVID-19症状（14症状）の重症度（9点未満、9点以上）、年齢（連続値）、性別、BMI（25未満、25以上）を説明変数とした修正ポアソン回帰モデルを適用する。調整リスク比を算出しプラセボ群と比較する。

解析項目　　：治療開始から3ヵ月時点で筋力低下、集中力・思考力の低下、課題解決力の低下、物忘れ（短期又は長期）、不眠、脱毛、動悸又は心拍数の増加、関節痛、食欲不振、めまい又は平衡感覚の異常、胸痛、皮膚の発疹のいずれかの症状がある研究対象者の割合（COVID-19と関連がある、又は関連不明）

解析対象集団：FAS

解析方法　　：治療開始から3ヵ月時点で研究対象者自身がCOVID-19と関連がある、又は関連不明と判断した筋力低下、集中力・思考力の低下、課題解決力の低下、物忘れ（短期又は長期）、不眠、脱毛、動悸又は心拍数の増加、関節痛、食欲不振、めまい又は平衡感覚の異常、胸痛、皮膚の発疹のいずれかの症状の有無を応答変数とし、治療薬群、COVID-19ワクチン接種歴の有無、同意取得時のCOVID-19症状（14症状）の重症度（9点未満、9点以上）、年齢（連続値）、性別、BMI（25未満、25以上）を説明変数とした修正ポアソン回帰モデルを適用する。調整リスク比を算出しプラセボ群と比較する。

解析項目　　：治療開始から3ヵ月時点で筋力低下、集中力・思考力の低下、課題解決力の低下、物忘れ（短期又は長期）、不眠、脱毛、動悸又は心拍数の増加、関節痛、食欲不振、めまい又は平衡感覚の異常、胸痛、皮膚の発疹の各症状がある研究対象者の割合

解析対象集団：FAS

解析方法　　：治療開始から3ヵ月時点で筋力低下、集中力・思考力の低下、課題解決力の低下、物忘れ（短期又は長期）、不眠、脱毛、動悸又は心拍数の増加、関節痛、食欲不振、めまい又は平衡感覚の異常、胸痛、皮膚の発疹の各症状の有無を応答変数とし、治療薬群、COVID-19ワクチン接種歴の有無、同意取得時のCOVID-19症状（14症状）の重症度（9点未満、9点以上）、年齢（連続値）、性別、BMI（25未満、25以上）を説明変数とした修正ポアソン回帰モデルを適用する。調整リスク比を算出しプラセボ群と比較する。

解析項目　　：治療開始から3ヵ月時点で筋力低下、集中力・思考力の低下、課題解決力の低下、物忘れ（短期又は長期）、不眠、脱毛、動悸又は心拍数の増加、関節痛、食欲不振、めまい又は平衡感覚の異常、胸痛、皮膚の発疹の各症状がある研究対象者の割合（COVID-19と関連がある、又は関連不明）

解析対象集団：FAS

解析方法　　：治療開始から3ヵ月時点で研究対象者自身がCOVID-19と関連がある、又は関連不明と判断した筋力低下、集中力・思考力の低下、課題解決力の低下、物忘れ（短期又は長期）、不眠、脱毛、動悸又は心拍数の増加、関節痛、食欲不振、めまい又は平衡感覚の異常、胸痛、皮膚の発疹の各症状の有無を応答変数とし、治療薬群、COVID-19ワクチン接種歴の有無、同意取得時のCOVID-19症状（14症状）の重症度（9点未満、9点以上）、年齢（連続値）、性別、BMI（25未満、25以上）を説明変数とした修正ポアソン回帰モデルを適用する。調整リスク比を算出しプラセボ群と比較する。

解析項目　　：治療開始から3ヵ月時点でCOVID-19 罹患前同様の通常の健康状態に戻っていない研究対象者の割合

解析対象集団：FAS

解析方法　　：治療開始から3ヵ月時点でCOVID-19 罹患前同様の通常の健康状態に戻っているかを応答変数とし、治療薬群、COVID-19ワクチン接種歴の有無、同意取得時のCOVID-19症状（14症状）の重症度（9点未満、9点以上）、年齢（連続値）、性別、BMI（25未満、25以上）を説明変数とした修正ポアソン回帰モデルを適用する。調整リスク比を算出しプラセボ群と比較する。

解析項目：治療開始から3ヵ月後及び6ヵ月後のそれぞれの時点における、 QOLのベースラインからの変化量

解析対象集団：FAS

解析方法：時点別にEQ-5D-5Lから算出される効用値のベースラインからの変化量を各群で要約する。

解析項目：治療開始から3ヵ月後及び6ヵ月後のそれぞれの時点における、労働生産性スコアのベースラインからの変化量

解析対象集団：FAS

解析方法：時点別に労働生産性スコアのベースラインからの変化量を各群で要約する。

- - 1. 有効性評価項目に対するサブグループ解析

割付因子（ワクチン接種歴、同意取得時のCOVID-19症状(14症状）の重症度（9点未満、9点以上））、年齢、性別、BMI、SARS-CoV-2感染歴でのサブグループ解析を行う。

- - 1. 安全性評価項目

解析項目　　：有害事象、疾病等

解析対象集団：SAS

解析方法　　：ICH国際医薬用語集日本語版（MedDRA）の器官別大分類（SOC）別及び基本語（PT）別に発現例数及び件数を発現群毎に集計し、発現割合を算出する。これらの発現割合の95% 信頼区間を Clopper-Pearson 法にて算出する。研究対象薬との因果関係が「関連あり」と判定された有害事象を副作用とし、有害事象と同様に集計する。さらに、発現時期、重症度、試験薬の処置及び転帰を、SOC、PT別に要約する。疾病等についても、同様に集計する。

- 1. 中間解析及び早期中止に関する基準

有効性主要評価項目の解析が可能なFAS症例数が全体で909例に到達した時点で、予想よりも明らかに有効性が優れている場合に研究を中止する目的で中間解析を1回行う。尚、中間解析時点で一定数の症例が既に登録されている場合、中間解析を実施しない可能性がある。

中間解析は本研究とは独立した統計解析担当者が行う。13.3.4項に記載した解析結果に基づき、独立データモニタリング委員会は、有効性中止又は研究の継続の勧告を行う。中間解析が行われている間も研究対象者の登録は可能とする。

中間解析に伴う多重性の調整としてO’Brien & Fleming型を用いることとする。

中間解析を実施するか否かの判断時期など中間解析の実施基準の目安や有効性中止の判断基準を、別途手順書で事前に規定し、中間解析の実施前に固定する。また、有効性中止あるいは試験継続勧告の情報開示範囲など、非盲検下での情報を厳格に管理した状態で中間解析を実施するための手順及び方法を定めた中間解析手順書を作成し、中間解析の実施前に固定する。

- 1. 統計解析計画の変更

研究代表医師は、本研究開始後に解析の変更や追加が生じた場合、その妥当性及び本研究の評価への影響を検討し、統計解析担当責任者と協議し、研究計画書を改訂し、解析計画の変更に至った経緯を本研究の総括報告書において説明する。

1. 品質管理及び品質保証
   1. 品質管理方針

本研究は臨床研究法及び同法施行規則を遵守して実施する。したがって、これらの法令が要求する事項を満たすよう品質管理方針を定める。

- 1. 品質目標

臨床研究法により要求される文書を確実に作成、保管するとともに、研究対象者の保護に関する事項を遵守する。主要評価項目が解析できる研究対象者数として20%の脱落を許容する。

- 1. モニタリング

研究代表医師は、臨床研究に対する信頼性の確保及び臨床研究の対象者の保護の観点から臨床研究が適正に行われていることを確保するため、本研究の進捗状況並びに本研究が法規則及び研究計画書に従って行われているかどうかについて、モニタリング担当者を指名し、モニタリング手順書を作成したうえでモニタリングを実施させる。

モニタリング担当者は、モニタリング手順書に従いモニタリングを実施し、その記録（モニタリング報告書）を作成する。

研究責任医師又は実施医療機関の管理者は、モニタリング担当者が、原資料を閲覧できることを保証する。

- 1. 監査

研究代表医師は、臨床研究に対する信頼性の確保及び臨床研究の対象者の保護の観点から臨床研究により収集された資料の信頼性を確保するため、本研究が法規則等及び研究計画書に従って行われたかどうかについて、監査担当者を指名し、監査手順書を作成したうえで監査を実施させる。

研究代表医師は本研究の実施に携わる者及びそのモニタリングに従事する者に監査を行わせてはならない。

監査担当者は監査手順書に従い監査を実施する。

研究責任医師又は実施医療機関の管理者は、監査担当者が、原資料を閲覧できることを保証する。

- 1. 規制当局等による調査への対応

研究責任医師又は実施医療機関の管理者は、認定臨床研究審査委員会又はその指名する者並びに厚生労働大臣又はその指名する者が調査を要求した場合にはそれを受け入れ、原資料及びその他必要な資料を閲覧できることを保証する。

- 1. 不適合
     1. 不適合の定義

不適合とは、臨床研究法施行規則、研究計画書、手順書等の不遵守及び研究データの改ざん、ねつ造等をいう。不適合を管理するにあたっては、以下の手順に基づき、対応する。

- - 1. 重大な不適合

重大な不適合とは、臨床研究の研究対象者の人権や安全性及び研究の進捗や結果の信頼性に影響を及ぼすものをいう。例えば、選択・除外基準や中止基準、併用禁止療法等の不遵守をいい、臨床研究の研究対象者の緊急の危険を回避するためその他医療上やむを得ない理由により研究計画書に従わなかったものについては含まない。

研究代表医師は、重大な不適合を知りえたときは、認定臨床研究審査委員会の意見を聴かなければならない。

- - 1. 不適合の管理手順

研究責任医師は、本研究において判明した不適合をすべて記録する。

研究分担医師は、不適合であることを知ったときは、速やかに研究責任医師に報告する。

研究責任医師は、不適合であることを知ったときは、速やかに実施医療機関の管理者に報告するとともに、研究代表医師に通知する。

研究代表医師は、不適合の発生について速やかに他の研究責任医師に情報提供するとともに、不適合の発生状況及びその後の対応について、認定臨床研究審査委員会に定期報告する。

1. 倫理的配慮
   1. 遵守すべき諸規則

本研究は、ヘルシンキ宣言に基づいた倫理原則に則り、臨床研究法、同法施行規則及びその他関連通知に従って実施する。研究責任医師及び研究分担医師は、本研究計画書を遵守してプロトコル治療を実施する。

- 1. 認定臨床研究審査委員会及び実施医療機関の管理者の承認

本研究を実施することの適否について認定臨床研究審査委員会が審査し承認を得て、実施医療機関の管理者による承認を得た後、jRCT公表後に実施する。

- 1. 本研究における研究対象者の費用負担

1. 試験薬は塩野義製薬株式会社から無償提供されるため、研究対象者の金銭的負担はない。
2. 研究対象者に対する謝礼として、オンライン面談及びスタディ・コンシェルジュへの入力の回数に応じて最大で10,000円を研究対象者が指定する口座に振り込む（登録時のオンライン面談、1週後のオンライン面談を実施した場合はそれぞれ2,000円、1ヵ月後、3ヵ月後、6ヵ月後の入力を実施した場合にはそれぞれ1,500円。2ヵ月後、4ヵ月後、5ヵ月後の入力を実施した場合はそれぞれ500円）。
   1. 同意説明文書及び研究対象者の同意
      1. 同意取得手順

研究責任医師は、研究対象者の同意を得るに際し、同意説明文書を作成し、認定臨床研究審査委員会の承認を得る。

研究責任医師又は研究分担医師は、本研究に関して認定臨床研究審査委員会で承認された同意説明文書の内容をオンライン面談で研究対象者に説明し、研究対象者が本研究の内容を十分に理解したことを確認したうえで、本研究への参加を依頼する。

研究対象者が同意した場合には、研究対象者のスマートフォンでMIROHAのeConsent機能を用いて同意書に研究対象者本人による電子署名を取得する。

研究対象者が電子署名したことを確認後、研究責任医師又は研究分担医師は、MIROHAのeConsent機能を用いて同意書に電子署名する。研究責任医師又は研究分担医師は、両者が電子署名をしたことを確認した後、同意説明文書及び同意書のpdfファイルをダウンロードできるリンクをメールで研究対象者本人に連絡するとともに、同意書のpdfファイルを保管する。なお、MiROHAに保管された電子署名済の同意書を原本とする。

ただし、説明を受けた研究対象者が研究参加に同意したもののMiROHAのeConsent機能での電子署名が困難な場合には、研究対象者による同意説明文書及び署名前の同意書を閲覧した記録がMiROHAに載っていることを確認したうえで研究対象者が署名及び署名日を記入した書面（様式は問わない）をオンライン面談時に確認後、研究責任医師又は研究分担医師が同意書に署名することで文書同意を取得できたものとする。なお、この場合においては、後日、研究対象者に説明を行った研究責任医師又は研究分担医師が署名した同意書（紙媒体）を送付し、その同意書に研究対象者が署名の上で返送したものを原本として保管する。

研究対象者は、本研究の参加について同意した後であっても、いつでも研究対象者の自由意志で同意を撤回することができる。同意を撤回する場合には、研究対象者は電話や電子メール等で研究責任医師又は研究分担医師に連絡する。研究責任医師又は研究分担医師はMIROHAに同意撤回の旨を記録する。

研究責任医師又は研究分担医師は、すでに同意を取得している研究対象者の研究参加の意思に影響を与えるような情報に関して同意説明文書を改訂した場合は、認定臨床研究審査委員会で審査を受け承認された同意説明文書（改訂版）を用いて、研究対象者に説明し再同意取得をする。

- - 1. 同意説明文書に記載すべき事項

同意説明文書には、以下の内容を含むものとする。

1. 研究の名称及び当該研究の実施について実施医療機関の管理者の承認を受けていること及び厚生労働大臣に実施計画を提出していること
2. 実施医療機関の名称及び研究責任医師の氏名及び職名（共同研究機関の名称及び研究責任医師の氏名を含む）
3. 本研究の目的及び意義
4. 医薬品等の概要
5. 本研究の方法（研究対象者から取得された情報の利用目的を含む）及び期間
6. 研究対象者として選定された理由
7. 研究対象者に生じる負担並びに予測されるリスク及び利益
8. 本研究が実施又は継続されることに同意した場合であっても随時これを撤回できる旨
9. 本研究が実施又は継続されることに同意しないこと又は同意を撤回することによって研究対象者等が不利益な取扱いを受けない旨
10. 本研究に関する情報公開の方法
11. 研究対象者等の求めに応じて、他の研究対象者等の個人情報等の保護及び当該研究の独創性の確保に支障がない範囲内で研究計画書及び本研究の方法に関する資料を入手又は閲覧できる旨並びにその入手又は閲覧の方法
12. 個人情報等の取扱い（特定の個人を識別することができないようにする場合にはその方法を含む）
13. 情報の保管及び廃棄の方法
14. 本研究の資金源等、研究機関の研究に係る利益相反及び個人の収益等、研究者等の研究に係る利益相反に関する状況
15. 研究対象者等及びその関係者からの相談等、苦情及び問合せへの対応
16. 研究対象者等への経済的負担又は謝礼について
17. 他の治療方法の有無及び内容並びに他の治療法により予期される利益及び不利益に関する事項
18. 本研究によって生じた健康被害に対する補償の有無及びその内容
19. 特定臨床研究の審査意見業務を行う認定臨床研究審査委員会における審査事項その他当該特定臨床研究に係る認定臨床研究審査委員会に関する事項
20. 臨床研究法第三十二条に規定する契約の内容
21. その他特定臨床研究の実施に関し必要な事項
    1. 研究対象者からの相談窓口

研究対象者及びその関係者からの質問は、研究責任医師又は研究分担医師が対応する。

- 1. 研究対象者の予想される利益及び不利益
     1. 予想される利益

いずれの研究対象者でも、必要に応じてCOVID-19の症状に対する対症療法を実施するため、通常診療と同程度の治療上の利益が得られる。本研究計画書に従って罹患後症状、QOL、労働生産性の情報を収集するため、研究対象者は、通常診療よりも詳細な健康状態等を把握することができる。

また、本研究において、COVID-19 に対するエンシトレルビル フマル酸の治療効果が詳細に検討されることによって、将来的に、より最適な治療法の選択について評価できる可能性がある。

- - 1. 予想される不利益

スマートフォンにインストールしたアプリに罹患後症状、QOL、労働生産性についての回答を入力するため、通常診療に比べて研究対象者の身体的及び精神的負担が増える可能性がある。

エンシトレルビルの服用により、10.6 項に示す副作用が発現する可能性がある。

- - 1. 利益と不利益の総合評価及び不利益を最小化する対策

エンシトレルビルの副作用は一過性であり、重篤な副作用は報告されておらず、不利益が利益を上回る可能性は低い。また、不利益を最小限にするため、服用終了後にオンライン面談に基づく安全性確認を実施し、有害事象等の兆候を調査する。なお、本研究に参加する前に研究対象者に十分に説明したうえで、その意思を確認する。研究責任医師及び共同研究者である塩野義製薬株式会社は、安全性に関する情報を収集し、必要に応じて研究計画書を改訂するなど適切に対応する。

- 1. 研究対象者の秘密保持（個人情報の保護）

開発業務受託機関は、受託業務の実施のために研究対象者募集サイトに患者が入力した個人情報（氏名、携帯電話番号、電子メールアドレス、住所等）を閲覧するため、その旨を患者が研究対象者募集サイトに情報を入力する際に説明し、同意を得る。開発業務受託機関は、患者の個人情報が外部に漏洩することがないよう保管管理を厳重に行う。

本研究の実施に係る生データ類及び同意書等を取扱う際は、研究対象者の秘密保護に十分配慮する。同意を取得した研究対象者に対して研究対象者識別コードを付与する。研究対象者識別コードは、イニシャルやカルテID等のような特定の個人を識別できる情報とは無関係の数字記号等で構成され、症例報告書等の本研究に関する書類を作成する際には研究対象者識別コードを使用することで特定の個人を識別することができないようにする。研究責任医師は、研究対象者の氏名等の個人情報が外部に漏洩することがないよう対応表等の保管管理を厳重に行う。研究の結果を公表する際は、研究対象者を特定できる情報を含まないようにする。

1. 健康被害に対する補償

研究責任医師及び研究分担医師は、本研究の実施に起因して研究対象者に健康被害が発生した場合には、研究対象者がただちに適切な診断、治療及び必要な措置を受けることができるよう近医への受診勧告その他必要な措置を講じる。

研究代表医師は、研究対象者の健康被害を補償するため、以下の補償内容を有する臨床研究保険（補償保険）に加入し、臨床研究保険（補償保険）の支払条件に従って補償を行う。

（１） 研究対象者の死亡又は後遺障害に対する補償金

（２） 研究対象者の健康被害の治療のために要する医療費・医療手当。ただし、この場合の治療には健康保険を適用し、補償は研究対象者の治療費の自己負担額及び治療費以外の費用負担に対する医療手当とする。

研究責任医師又は研究分担医師は、同意説明文書に健康被害発生時には補償が受けられる旨を記載し、また、補償の概要を示した文書を作成し、同意説明時に研究対象者へ提供する。

1. 臨床研究全体の中止又は終了
   1. 中止の基準

研究代表医師は、下記に該当する場合、必要に応じて研究を中止又は中断する。

1. 予想される有害事象（疾病等）が計画時の想定を著しく超える場合等、研究対象者の安全性又は本研究の実施に悪影響を及ぼす可能性のある新たな重大な情報を入手した場合
2. 研究対象者の登録が計画と比較して著しく遅い場合等、目標とする研究対象者数を達成することが極めて困難であると判断される場合
3. 認定臨床研究審査委員会から本研究を中止すべき旨の意見を受けた場合
4. その他、本研究の中止又は中断を必要とする状況が発生した場合
   1. 中止の手順

研究代表医師は、中止基準に該当することが判明し、本研究全体の中止を決定した場合、速やかに研究責任医師に通知する。

研究責任医師又は研究分担医師は、研究対象者に研究中止について説明し、可能な範囲で本研究において計画されている観察・検査・評価を実施するとともに必要に応じて代替治療等の医療上の処置を講じる。

研究代表医師は、本研究全体の中止を決定した日から10日以内に認定臨床研究審査委員会に中止通知書を提出するとともに、厚生労働大臣に届け出る。

- 1. 終了の基準

研究代表医師が、総括報告書の概要をjRCTに記録することにより公表した日を本研究が終了した日とする。

1. 研究の情報公開及び結果公表
   1. 研究の登録

本研究の実施に先立ち、jRCTに登録する。研究計画書の変更及び研究の進捗に応じて適宜更新する。

- 1. 研究結果の公表
     1. 主要評価項目報告書

研究代表医師は、主要評価項目に係るデータの収集を行うための期間が終了した日から原則として１年以内に主要評価項目報告書を作成し、認定臨床研究審査委員会に意見を聴き、実施医療機関の管理者に提出する。認定臨床研究審査委員会が意見を述べた日から起算して1月以内にjRCTに記録することで公表する。

研究代表医師は公表したことを速やかに実施医療機関の管理者に報告するとともに、その旨を他の研究責任医師に情報提供する。他の研究責任医師は、速やかに、当該情報提供の内容を実施医療機関の管理者に報告する。

なお、本研究では主要評価項目報告書の作成が総括報告書作成と同時期になる予定であるため、総括報告書の作成により主要評価項目報告書を作成したものとみなす。

- - 1. 総括報告書

研究代表医師は、すべての評価項目に係るデータの収集を行うための期間が終了した日から原則として１年以内に総括報告書及びその概要を作成する。

総括報告書には少なくとも以下の事項を含めて作成する。

（１）臨床研究の対象者の背景情報（年齢、性別等）

（２）臨床研究のデザインに応じた進行状況に関する情報（対象者数の推移等）

（３）疾病等の発生状況のまとめ

（４）主要評価項目及び副次評価項目のデータ解析及び結果

研究代表医師は、総括報告書及びその概要について認定臨床研究審査委員会に意見を聴き、実施医療機関の管理者に提出する。認定臨床研究審査委員会が意見を述べた日から起算して1月以内に、当該総括報告書の概要、研究計画書及び統計解析計画書（作成した場合）をjRCTに記録することで公表する。

研究代表医師は公表したことを速やかに実施医療機関の管理者に報告するとともに、その旨を他の研究責任医師に情報提供する。他の研究責任医師は、速やかに、当該情報提供の内容を実施医療機関の管理者に報告する。

- - 1. 学会等の公表

本研究から得られた成果について、速やかに学会発表や論文投稿による公表を行う。公表する際は、研究対象者等及びその関係者の人権や権利利益の保護のために必要な措置を講じた上で公表する。学会発表者及び論文著者は、本研究への貢献度に基づき決定する。

1. 変更管理
   1. 認定臨床研究審査委員会で承認された書類の変更

認定臨床研究審査委員会で承認された書類から変更が生じた場合は、認定臨床研究審査委員会に変更申請する。認定臨床研究審査委員会から意見が述べられた場合には、速やかに、その意見の内容について実施医療機関の管理者に報告する。

- 1. 実施計画の変更

研究代表医師は、実施計画を変更（厚生労働省令で定める軽微な変更を除く。）するときは、当該実施計画に記載されている認定臨床研究審査委員会の意見を聴き、あらかじめ変更後の実施計画及び様式第二による届書を提出して行う。

- 1. 実施計画の軽微な変更

研究代表医師は、実施計画について、臨床研究法施行規則第四十二条に則り、軽微な変更をしたときは、その変更の日から 10 日以内に、その内容を、当該実施計画に記載されている認定臨床研究審査委員会に通知するとともに、厚生労働大臣に届け出る。

1. 利益相反
   1. 本研究に関する資金源

塩野義製薬株式会社の資金提供を受けて実施する。

本研究は医学的な視点から行われ、当該企業の利益や便宜をはかるものではない。

- 1. 利益相反管理

本研究は、各々の実施医療機関において事実確認を受けた上で利益相反管理計画を作成し、認定臨床研究審査委員会の意見を聴き適切な管理を行う。

本研究の利益相反状況は別紙1のとおり。

1. 認定臨床研究審査委員会、厚生労働大臣に対する定期報告

研究代表医師は、特定臨床研究の実施状況について、次に掲げる事項について、実施医療機関の管理者に報告した上で、認定臨床研究審査委員会、厚生労働大臣に報告する。

研究代表医師は認定臨床研究審査委員会に定期報告を行ったことを速やかに他の研究責任医師に情報提供する。他の研究責任医師は、速やかに、当該情報提供の内容を実施医療機関の管理者に報告する。

- 1. 認定臨床研究審査委員会に対する定期報告
     1. 定期報告における報告事項

（１）本研究に参加した研究対象者の数

（２）本研究に係る疾病等の発生状況及びその後の経過

（３）本研究に係るこの省令又は研究計画書に対する不適合の発生状況及びその後の対応

（４）本研究の安全性及び科学的妥当性についての評価

（５）本研究に対する医薬品等製造販売業者等の関与に関する事項

- - 1. 定期報告の時期

認定臨床研究審査委員会への定期報告は、原則として、実施計画を厚生労働大臣に提出した日から起算して、１年ごとに、当該期間満了後２月以内に行う。

- 1. 厚生労働大臣に対する定期報告
     1. 定期報告における報告事項

（１）実施計画に記載されている認定臨床研究審査委員会の名称

（２）認定臨床研究審査委員会による本研究の継続の適否

（３）本研究に参加した研究対象者の数

- - 1. 定期報告の時期

厚生労働大臣への定期報告は、認定臨床研究審査委員会が意見を述べた日から起算して、１月以内に行う。

1. 資料及び記録等の保管並びに廃棄方法
   1. 原資料の保管

「原資料」とは、研究対象者に対する医薬品等の適用及び診療により得られた臨床所見、観察その他の活動に関する元の記録やデータをいう。

研究責任医師又は実施医療機関の管理者は、認定臨床研究審査委員会及び規制当局又はその指名する者による調査又は監査のため、原資料及び本研究固有の文書を含む以下の資料を保管する。

これらの資料には、署名・日付入り同意書pdfファイル等が該当する。

また、研究責任医師又は実施医療機関の管理者は、保管すべき必須文書を、本研究の中止又は終了後5年が経過した日までの期間、保存する。

研究責任医師は上記の文書、記録等の修正を行う場合は、修正者の氏名及び修正を行った年月日を記録し、修正した記録とともに保存する。

- 1. 法で定める記録文書の保管

研究責任医師は、本研究の審議に関する記録及び資料等は、漏えい、混交、盗難、紛失等が起こらないよう、本研究の中止又は終了後5年が経過した日までの期間、適切に保管する。本研究で保管する文書は下記のとおり。

（１）研究対象者を特定する事項を記載した文書

（２）研究対象者に対する診療及び検査に関する事項を記載した文書

（３）本研究への参加に関する事項を記載した文書

（４）研究対象者への研究対象とした医薬品等の投与に関する事項を記載した文書

（５）認定臨床研究審査委員会から受領した本研究に関する審査意見業務に係る事項を記載した文書

（６）研究計画書、実施計画、研究対象者に対する説明及びその同意に係る文書

（７）総括報告書、その他の臨床研究法施行規則の規定により研究責任医師が作成した文書（又は写）

（８）モニタリング及び監査に関する文書

（９）上記（１）～（４）を除く原資料等

（10）本研究の実施に係る契約書

（11）本研究に用いる医薬品等の概要を記載した文書

（12）本研究の対象とした医薬品等を入手した記録（数量及び年月日）

（13）本研究の対象とした医薬品等を処分した記録（処分した場合）

（14）上記のほか本研究を実施するために必要な文書

なお、研究責任医師は、これらの記録の修正をする場合は、修正者の氏名及び修正した年月日を記録し、修正した記録とともに保存する。

- 1. 情報の二次利用について

本研究で得られた情報を異なる研究目的で使用する可能性がある（二次利用）。その旨を同意・説明文書に記載し、研究対象者に説明したうえで同意を取得する。二次利用を行う場合には、新たに研究計画書を作成し、当該研究に意見を聴くべき倫理審査委員会で承認を得た後に実施する。

- 1. 廃棄の手順及び方法

研究責任医師は、本研究において研究対象者から取得した情報、本研究の審議に関する記録及び資料を廃棄する場合は、特定の個人を識別することができないように必要な措置をとる。

実施医療機関の管理者は、研究責任医師から保存すべき情報、記録及び資料等について、その保存の必要がなくなったことを通知されるまで保存する。

1. 研究結果の帰属

本研究の結果の帰属については、資金提供者である塩野義製薬株式会社と大阪大学との契約に定める。本研究の終了後、資金提供者である塩野義製薬株式会社に対して臨床研究完了報告書を提出する。また、本研究で得られた情報を研究代表医師が公表する場合は、契約に従って塩野義製薬株式会社の承諾を得るものとする。

1. 本研究で設置する委員会等
   1. 独立データモニタリング委員会

本研究の中間解析結果に伴う有効性中止の要否を評価するために独立データモニタリング委員会を設置する。

独立データモニタリング委員は、本研究の領域における研究歴や業績、類似薬の開発経験、本臨床研究の実施関係者（研究責任医師等本研究に従事する者、認定臨床研究審査委員、実施医療機関の管理者等）との独立性を確保できること等を条件に選任される。

1. 引用文献

| [1] | 厚生労働科学特別研究事業　福永班, “新型コロナウイルス感染症（COVID-19）の長期合併症の実態把握と病態生理解明に向けた基盤研究　総括研究報告,” 1 6 2022. [オンライン]. Available: https://www.mhlw.go.jp/content/10900000/000945990.pdf. [アクセス日: 30 5 2023]. |
| --- | --- |
| [2] | 厚生労働科学特別研究事業　横山班, “COVID-19後遺障害に関する実態調査（中等症以上対象）　総括研究報告,” 1 6 2022. [オンライン]. Available: https://www.mhlw.go.jp/content/10900000/000945990.pdf. [アクセス日: 30 5 2023]. |
| [3] | 大阪大学大学院医学系研究科感染制御医学講座. 他, “コロナ後遺症調査結果,” 14 12 2022. [オンライン]. Available: https://www.city.toyonaka.osaka.jp/kenko/covid19_support/koronakouisyousien.files/tyousakekka3.pdf. [アクセス日: 30 5 2023]. |
| [4] | 厚生労働省, “第２回献血時の検査用検体の残余血液を用いた新型コロナウイルスの抗体保有率実態調査,” [オンライン]. Available: https://www.mhlw.go.jp/content/10906000/001070846.pdf. [アクセス日: 30 5 2023]. |
| [5] | Xie Y, et al., “Association of treatment with nirmatrelvir and the risk of post-COVID-19 condition,” JAMA Intern Med. 2023;e230743. doi: 10.1001/jamainternmed.2023.0743. |
| [6] | 迎寛, 他, “エンシトレルビル フマル酸によるCOVID-19罹患後症状（Long COVID）抑制の可能性,” Precision Medicine. 2023;6:291-299, 2023. |
| [7] | Tsampasian V, et al., “Risk factors associated with post-COVID-19 condition: A systematic review and meta-analysis,” JAMA Intern Med. 2023;e230750. doi: 10.1001/jamainternmed.2023.0750. |

**別紙1（利益相反事項）**

研究名称：エンシトレルビル フマル酸のCOVID-19罹患後症状に対する有効性の検証

研究代表医師：大阪大学医学部附属病院　感染制御部　忽那　賢志

本研究に関与する製薬企業等についてのCOI（研究に対する関与）

| 塩野義製薬株式会社とのCOIについて |
| --- |
| 研究資金等の提供  物品の提供：試験薬  役務提供：共同研究の契約に基づく研究計画書等各種ドキュメント作成への必要な情報の提供と立案等の業務 |

本研究に関与する対象薬剤製薬企業等と実施医療機関の研究責任医師・研究分担医師との開示すべきCOI

| 塩野義製薬株式会社とのCOIについて | |
| --- | --- |
| 実施医療機関名 | 人数 |
| 大阪大学医学部附属病院 | 8 |

　　　　　　　　　　　　　　　　　　　　　　　　　　（2025年4月28日時点）
