# Supplementary material for: Research to evaluate safety and impact of long COVID intervention with Ensitrelvir for National Cohort (RESILIENCE Study): A protocol for a randomized, double-blind, placebo-controlled trial
Source: PLoS One. 2025 Nov 3;20(11):e0335609. doi: 10.1371/journal.pone.0335609 (PMC12582476; doi:10.1371/journal.pone.0335609)
Supplement: Attachment — Submitted filename: Response to Reviewers.docx [file pone.0335609.s005.docx]

Title: Response to the Editor and Reviewers

Manuscript ID: PONE-D-25-15689

Manuscript Title: “Research to Evaluate Safety and Impact of Long COVID Intervention with Ensitrelvir for National Cohort (RESILIENCE Study): A protocol for a randomized, double-blind, placebo-controlled trial”

Dear Dr. Kamal Sharma,

Thank you for the opportunity to revise our manuscript.

First, we would like to sincerely apologize for the delay in our resubmission. This was due to an essential amendment to the study protocol, the details of which have now been incorporated into the revised manuscript.

We are very grateful to you and the reviewers for the insightful and constructive comments, which have helped us significantly improve the quality of our paper. We have addressed all points raised in the detailed point-by-point response below. In addition to the specific revisions requested by the reviewers, we have also performed a thorough review of the entire manuscript and made minor corrections to grammar and phrasing to enhance overall clarity and readability.

All modifications in the revised manuscript have been highlighted in yellow for your convenience.

We hope that the revised manuscript is now suitable for publication in PLOS ONE.

Sincerely,

Keiji Konishi, MD

Journal Requirements

1. Comment: Please ensure that your manuscript meets PLOS ONE's style requirements, including those for file naming.

Response: Thank you for the guidance. We have carefully reviewed the PLOS ONE style templates and revised our manuscript, including file names, to ensure full compliance with the journal's requirements.

2. Comment: Please state what role the funders took in the study.

Response: Thank you for the comment. To clarify the funder's role and resolve a previous inconsistency, we have revised both the Funding and the Competing interests sections of the manuscript.

3. Comment: Please confirm that this does not alter your adherence to all PLOS ONE policies on sharing data and materials, by including the following statement: "This does not alter our adherence to PLOS ONE policies on sharing data and materials.”

Response: As requested, we have added the statement "This does not alter our adherence to PLOS ONE policies on sharing data and materials" to the Competing interests section.

4. Comment: Your ethics statement should only appear in the Methods section of your manuscript. If your ethics statement is written in any section besides the Methods, please move it to the Methods section and delete it from any other section.

Response: Thank you for this instruction. As requested, we have moved the ethics statement regarding the institutional review board approval to the main Methods section, under the "Study design and setting" subsection. The original section has been renamed to "Dissemination" to accurately reflect its remaining content.

5. Comment: Please include captions for your Supporting Information files at the end of your manuscript...

Response: Our manuscript does not include any Supporting Information files at this time.

Reviewer #1

1. Comment: The effect size used for the sample size calculation needs to be justified.

Response: Thank you for this important point. We have clarified the basis for our sample size calculation in the manuscript. The assumed 14.6% event rate in the placebo group was based on an exploratory analysis of data from the preceding SCORPIO-SR trial. The 30% relative risk reduction (i.e., a relative risk of 0.70) was subsequently established as a clinically meaningful and plausible target for the intervention effect, informed by the potential suggested in the prior trial. This has been clarified in the Sample size section.

2. Comment: For a large sample size, a 10% dropout rate may be too optimistic.

Response: We appreciate the reviewer's valid concern. We believe the 10% rate is a reasonable initial estimate due to the participant-friendly decentralized clinical trial (DCT) design, which is expected to enhance retention by minimizing participant burden. However, we acknowledge this may be optimistic and, as the reviewer implies, a higher-than-expected dropout rate is a potential limitation. We have ensured this point is clearly stated in the Discussion section of the manuscript.

3. Comment: The sample size was calculated using a chi-square test. Why is the primary analysis planned with a Poisson model instead?

Response: This is an excellent question. While a straightforward chi-square test was used for the sample size calculation, we chose a modified Poisson regression model for the primary analysis. This approach allows for the estimation of relative risks while robustly adjusting for stratification factors and key baseline covariates, thereby providing a more precise and powerful estimate of the treatment effect. We have added a sentence to the Statistical methods section to clarify this rationale.

4. Comment: Please specify the stratification factors and covariates. Will stratification be used in randomization?

Response: Thank you for the suggestion. We have revised the manuscript to explicitly state that randomization will be stratified by COVID-19 vaccination history (yes/no) and baseline severity score of 14 COVID-19 symptoms (less than 9 vs. 9 or more). We have also specified in the Statistical methods section that the primary analysis model will adjust for these stratification factors and the baseline covariates of age, sex, and Body Mass Index (BMI).

5. Comment: Additional details are needed regarding the subgroup and sensitivity analyses.

Response: We have expanded the Statistical methods section to provide these details. Subgroup analyses for the primary endpoint will be performed based on the stratification factors, as well as age, sex, BMI, history of prior COVID-19 infection, and time from symptom onset to treatment.

6. Comment: What is the objective of the interim analysis? Is any adjustment for multiplicity?

Response: We have clarified in the Data monitoring section that the objective of the single interim analysis is to stop the trial early for overwhelming efficacy. To control the overall type I error rate, we will use the O'Brien-Fleming alpha-spending function to adjust for multiplicity.

7. Comment: Define the Full Analysis Set (FAS) and Safety Set clearly.

Response: Thank you. We have added clear definitions for the Full Analysis Set (FAS) and the Safety Analysis Set (SAS) to the Statistical methods section.

8. Comment: What methods will be used for imputing missing data?

Response: We have clarified in the Statistical methods section that the primary analysis will be based on available cases without imputation.

9. Comment: What decision will DMC make for efficacy?

Response: We have clarified in the Data monitoring section that the independent Data Monitoring Committee (DMC) will review the unblinded interim analysis results and recommend to the trial steering committee whether to continue or terminate the trial based on the pre-specified statistical stopping boundary for overwhelming efficacy.

10. Comment: Line 275: Please clarify why the sample size is n = 909 for the interim analysis.

Response: We have clarified this point in the Data monitoring section. The interim analysis will be conducted when 909 participants have been enrolled in the Full Analysis Set (FAS). This number corresponds to approximately 50% of the total required statistical information for the final analysis, not 50% of the participants.

Reviewer #2

1. Comment: The protocol has been submitted now for publication, but study status shows enrolment begun on Feb. 20, 2020 (https://jrct.mhlw.go.jp/en-latest-detail/jRCTs051230184). Authors are encouraged to provide actual status and ethical approval etc.

Response: Thank you for your review and for spotting the typo in your comment. The trial began in February 2024, not 2020, which is consistent with the clinical trial registry (jRCT) and the manuscript. Participant recruitment is currently proceeding as planned.

2. Comment: Since study is on-going, was there any interim data published.

Response: As the study is ongoing and remains blinded to investigators and participants, no interim data have been published.

3. Comment: Proposed Mechanism of action of Ensitrelvir (figure is preferred), a brief write-up of prior phase I / II studies of Ensitrelvir with outcome should be helpful for readers.

Response: Thank you for this valuable suggestion. We have significantly expanded the Introduction section to include details on ensitrelvir's mechanism of action (as a 3C-like protease inhibitor) and a summary of key findings from prior Phase I and II/III trials regarding its safety, tolerability, and efficacy on viral load and acute symptoms.

4. Comment: Reference 4 and 14 are same.

Response: We sincerely thank you for identifying this error. We have removed the duplicate reference, renumbered the entire reference list accordingly, and double-checked all citations in the text.
